# SIRT5 promote malignant advancement of chordoma by regulating the desuccinylation of c-myc

**DOI:** 10.1186/s12885-024-12140-w

**Published:** 2024-03-26

**Authors:** Minghui Jiang, Zheng Huang, Li Chen, Ting Deng, Junpeng Liu, Yue Wu

**Affiliations:** 1 Department of Orthopedics, ChangSha Third Hospital, ChangSha, China; 2 Department of Orthopedics, HuaZhong University of Science and Technology Union Shenzhen Hospital, Shenzhen, China; 3 Department of Orthopedics, BeiJing ChaoYang Hospital, Beijing, China; 4 Department of Orthopedics, BeiJing ChaoYang Hospital, BeiJing Chao-Yang Hospital, No.8 Gongti South Rd, Chaoyang District, 100020 Beijing, China

**Keywords:** Chordoma, SIRT5, Desuccinylation, c-myc, Proliferation, Migration

## Abstract

Chordoma is a relatively rare and locally aggressive malignant tumor. Sirtuin (SIRT)5 plays pivotal roles in various tumors, but the role of SIRT5 in chordoma has not been found. This study was performed to investigate the regulatory effects of SIRT5 on cell proliferation, migration, and invasion and the underlying mechanism in chordoma. A xenograft tumor mouse model was established to assess tumor growth. Reverse transcription-quantitative polymerase chain reaction was used to analyze the mRNA levels of SIRT5 and c-myc. The effects of SIRT5 and c-myc on cell proliferation, migration, and invasion of chordoma cells were detected by cell counting kit-8, colony formation, and Transwell assays. The interaction between SIRT5 and c-myc was evaluated by co-immunoprecipitation (IP) assay. The succinylation of c-myc was analyzed by IP and Western blot. The results showed that SIRT5 expression was upregulated in chordoma tissues and cells. SIRT5 interacted with c-myc to inhibit the succinylation of c-myc at K369 site in human embryonic kidney (HEK)-293T cells. Silencing of SIRT5 suppressed the cell proliferation, migration, and invasion of chordoma cells, while the results were reversed after c-myc overexpression. Moreover, silencing SIRT5 suppressed tumor growth in mice. These findings suggested that SIRT5 promoted the malignant advancement of chordoma by regulating the desuccinylation of c-myc.

**Supplementary Information:**

The online version contains supplementary material available at 10.1186/s12885-024-12140-w.

## Introduction

Chordoma, accounts for 1–4% of all bone tumors, is a primary, slowly growing, and locally aggressive neoplasms bone cancer that mainly affects the axial skeleton [1, 2]. Spinal residue, myelitis infection, and heredity are the main risk factors for chordoma induction. Chordoma can affect multiple sites of the axial skeleton, with 32% occurring inside the skull, 29.2% in the sacrum, and 32.8% affecting the rest of the spine [3]. Regrettably, about 30–40% of chordoma patients develop multi-organ (including liver, lung, and lymph nodes) metastasis [4]. For the treatment, chordoma is not sensitive to chemotherapy and is currently mainly treated with surgical resection and radiotherapy [5]. However, due to the complex and delicate structure of the central nervous system, appropriate surgical removal in clinical is difficult [6, 7]. Therefore, the prognosis for patients with chordoma remains poor, with more than 40% of patients experiencing postoperative recurrence [8]. Thus, molecular mechanisms and novel treatments for chordoma are worth further exploration.

The discovery of succinylation of lysine originates from the studies on acetylation [9]. In 2011, Zhang et al. identify succinylation modification as a new post-translational modification (PTM) [10]. Over the next decade, succinylation modification was studied extensively on different diseases. Nowadays, succinylation is considered as a PTM widely present in prokaryotes and eukaryotes, and plays vital roles in regulating various physiological or pathological functions including signaling pathways, mitochondrial metabolism, and energy metabolism [11]. Succinylation works by regulating the structure of the protein by transferring the succinyl group (-CO-CH_2_-CH_2_-CO_2_H) to the residue of the target protein [12]. Interestingly, the effects of succinylation on the structure and function of target proteins may be greater than that of other PTMs [12]. Previous studies have demonstrated the role of succinylation in various diseases, including cancers [13–15]. In addition, the degree of succinylation of certain proteins is regulated by the desuccinylation process. Sirtuin (SIRT)5 is the only known desuccinylase, located in the mitochondria. SIRT5 relies on nicotine adenine dinucleotide (NAD)^+^ Lys deacetylases to regulate key mammalian biological processes. SIRT5-mediated desuccinylation modification has been shown to be involved in the progression of many diseases [15, 16]. However, the role of SIRT5 in chordoma has not been found.

Given this background, this study aimed to explore the effects of SIRT5 on cell proliferation, migration, and invasion and the underlying mechanism in chordoma, which might provide a potential therapeutic intervention strategy for chordoma.

## Methods and materials

### Bioinformatics analysis

The Search Tool for Recurring Instances of Neighbouring Genes (STRING) database (https://cn.string-db.org/) was used to analyze SIRT5-related proteins. Besides, the GPSuc database (http://kurata14.bio.kyutech.ac.jp/GPSuc/index.php) was used to screen succinylation sites of c-myc.

### Sample collection

This study was approved by BeiJing ChaoYang Hospital. The study included 26 patients diagnosed with chordoma. The complete clinical data of chordoma patients, including age, sex, tumor location, tumor size, whether they were primary tumor, and whether the tumor was totally resected were collected in Table 1. The tumor tissue specimens and corresponding adjacent ones (about 2 cm away from the tumor margin) were collected and stored in liquid nitrogen for use. All subjects consented to clinical examination and sampling. Written informed consent was obtained from all subjects.

**Table 1:** Association between SIRT5 expression and the clinicopathologic characteristics of patients with chordoma. **SIRT**, sirtuin

Variables	Number	SIRT5 Expression		
		High (*N* = 13)	Low (*N* = 13)	P value
Age (years)				0.4201
> 50	10	4	6	
≤ 50	16	9	7	
Sex				0.2162
Male	17	7	10	
Female	9	6	3	
Tumor location				0.1847
Skull base	7	5	2	
Spine	19	8	11	
Tumor size				0.6914
≤ 3 cm	11	6	5	
> 3 cm	15	7	8	
Primary tumor				0.0271
Yes	19	12	7	
No	7	1	6	
Total resection				0.6188
Yes	5	2	3	
No	21	11	10	

### Cell culture

Chordoma cell lines (U-CH1 and U-CH2), human embryonic kidney (HEK)-293T cells, and human umbilical vein endothelial cells (HUVEC) were purchased from American type culture collection (Manassas, VA, USA). U-CH1 and U-CH2 cells were cultured in Iscove’s modified Dulbecco’s medium (IMDM)/Roswell Park Memorial Institute (RPMI) (4:1; Gibco; Thermo Fisher Scientific, Inc., Waltham, MA, USA) medium supplemented with 10% heat inactivated fetal bovine serum (FBS, Gibco), 100 U/mL penicillin and 100 µg/mL streptomycin [17]. HEK-293T and HUVEC cells were maintained in Dulbecco’s modified eagle medium (DMEM, Thermo Fisher) containing 10% FBS and 1% penicillin/streptomycin. All cells were incubated in a humidified incubator at 37 °C with 5% CO_2_.

### Cell transfection

SIRT5 short hairpin (sh) RNA (sh-SIRT5), negative control shRNA (sh-NC), negative control pcDNA 3.1 vector, and pcDNA 3.1-c-myc overexpression vector were synthesized by GenesScript Biotechnology Co. Ltd. (Nanjing, China). The cells (5 × 10^5^ cells/well) were inoculated in a 6-well plate (Corning, NY, USA) a few days before transfection. After the cell confluence reached about 60–80%, transfection was performed using Lipofectamine 3000 (Thermo Fisher) for 48 h. Next, reverse transcription-quantitative polymerase chain reaction (RT-qPCR) was performed to analyze the expression of SIRT5 and c-myc.

In addition, arginine (R) mutations were introduced at K369 (K369R), K385 (K385R) and K411 (K411R) sites of c-myc, respectively (Genscript). Then, c-myc-K369R, c-myc-K385R, and c-myc-K411R were transfected into HEK-293T cells for 24 h.

### Animal study

Animal experiment protocols in this study were approved by the Animal Ethics Committee of BeiJing ChaoYang Hospital according to the Guide for the Care and Use of Laboratory Animals (National Research Council). Twelve male BALB/c nude mice (8 weeks old) with an average weight of 25 g purchased from Oricell Biosciences Co. Ltd. (Guangzhou, China) were housed under specific pathogen free conditions at 25℃ with a 12-h light/dark cycle. The mice were randomly divided into two groups: sh-NC and sh-SIRT5 groups (six mice per group). The mice were subcutaneously injected with U-CH1 cells stablely transfected with sh-NC and sh-SIRT5 plasmids (1 × 10^6^ cells/100 µL). The volume of the tumors was measured every week, for four weeks. The tumor size was measured with a caliper and the volume was estimated using the formula: a × b^2^/ 2 (a, the longest diameter; b, the shortest diameter). The mice were euthanized using pentobarbital sodium (40 mg/kg, Sigma-Aldrich, St. Louis, MO,USA) after 28 days, and the tumors were harvested, weighed and subjected to immunohistochemistry (IHC).

### RT-qPCR

Total RNA from tissues and cells was extracted by TRIzol reagent (Yuanye Biotechnology Co. Ltd., Shanghai, China). Then, RNA was reverse transcribed into cDNA using the HiScript IV RT SuperMix for qPCR (Vazyme Biotechnology Co. Ltd., Nanjing, China), and the qPCR amplification experiment was performed using the ChamQ Universal SYBR qPCR Master Mix (Vazyme) with the reaction conditions: 95 °C for 30 s, 40 cycles of 95 °C for 10 s, 60 °C for 30 s, and a melt curve stage. Primers used in this study are synthesized by Tsingke Biotechnology Co. Ltd. (Beijing, China) and listed as follows: sirtuin (SIRT)5, forward, 5′-GCCATAGCCGAGTGTGAGAC-3′ and reverse, 5′-CAACTCCACAAGAGGTACATCG-3′; c-myc, forward, 5′-TCAAGAGGCGAACACACAAC-3′ and reverse, 5′-TAACTACCTTGGGGGCCTTT-3′; glyceraldehyde-3-phosphate dehydrogenase (GAPDH), 5′-TGTGGGCATCAATGGATTTGG-3′ and reverse, 5′-ACACCATGTATTCCGGGTCAAT-3′. The gene expression was calculated by the 2^−ΔΔCT^ method.

### Western blot

The Total Protein Extraction Kit (Solarbio Science & Technology Co., Ltd, Beijing, China) was used to extract the protein from tissues and cells according to the provided instructions. Then, the homogenate was homogenized in an ice bath, shaken (2 h), and centrifuged (12,000 rpm, 20 min) at 4℃. Then, the supernatant was taken and stored at -80℃ for use. Bicinchoninic acid (BCA) method (Solarbio) was used to detect the protein concentration. After that, 50 µg of protein was separated by 10% SDS-PAGE (Sigma) and transferred to the PVDF membrane (Solarbio). The membrane was blocked in 5% skim milk for 1 h in order to remove nonspecific adsorption, and incubated with the primary antibodies (Sigma) overnight at 4 °C. Then, the secondary antibody (Sigma) was incubated with the membrane for 1 h at room temperature after washing the membrane three times with Tris-buffered saline Tween (TBST, Solarbio). Finally, protein signal detection was performed using an enhanced chemiluminescence solution (Sigma). The specific proteins were visualized using the Odyssey™ Infrared Imaging System (Gene Company Limited, Hong Kong, China). GAPDH expression was used as an internal control to show equal loading of the protein samples. The used antibodies were listed as follows: SIRT5 (Abcam, Cambridge, MA, USA; ab259967; 1/1000), c-myc (Abcam; ab32072; 1/1000), succinyl lysine (PTM Biolabs, Hangzhou, China; PTM-401; 1/1000), glyceraldehyde 3-phosphate dehydrogenase (GAPDH) (Abcam; ab181602; 1/10,000), and goat anti-rabbit IgG (Abcam; ab205718; 1/5000).

### Cell counting kit-8 (CCK-8) assay

Cell viability was detected by the CCK-8 kit (Vazyme) according to the instructions. Three replicate wells were set up. Firstly, the cells were seeded into a 96-well plate (Corning) at the density of 1 × 10^3^ cells/well, then maintained in the incubator for 24 h. After that, 10 µL of CCK-8 solution was added to each well and incubated with cells for 2 h. Finally, a microplate reader (Thermo Fisher) was used to assess the absorbance at 450 nm.

### Colony formation assay

For colony formation detection, the cells were planted in 6-well plates and cultured at 37 °C for two weeks. Then, the cells were fixed with 4% paraformaldehyde (PFA, Sigma) and stained with 0.5% crystal violet (Solarbio). Finally, the stained cells were counted under a microscope (Olympus, Tokyo, Japan).

### Transwell migration and invasion assays

The Transwell migration and invasion assays were performed as previously described [18]. For the migration assays, the cells (5 × 10^4^/100 µL) were seeded onto the 24-well Transwells (Corning) uncoated with Matrigel. For the invasion assays, the cells (5 × 10^4^/100 µL) were seeded onto the 24-well Transwells pre-coated with 100 µL of Matrigel. The U-CH1 and U-CH2 cells were resuspended in serum-free medium and added to the upper chamber. The bottom chamber was prepared using 800 µL of RPMI-1640 supplemented with 10% FBS as a chemoattractant. After 48 h of incubation, the cells on the lower surface were washed with phosphate buffer solution (PBS, Vazyme), fixed with 4% PFA for 20 min, and stained in a dye solution containing 0.5% crystal violet for visualization. Finally, the cells were counted and photographed under a microscope. All experiments were performed in triplicate.

### Co-immunoprecipitation (Co-IP) assay

Co-IP assay was performed to detect the interaction relationship between SIRT5 and c-myc in HEK-293T cells. After washing by pro-cooled PBS twice, the cells were lysed in Radio Immunoprecipitation Assay Lysis buffer (RIPA) buffer (Vazyme) containing protease inhibitors on ice for 30 min. Then, the supernatant was collected after centrifugating (12,000 *g*, 10 min), and 10 µL of it was taken as the input group. After that, SIRT5, c-myc, or lgG antibody (2 µg) was added into the remaining supernatant and incubated with protein G Plus-Agarose Immunoprecipitation reagent (Abcam) at 4 °C overnight. IgG was used as a negative control. Next, the beads were washed with lysis buffer four times. Immunoprecipitates were eluted by boiling with 1% (wt: vol) SDS sample buffer (Vazyme) and boiled at 100 °C for 5 min. The protein-protein complexes were subsequently subjected to Western blot. The labeled protein membranes were observed and quantified using the Tanon 5200 system (Shanghai, China). The used antibodies were listed as follows: SIRT5 (Abcam; ab259967; 1/30), GAPDH (Abcam; ab181602; 1/60), c-myc (Thermo Fisher; PA5-85185; 1/500), and goat anti-rabbit IgG (Thermo Fisher; 31,460; 1/1000).

### Ubiquitination assay

IP assay combined with Western blot were used to access the succinylation level of c-myc (PTM Biotechnology Co., Ltd, Hangzhou, China) in HEK-293T cells. Briefly, the HEK-293T cell lysates were obtained and immunoprecipitated with c-myc antibody c-myc (Thermo Fisher; PA5-85185; 1/500) and protein A/G agarose, followed by Western blot against ubiquitin.

### IHC

Paraffin-embedded tumor tissues were cut into sections (about 5 μm), dewaxed, rehydrated, and treated with 3% H_2_O_2_, respectively. Then, the sections were treated with 10 mM citrate buffer (Yuanye) and boiled in microwave oven for 3 min to expose the site of the antigen. After cooling to room temperature, the citrate buffer was discarded and the sections were washed by PBS twice. Next, normal goat serum was used to block the sections at 37℃ for 30 min. After that, the sections were incubated with primary antibodies against SIRT5 and c-myc overnight at 4℃, and were then treated with goat anti-rabbit IgG at 37℃ for 30 min. After adding DAB solution (Vazyme), the sections were counterstained using hematoxylin (Vazyme) and blued in 1% ammonia water. Finally, the sections were dehydrated, sealed, and observed under a light microscope (Leica Microsystems Trading LTD., Shanghai, China). The used antibodies were listed as follows: SIRT5 (Abcam; ab259967; 1/100), c-myc (Abcam; ab185656; 1/500), GAPDH (Abcam; ab181602; 1/2000), and goat anti-rabbit IgG (Abcam; ab205718; 1/5000).

### Protein stability assessment

The cells were treated with cycloheximide (CHX, 100 µg/mL, Yuanye), and the protein level of c-myc at different time points (0, 8, 16, and 24 h) was analyzed by Western blot.

### Statistical analysis

The SPSS 21.0 software was used to analyze data. Data are expressed as mean ± standard deviation (SD). Student’s t-test was used for comparison between the two groups. One-way analysis of variance (ANOVA) was used for comparison among groups. Statistical analyses were performed using GraphPad Prism software (v8.0.1, GraphPad Software Inc., San Diego, CA, USA). *p* < 0.05 indicates that the difference is statistically significant.

## Results

### High expression of SIRT5 in chordoma tissues and cells

**Fig. 1:**
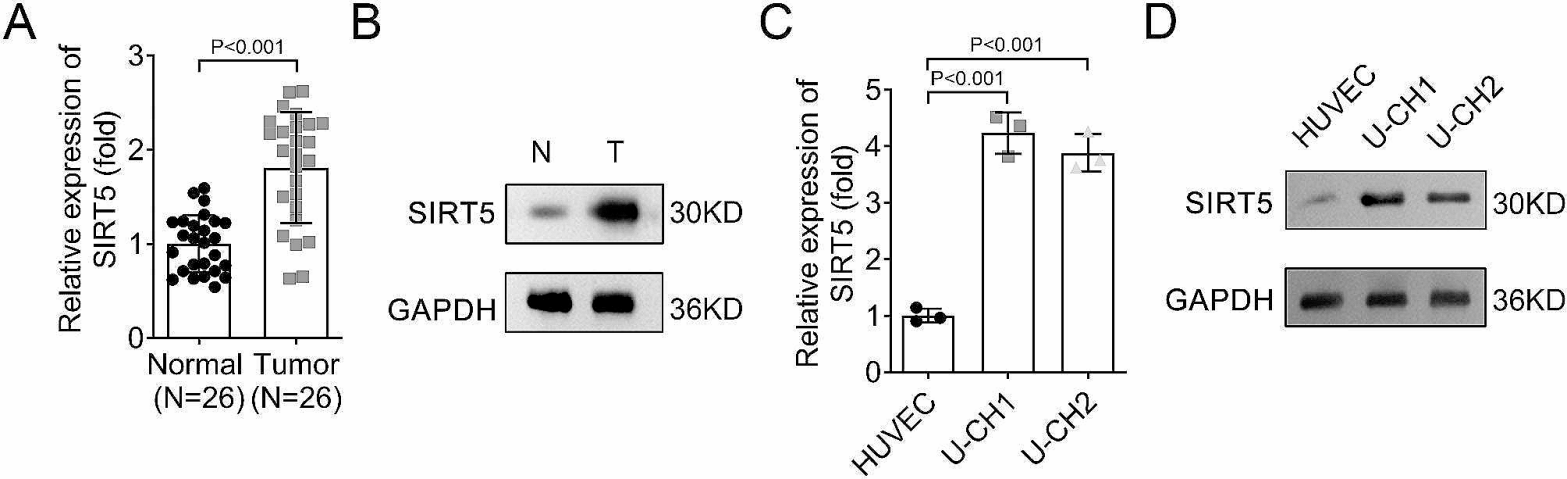
High expression of SIRT5 in chordoma tissues and cells. **A**, RT-qPCR and **B**, Western blot were performed to analyze the mRNA and protein levels of SIRT5 in normal and tumor tissues; The expression of SIRT5 in HUVEC, U-CH1, and U-CH2 cells was analyzed by **C**, RT-qPCR and **D**, Western blot. **SIRT**, sirtuin; **HUVEC**, human umbilical vein endothelial cells; **RT-qPCR**, reverse transcription-quantitative polymerase chain reaction

There are few studies on the posttranslational modification of chordoma. Previous articles have found that the progression of chordoma was regulated by ubiquitination modification [19, 20]. Succinylation is a research hotspot in cancer-related diseases in recent years. However, the effects of succinylation on chordoma have not been researched. In this study, we detected the expression of SIRT5 in normal and tumor tissues. The RT-qPCR and Western blot results indicated that the mRNA and protein levels of SIRT5 were increased in tumor tissues in comparison with the normal ones (Fig. 1A and B). Moreover, as indicated in Table 1, 26 patients were divided into high expression (*n* = 13) and low expression (*n* = 13) according to the expression of SIRT5, further analysis demonstrated that the SIRT5 level was independent of age, sex, tumor location, tumor size, and whether the tumor was totally resected, but correlated with whether the tumor was the primary tumor. In addition, the mRNA and protein levels of SIRT5 in U-CH1 and U-CH2 cells were higher than that in HUVEC cells (Fig. 1C and D).

### Knockdown of SIRT5 inhibited cell proliferation, migration, and invasion of U-CH1 and U-CH2 cells

**Fig. 2:**
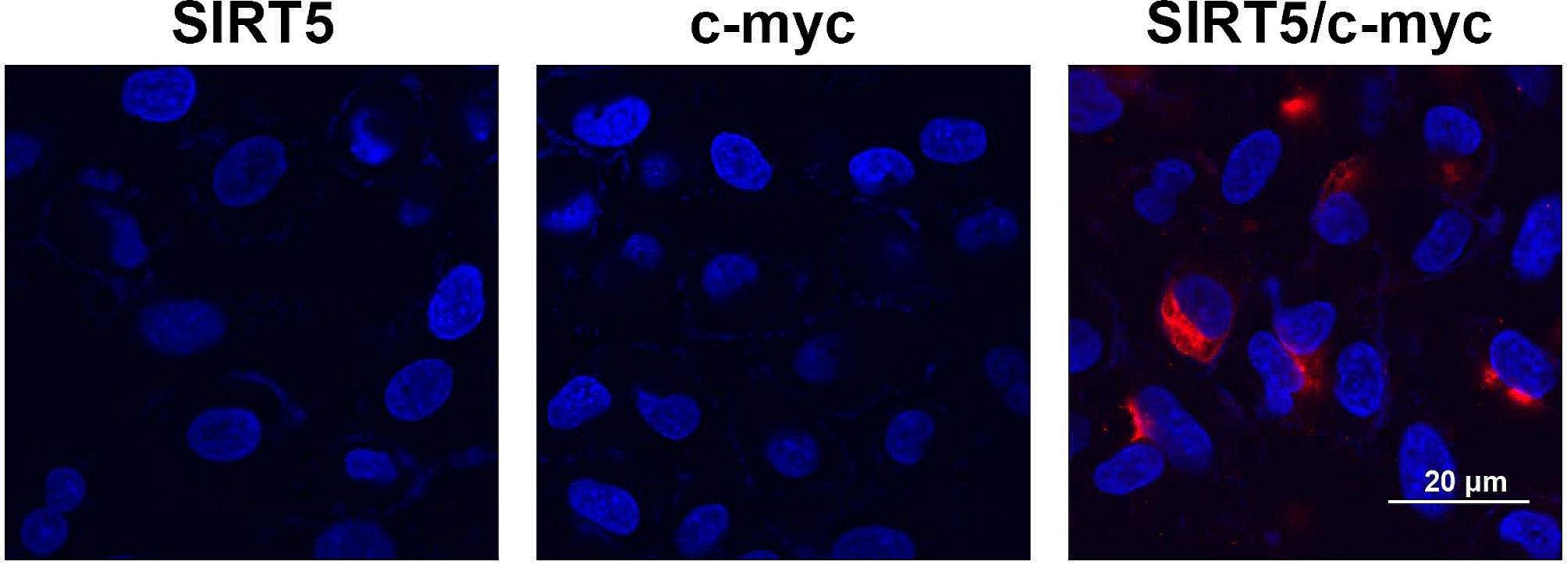
Knockdown of SIRT5 inhibited cell proliferation, migration, and invasion of U-CH1 and U-CH2 cells. The expression of SIRT5 in sh-NC and sh-SIRT5 groups in U-CH1 and U-CH2 cells was detected by **A**, RT-qPCR and **B**, Western blot; **C**, CCK-8 assay was performed to assess the cell viability of U-CH1 and U-CH2 cells; **D**, Cell colonies were evaluated by colony formation analysis (scale bars = 0.5 cm); **E**, The colony number in sh-NC and sh-SIRT5 groups in U-CH1 and U-CH2 cells; **F**, Transwell assay was performed to detect cell migration (magnification, ×200); **G**, The migration cell number in sh-NC and sh-SIRT5 groups in U-CH1 and U-CH2 cells; **H**, Cell invasion was detected by Transwell assay (magnification, ×200); **I**, The invasion cell number in sh-NC and sh-SIRT5 groups in U-CH1 and U-CH2 cells. **SIRT**, sirtuin; **RT-qPCR**, reverse transcription-polymerase chain reaction; **CCK-8**, cell counting kit-8; **sh-RNA**, short hairpin RNA.

Then, we conducted the in vitro study to explore the role of SIRT5 in chordoma. After transfecting sh-SIRT5 into U-CH1 and U-CH2 cells, the mRNA and protein levels of SIRT5 were downregulated (Fig. 2A and B). The cell viability of U-CH1 and U-CH2 cells was suppressed after SIRT5 inhibition (Fig. 2C). The colony formation and Transwell assays results showed that the colony number, migration cell number, and invasion cell number were downregulated after SIRT5 inhibition compared with the sh-NC group (Fig. 2D-I). These results suggested that silencing SIRT5 suppressed cell proliferation, migration, and invasion in chordoma.

### SIRT5 interacted with c-myc to inhibit the succinylation of c-myc at K369 site

**Fig. 3:**
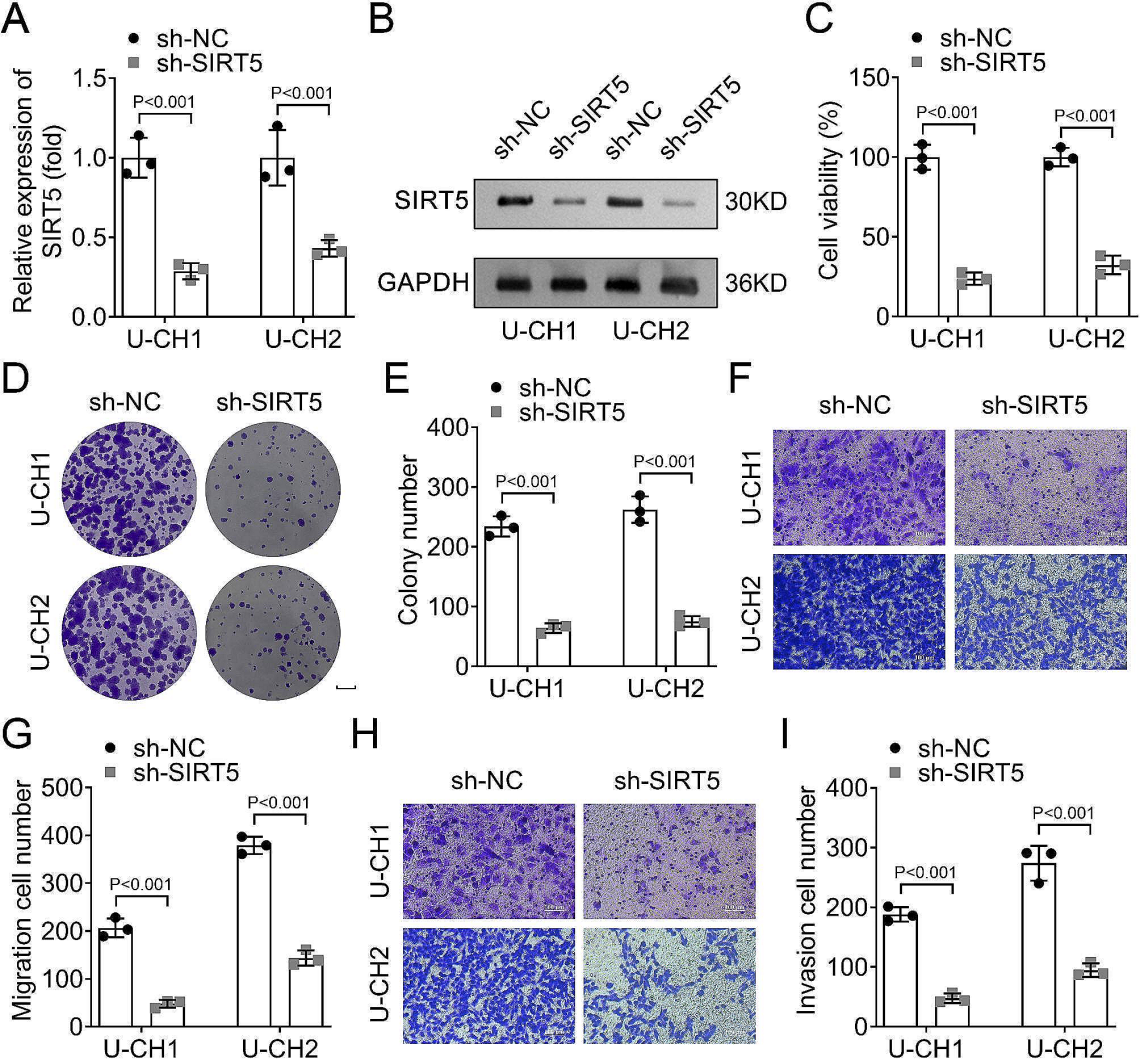
SIRT5 interacted with c-myc to inhibit the succinylation of c-myc at K369 site. **A**, The succinylation level in sh-NC and sh-SIRT5 groups in HEK-293T cells was assessed by Western blot; **B**, STRING database was used to screen SIRT5-related proteins; **C**, Co-IP assay was performed to detect the interaction between SIRT5 and c-myc; **D**, The protein levels of c-myc and c-myc-succ in HEK-293T cells were detected by IP and Western blot after SIRT5 knockdown; **E**, The succinylation sites for c-myc were predicted using GPSuc database; **F**, IP and Western blot assays were used to analyze the succinylation sites of c-myc; **G**, The protein expression of c-myc was assessed by Western blot at the different time points (0, 8, 16, and 24 h) in HEK-293T cells; **H**, Quantification of the existing c-myc protein level at different time points (0, 8, 16, and 24 h) in HEK-293T cells. **SIRT**, sirtuin; **STRING**, Search Tool for Recurring Instances of Neighbouring Genes; **Co-IP**, co-immunoprecipitation; **succ**, succinylation

After silencing SIRT5 in HEK-293T cells, the protein level of succinylation was increased (Fig. 3A). STRING database was used to screen SIRT5-associated proteins (Fig. 3B). A previous study has indicated that SIRT5-dependent genes include the c-myc proto-oncogene [21]. Thus, we assessed the interaction between SIRT5 and c-myc. Co-IP assay results showed that SIRT5 was interacted with c-myc in HEK-293T cells (Fig. 3C). In addition, proximity ligation assay was conducted to further demonstrate the interaction between SIRT5 and c-myc in HEK-293T cells (Supplementary Fig. 1). Besides, inhibiting SIRT5 obviously reduced the protein level of c-myc and increased that of c-myc-succ (Fig. 3D). In order to further identify the succinylation sites of c-myc, we used the GPSuc database for prediction, and the results showed three possible c-myc succinylation sites, K369, K385, and K411 (Fig. 3E). Then, arginine mutations were introduced at K369, K385, and K411 sites of c-myc. IP and Western blot results showed that arginine mutation of K369 site showed upregulated c-myc protein level and downregulated c-myc-succ protein level (Fig. 3F) rather than K385 and K411 sites, suggesting that c-myc was succinylated at K369 site in HEK-293T cells. Protein stability assay results revealed that inhibition of SIRT5 downregulated the protein stability of c-myc in HEK-293T cells (Fig. 3G and H).

***Overexpressing c-myc reversed the decreased cell viability, colony number, migration and invasion cell numbers caused by silencing SIRT5 in U-CH1 and U-CH2 cells***.

**Fig. 4:**
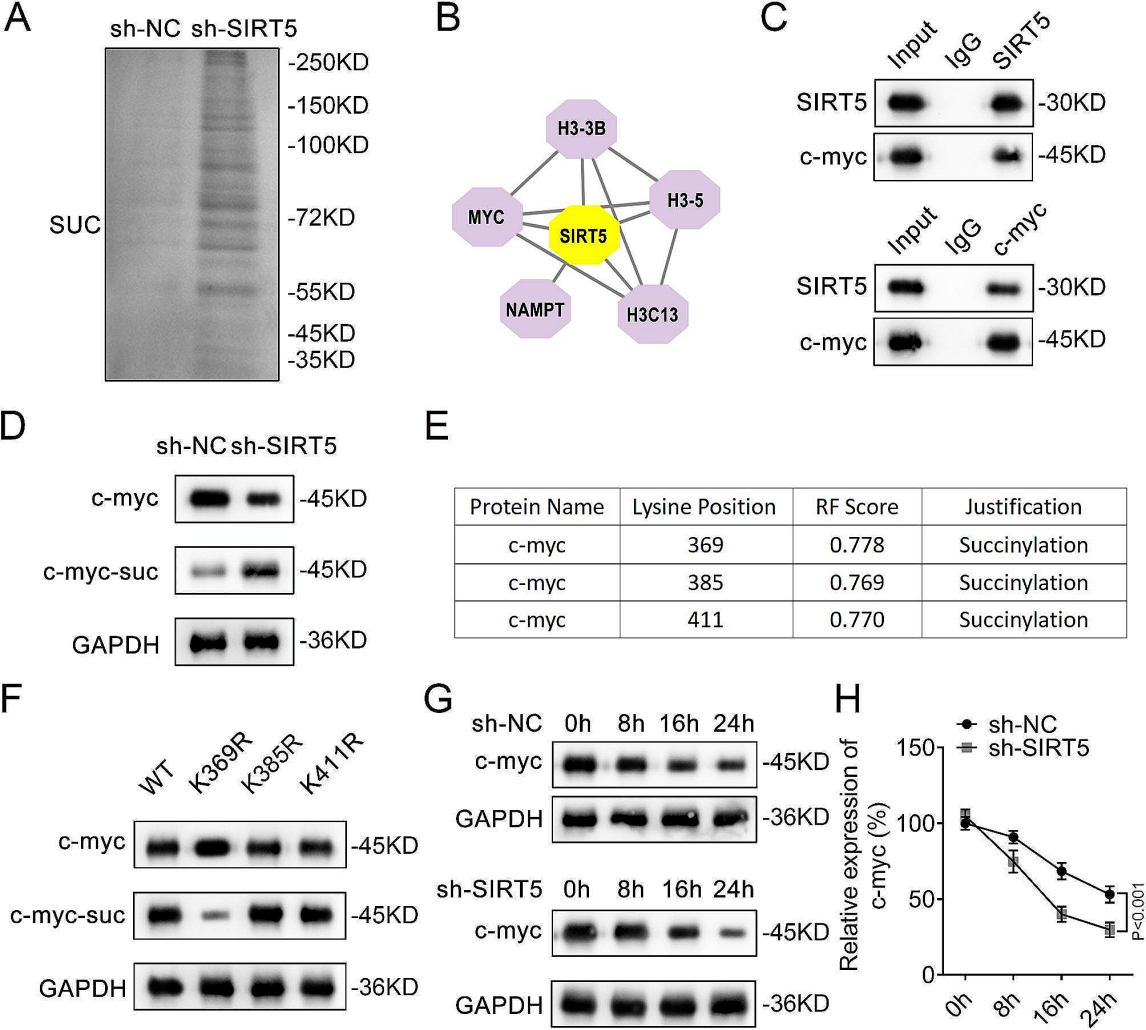
Overexpressing c-myc reversed the decreased cell viability, colony number, migration and invasion cell numbers caused by silencing SIRT5 in U-CH1 and U-CH2 cells. The expression of c-myc after c-myc overexpression in U-CH1 and U-CH2 cells was detected by **A**, RT-qPCR and **B**, Western blot; **C**, CCK-8 assay was performed to assess the viability of U-CH1 and U-CH2 cells in each group; **D**, Cell colonies were evaluated by colony formation analysis(scale bars = 0.5 cm); **E**, The colony number in each group in U-CH1 and U-CH2 cells; **F**, Transwell assay was performed to detect cell migration (magnification, ×200); **G**, The migration cell number in each group in U-CH1 and U-CH2 cells; **H**, Cell invasion was detected by Transwell assay (magnification, ×200); **I**, The invasion cell number in each group in U-CH1 and U-CH2 cells. **SIRT**, sirtuin; **RT-qPCR**, reverse transcription-polymerase chain reaction; **CCK-8**, cell counting kit-8; **sh-RNA**, short hairpin RNA.

After transfecting c-myc overexpression vector into U-CH1 and U-CH2 cells, the mRNA and protein levels of c-myc were increased (Fig. 4A and B). In rescue experiments, the results showed that silencing SIRT5 decreased the cell viability, colony number, migration, and invasion cell numbers compared with the sh-NC group in U-CH1 and U-CH2 cells. Besides, overexpression of c-myc increased the cell viability, colony number, migration and invasion cell numbers in U-CH1 and U-CH2 cells in comparison with the sh-SIRT5 + Vector group (Fig. 4C-I).

### Silencing SIRT5 inhibited tumor growth of mice

**Fig. 5:**
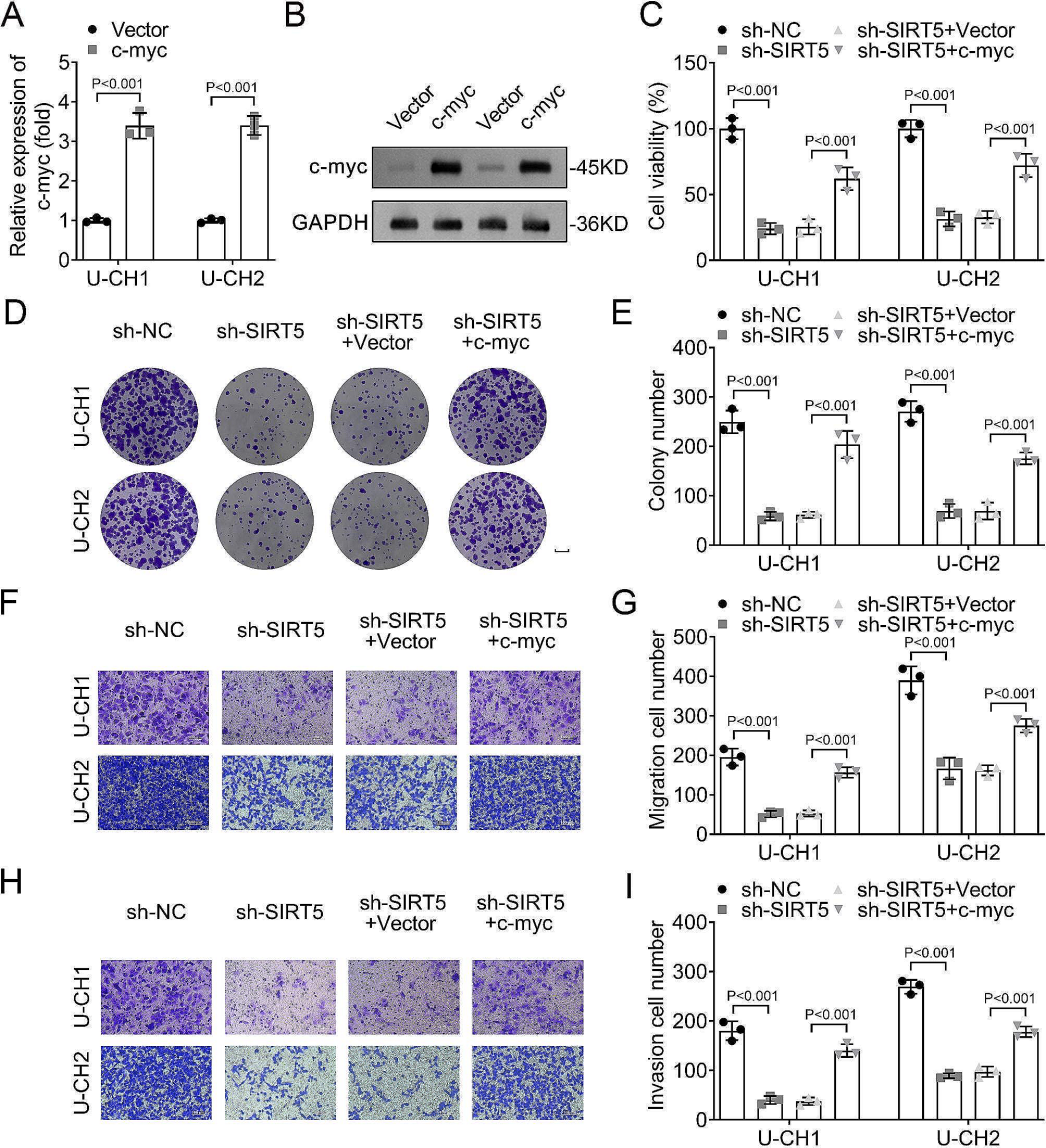
Silencing SIRT5 inhibited tumor growth of mice. **A**, The tumor size, **B**, weight, and **C**, volume in sh-SIRT5 and sh-NC groups; **D**, IHC assay was performed to assess the SIRT5 and c-myc protein levels in sh-SIRT5 and sh-NC groups (scale bar = 100 μm). **SIRT**, sirtuin; **sh-RNA**, short hairpin RNA; **IHC**, immunohistochemistry

The tumor size, weight and volume were suppressed in the sh-SIRT5 group compared with that in the sh-NC group (Fig. 5A-C). IHC assay showed that the protein levels of SIRT5 and c-myc were lower in the sh-SIRT5 group than that in the sh-NC group (Fig. 5D and E).

## Discussion

The notochord is thought to be an axial skeleton of primitive structure that promotes the development of surrounding tissues in the early embryo [22]. Chordoma is a rare mesenchymal malignancy with a high recurrence rate, and its tumorigenic mechanism remains unclear. Previous studies have found that genetic alterations, epigenetic regulators, and chromatin spatial organization play crucial roles in the development and progression of chordoma [23]. However, the role of post-translational modification of proteins in chordoma is rarely studied. Succinylation modification has been shown to play key roles in other bone-related diseases, including compression-induced intervertebral disc [15] and early osteoarthritis [24]. However, no study has investigated the effect of succinylation on the development of chordoma. In this study, we found that SIRT5 was increased in chordoma tissues and cells. SIRT5 is the only known desuccinylase [25]. Besides, we found that silencing SIRT5 inhibited proliferation, migration, and invasion of chordoma cells, indicating that SIRT5 promoted malignant advancement of chordoma. Similarly, a previous study demonstrates that SIRT5 deletion promotes obesity-associated osteoarthritis development [24]. In addition, the role of other members of the SIRT family in bone-related diseases has also been described [26, 27]. Interestingly, we found that low level of SIRT5 was also associated with tumors that are not primary chordoma (6 out of 13), suggesting that other primary tumors may have a low level of SIRT5 or that other primary tumors may have a high level of SIRT5 while the SIRT5 level was decreased when progressing and metastasizing.

Additionally, we used HEK-293T cells to further explore the mechanism by which SIRT5 affected the progression of chordoma. The results found for the first time that SIRT5 inhibited the succinylation of c-myc. Similar with our result, a previous study has showed that c-myc is a SIRT5-dependent gene in melanoma [21]. C-myc is located on human chromosome 8, participates in various physiological process, such as cell cycle progression, proliferation and apoptosis [28]. Besides, c-myc is a vital oncogenic transcription factor and closely associated with the progression of different tumor-related diseases [29, 30]. Interestingly, our results also found that c-myc was succinylated at K369 site, which has never been reported in previous studies. This could provide a reference for further study to explore the specific mechanism of c-myc in chordoma or other tumor-related diseases. Moreover, further rescue experiments found that overexpression of c-myc reversed the decreases of proliferation, migration, and invasion by silencing SIRT5 in chordoma cells, further supporting the above results. In in vivo study, we found that SIRT5 inhibition suppressed the growth of mice, which was consistent with clinical and in vitro studies.

In summary, this finding indicated that SIRT5-mediated desuccinylation of c-myc promoted malignant advancement of chordoma, which might provide new ideas for the clinical treatment of chordoma. However, there are still some limitations in this study, such as a small clinical sample size, which will be further explored in future studies.

### Electronic supplementary material

Below is the link to the electronic supplementary material.

Supplementary Material 1

Supplementary Material 2

Supplementary Material 3

Supplementary Material 4

Supplementary Material 5

Supplementary Material 6

## Data Availability

The datasets used and/or analyzed during the current study are available from the corresponding author on reasonable request.
