# Supplementary figures and images for: SIRT5 promote malignant advancement of chordoma by regulating the desuccinylation of c-myc
Source: BMC Cancer. 2024 Mar 26;24:386. doi: 10.1186/s12885-024-12140-w (PMC10967166; doi:10.1186/s12885-024-12140-w)

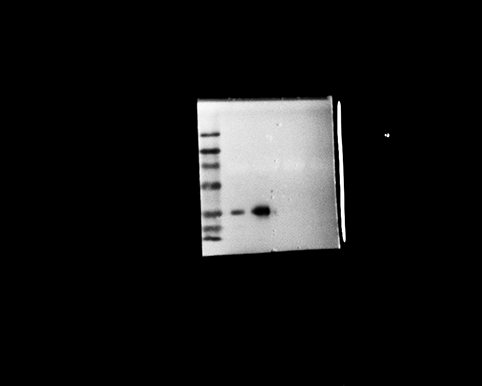

Supplement: Supplementary file 2 — Supplementary Material 2 [file 12885_2024_12140_MOESM2_ESM.zip › Fig.1/1B/1-SIRT5.tif]

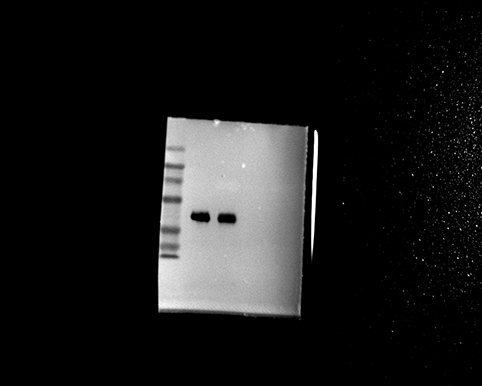

Supplement: Supplementary file 2 — Supplementary Material 2 [file 12885_2024_12140_MOESM2_ESM.zip › Fig.1/1B/2-GAPDH.tif]

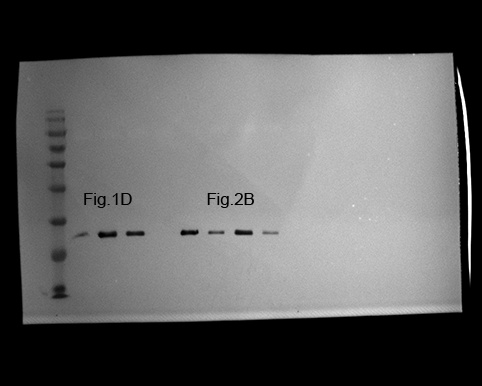

Supplement: Supplementary file 2 — Supplementary Material 2 [file 12885_2024_12140_MOESM2_ESM.zip › Fig.1/1D/1-SIRT5.tif]

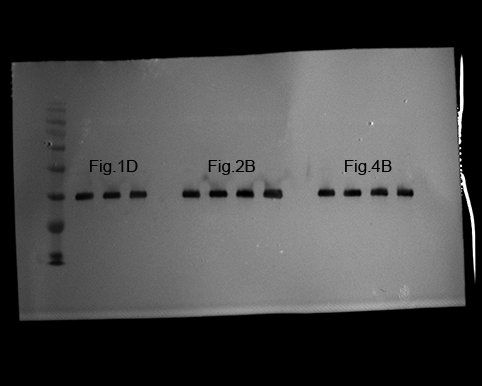

Supplement: Supplementary file 2 — Supplementary Material 2 [file 12885_2024_12140_MOESM2_ESM.zip › Fig.1/1D/2-GAPDH.tif]

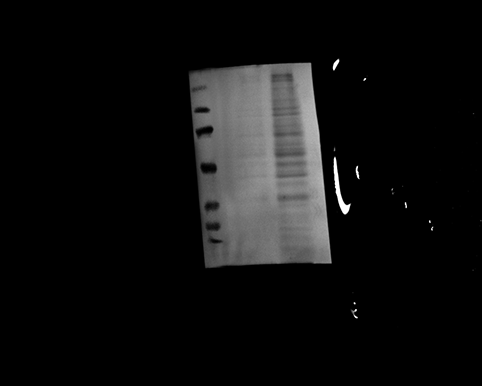

Supplement: Supplementary file 3 — Supplementary Material 3 [file 12885_2024_12140_MOESM3_ESM.zip › Fig.3/3A/suc.tif]

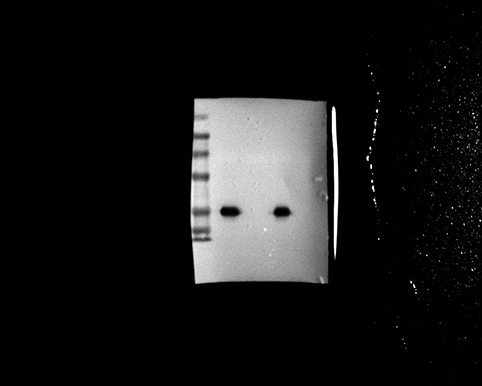

Supplement: Supplementary file 3 — Supplementary Material 3 [file 12885_2024_12140_MOESM3_ESM.zip › Fig.3/3C/1-SIRT5.tif]

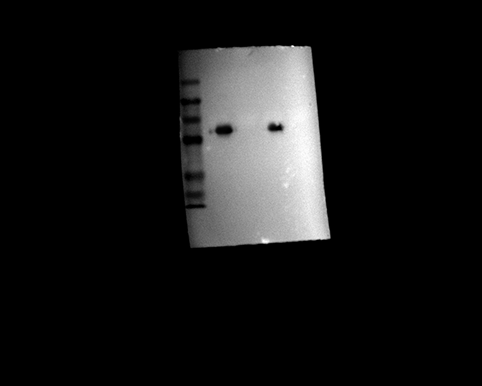

Supplement: Supplementary file 3 — Supplementary Material 3 [file 12885_2024_12140_MOESM3_ESM.zip › Fig.3/3C/2-c-myc.tif]

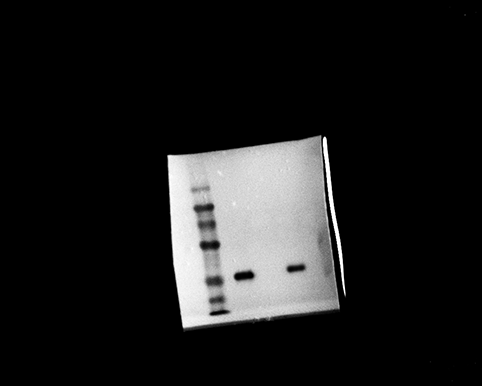

Supplement: Supplementary file 3 — Supplementary Material 3 [file 12885_2024_12140_MOESM3_ESM.zip › Fig.3/3C/3-SIRT5.tif]

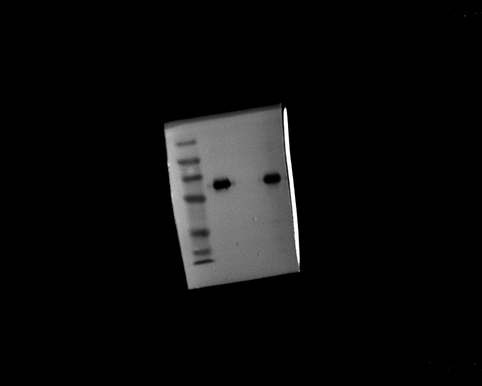

Supplement: Supplementary file 3 — Supplementary Material 3 [file 12885_2024_12140_MOESM3_ESM.zip › Fig.3/3C/4-c-myc.tif]

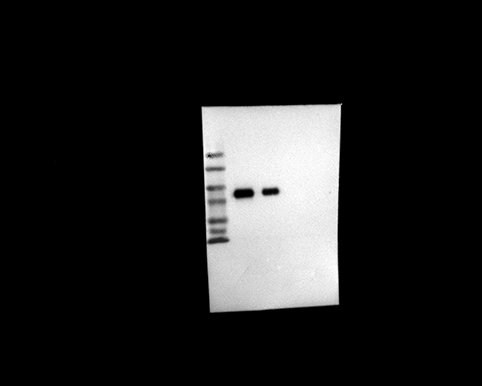

Supplement: Supplementary file 3 — Supplementary Material 3 [file 12885_2024_12140_MOESM3_ESM.zip › Fig.3/3D/1-c-myc.tif]

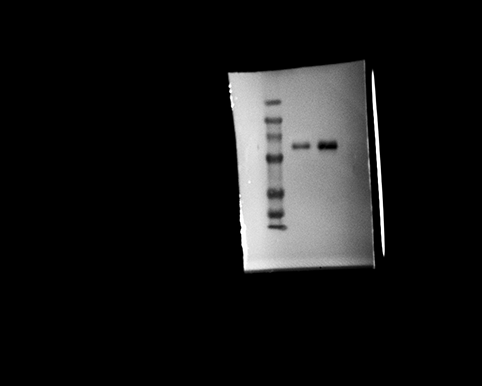

Supplement: Supplementary file 3 — Supplementary Material 3 [file 12885_2024_12140_MOESM3_ESM.zip › Fig.3/3D/2-c-myc-suc.tif]

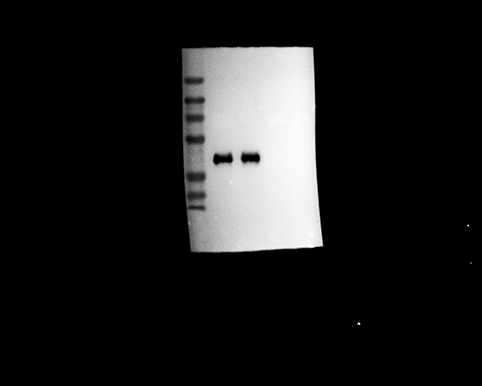

Supplement: Supplementary file 3 — Supplementary Material 3 [file 12885_2024_12140_MOESM3_ESM.zip › Fig.3/3D/3-GAPDH.tif]

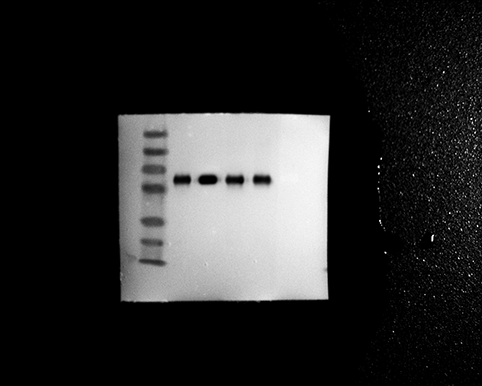

Supplement: Supplementary file 3 — Supplementary Material 3 [file 12885_2024_12140_MOESM3_ESM.zip › Fig.3/3F/1-c-myc.tif]

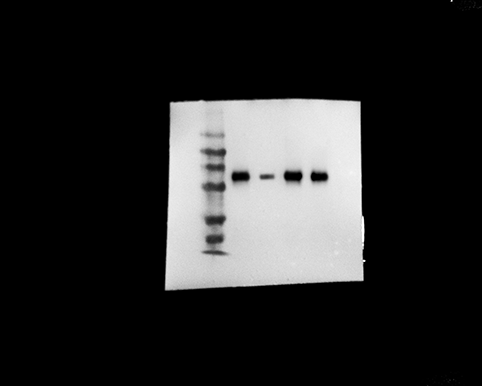

Supplement: Supplementary file 3 — Supplementary Material 3 [file 12885_2024_12140_MOESM3_ESM.zip › Fig.3/3F/2-c-myc-suc.tif]

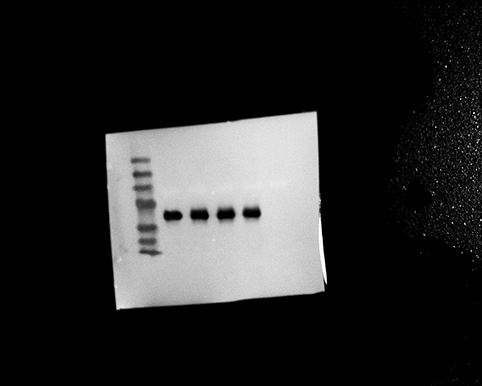

Supplement: Supplementary file 3 — Supplementary Material 3 [file 12885_2024_12140_MOESM3_ESM.zip › Fig.3/3F/3-GAPDH.tif]

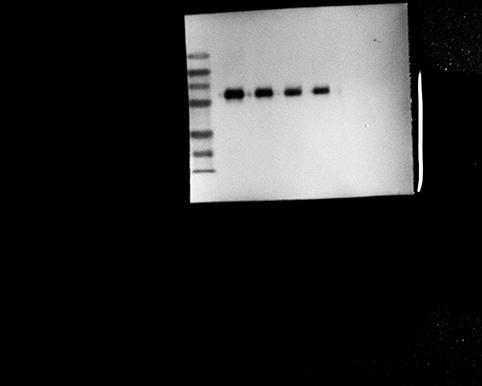

Supplement: Supplementary file 3 — Supplementary Material 3 [file 12885_2024_12140_MOESM3_ESM.zip › Fig.3/3G/1-c-myc.tif]

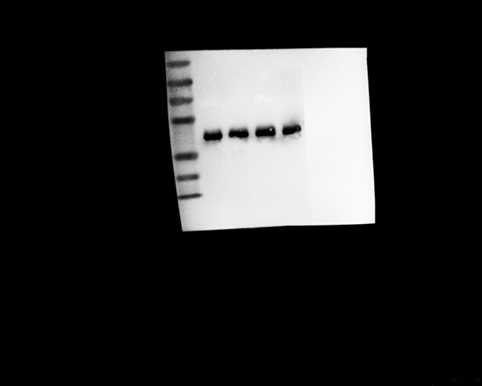

Supplement: Supplementary file 3 — Supplementary Material 3 [file 12885_2024_12140_MOESM3_ESM.zip › Fig.3/3G/2-GAPDH.tif]

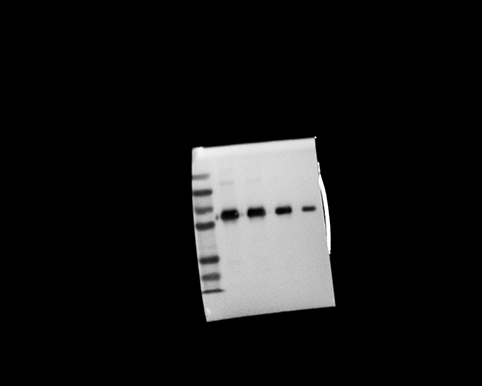

Supplement: Supplementary file 3 — Supplementary Material 3 [file 12885_2024_12140_MOESM3_ESM.zip › Fig.3/3G/3-c-myc.tif]

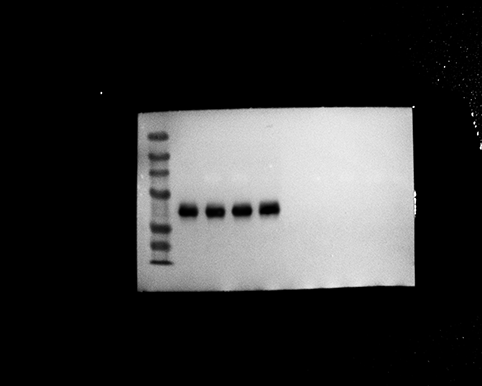

Supplement: Supplementary file 3 — Supplementary Material 3 [file 12885_2024_12140_MOESM3_ESM.zip › Fig.3/3G/4-GAPDH.tif]

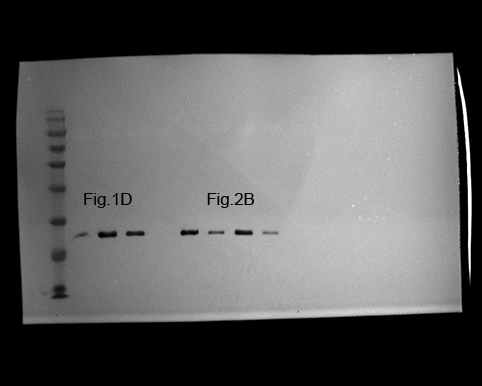

Supplement: Supplementary file 4 — Supplementary Material 4 [file 12885_2024_12140_MOESM4_ESM.zip › Fig.2/2B/1-SIRT5.tif]

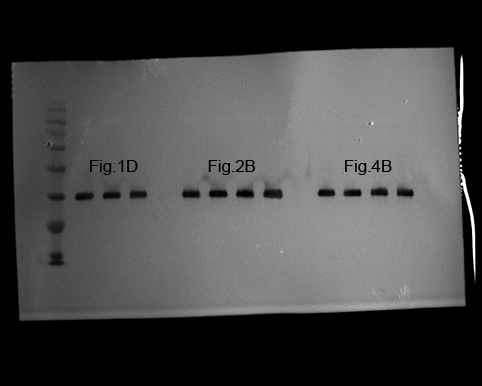

Supplement: Supplementary file 4 — Supplementary Material 4 [file 12885_2024_12140_MOESM4_ESM.zip › Fig.2/2B/2-GAPDH.tif]

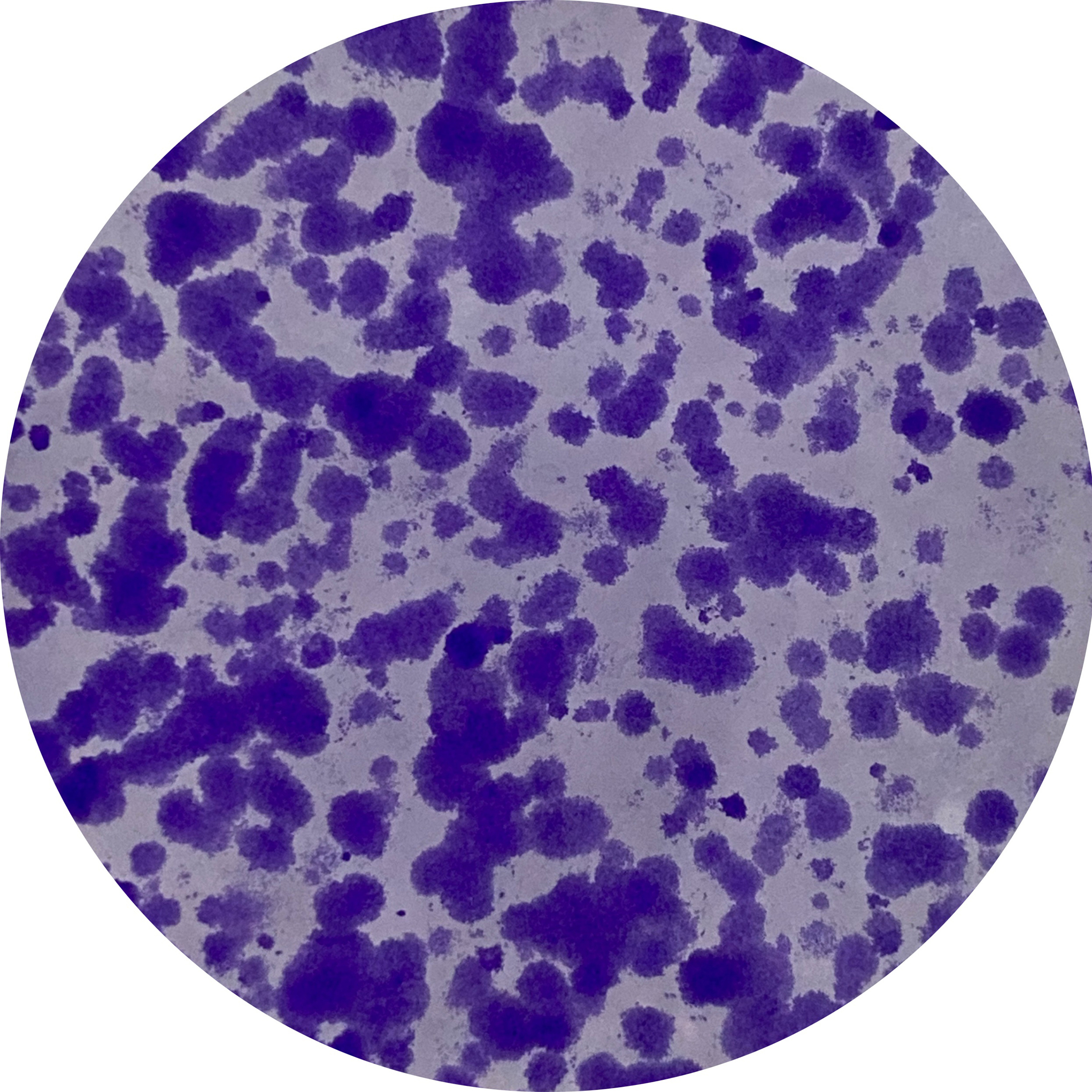

Supplement: Supplementary file 4 — Supplementary Material 4 [file 12885_2024_12140_MOESM4_ESM.zip › Fig.2/2D/U-CH1/2-1-1.jpg]

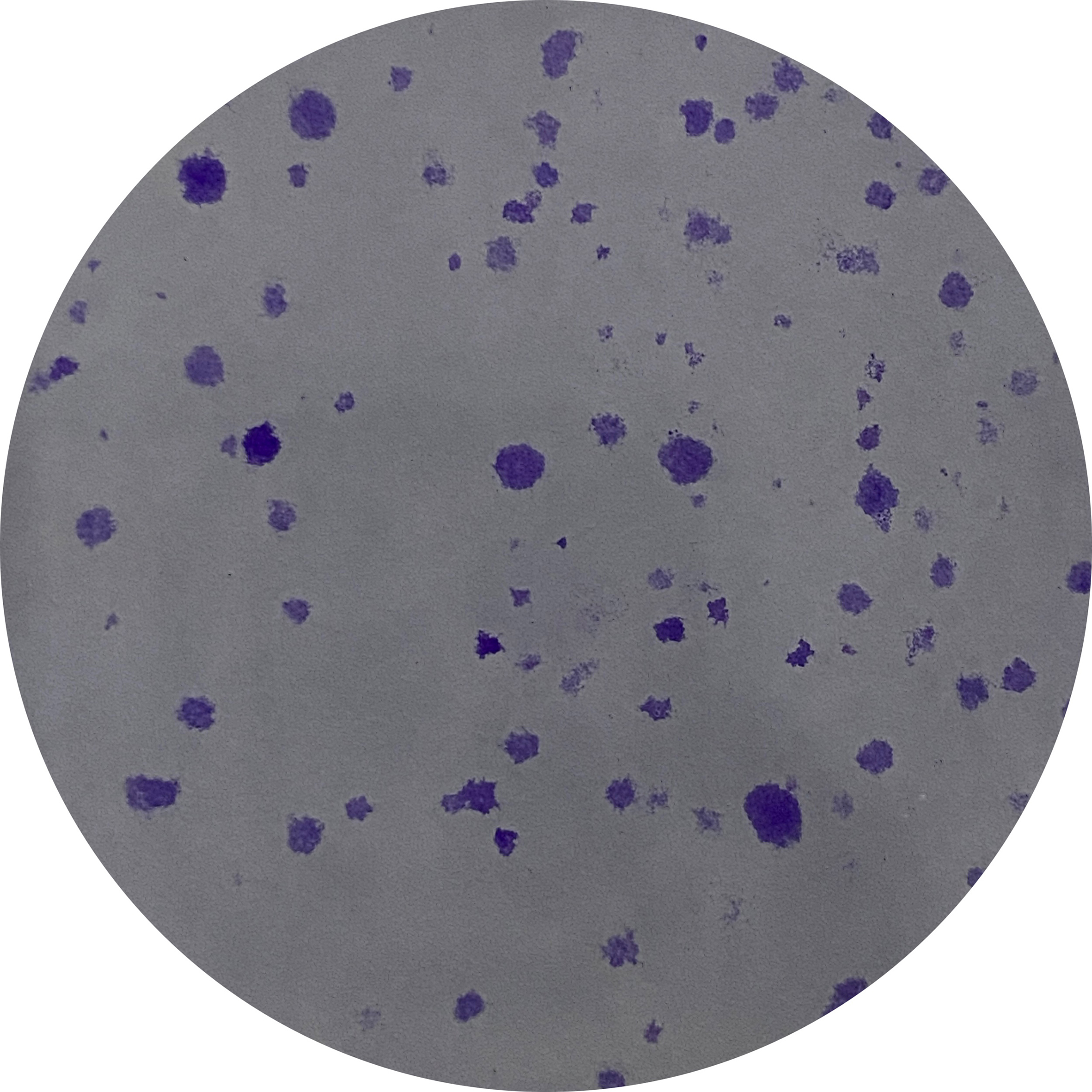

Supplement: Supplementary file 4 — Supplementary Material 4 [file 12885_2024_12140_MOESM4_ESM.zip › Fig.2/2D/U-CH1/2-1-2.jpg]

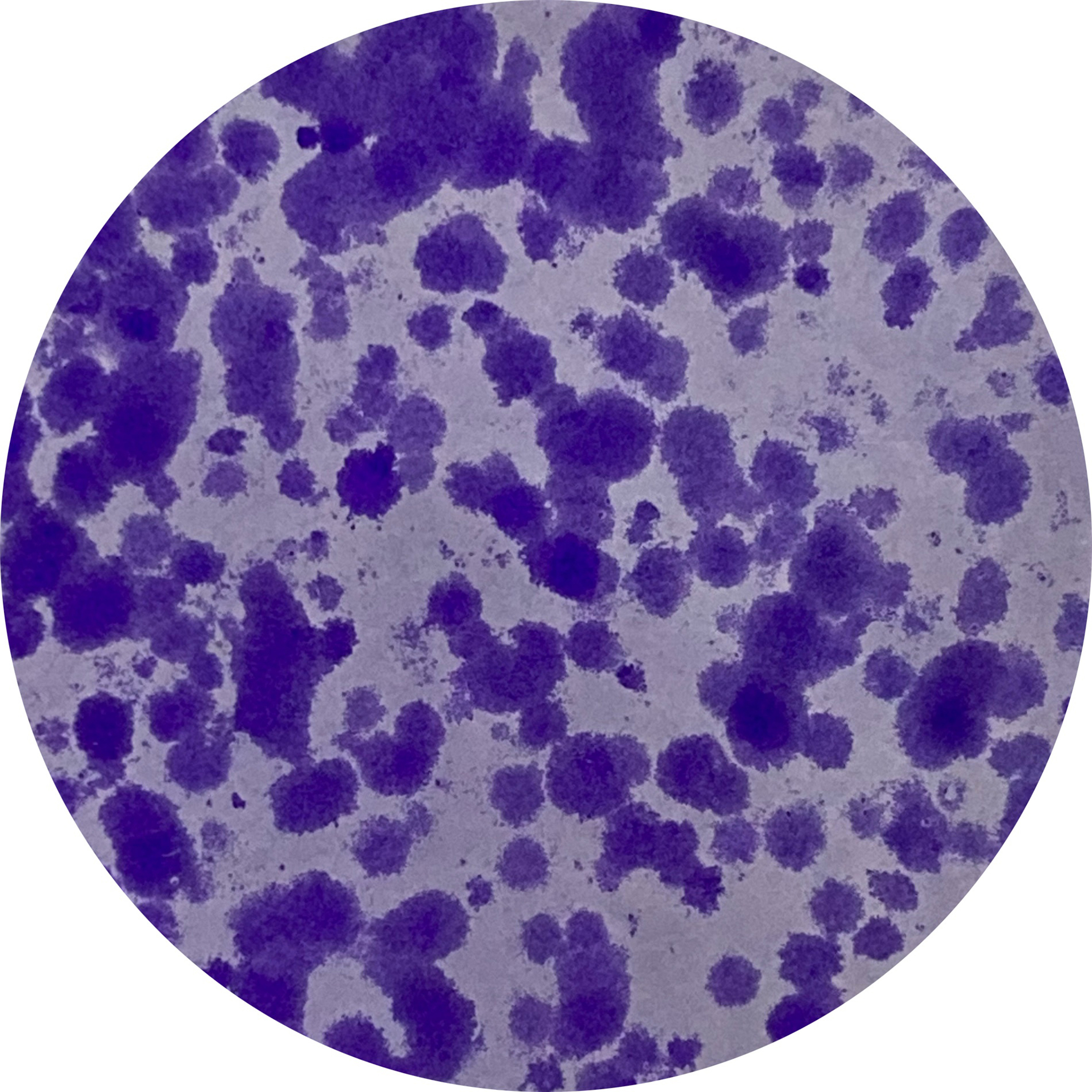

Supplement: Supplementary file 4 — Supplementary Material 4 [file 12885_2024_12140_MOESM4_ESM.zip › Fig.2/2D/U-CH2/2-2-1.jpg]

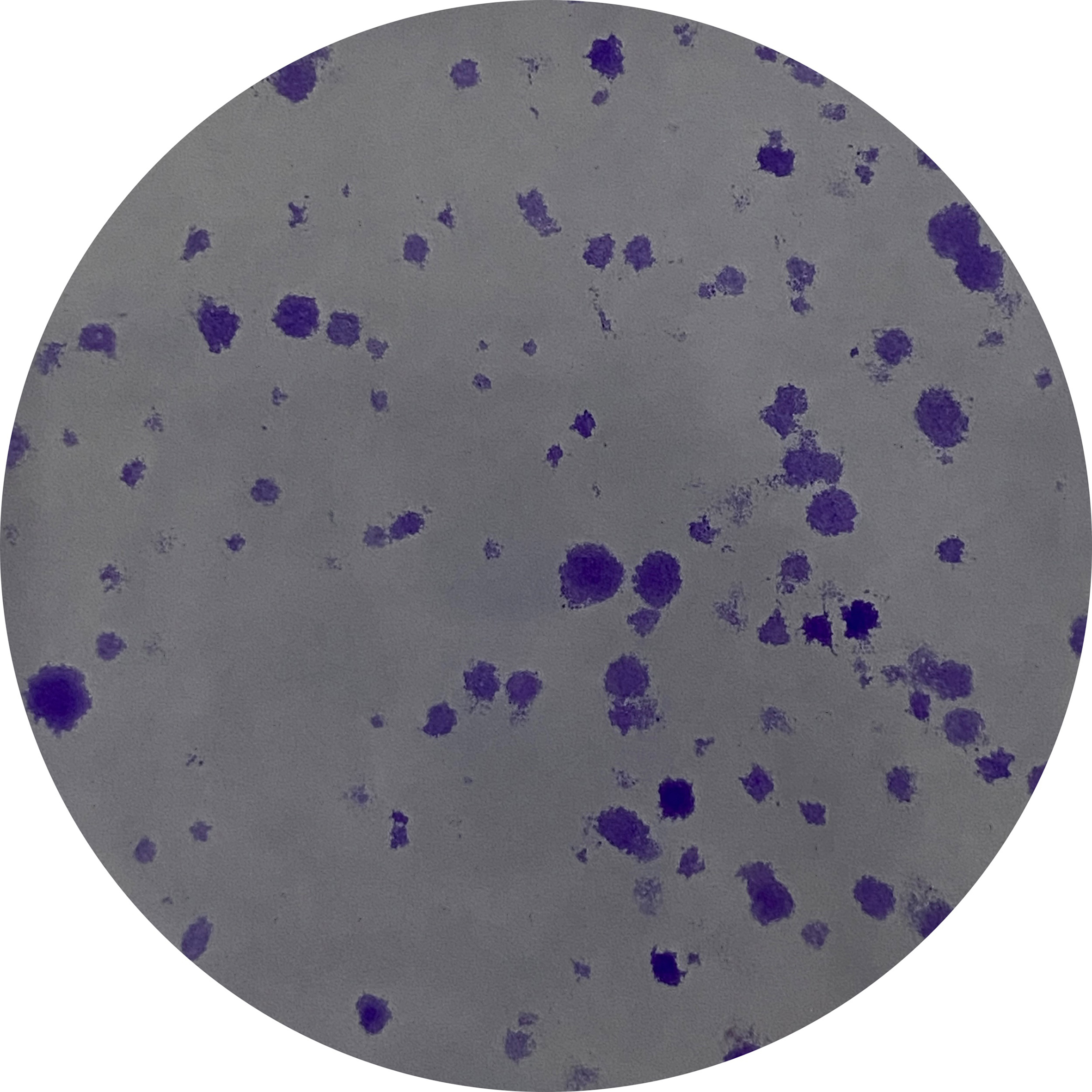

Supplement: Supplementary file 4 — Supplementary Material 4 [file 12885_2024_12140_MOESM4_ESM.zip › Fig.2/2D/U-CH2/2-2-2.jpg]

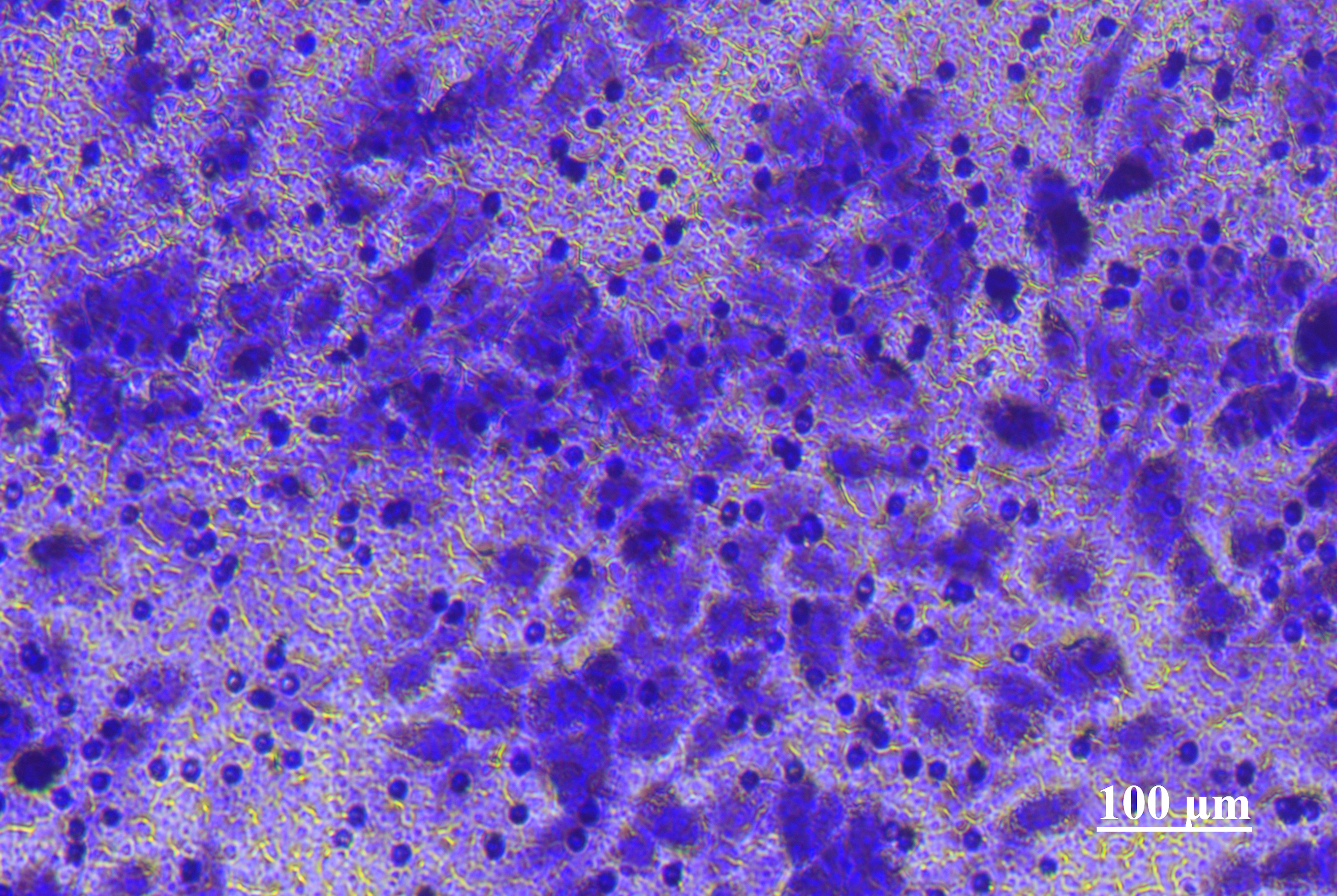

Supplement: Supplementary file 4 — Supplementary Material 4 [file 12885_2024_12140_MOESM4_ESM.zip › Fig.2/2F/U-CH1/MI-2-1-1.jpg]

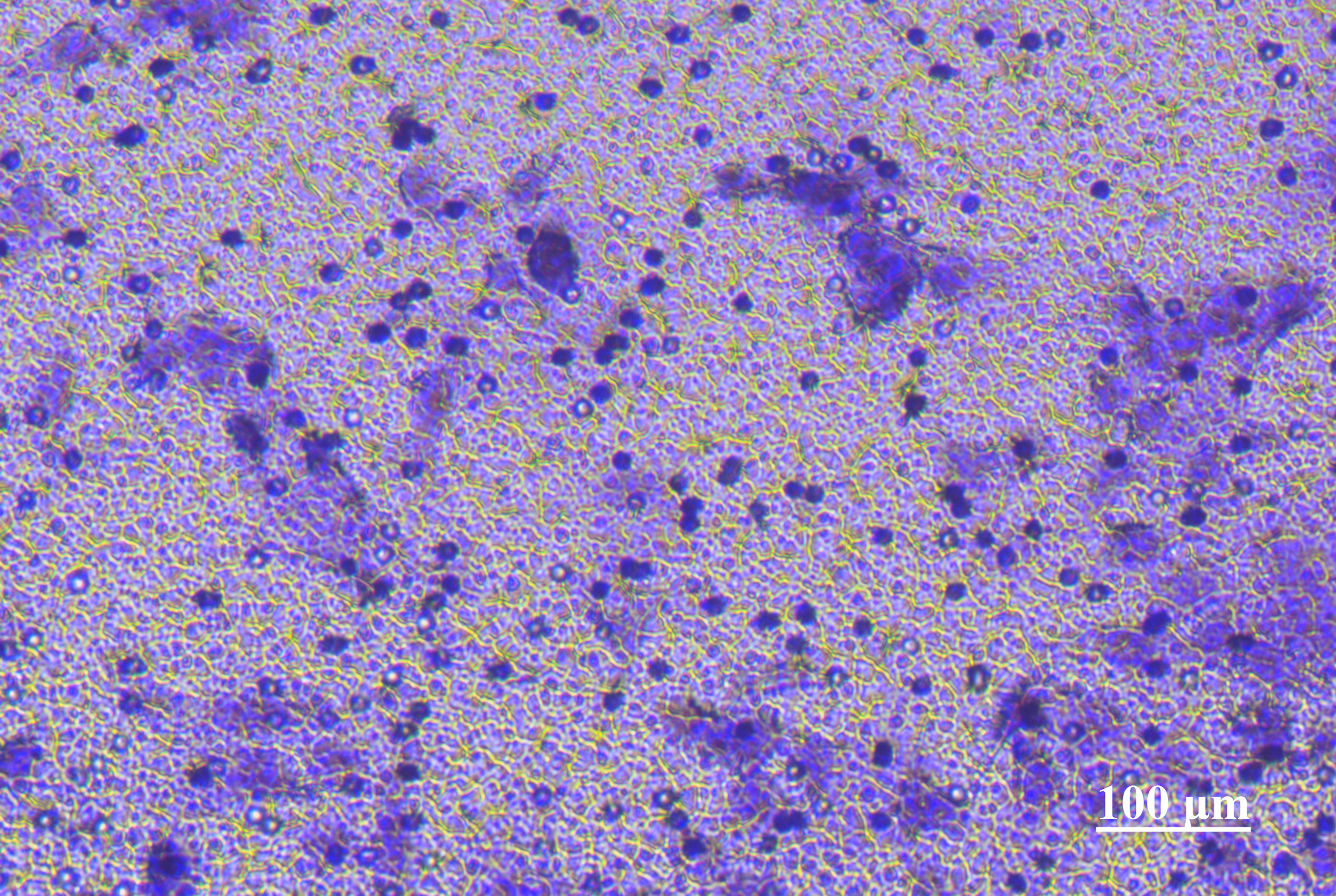

Supplement: Supplementary file 4 — Supplementary Material 4 [file 12885_2024_12140_MOESM4_ESM.zip › Fig.2/2F/U-CH1/MI-2-1-2.jpg]

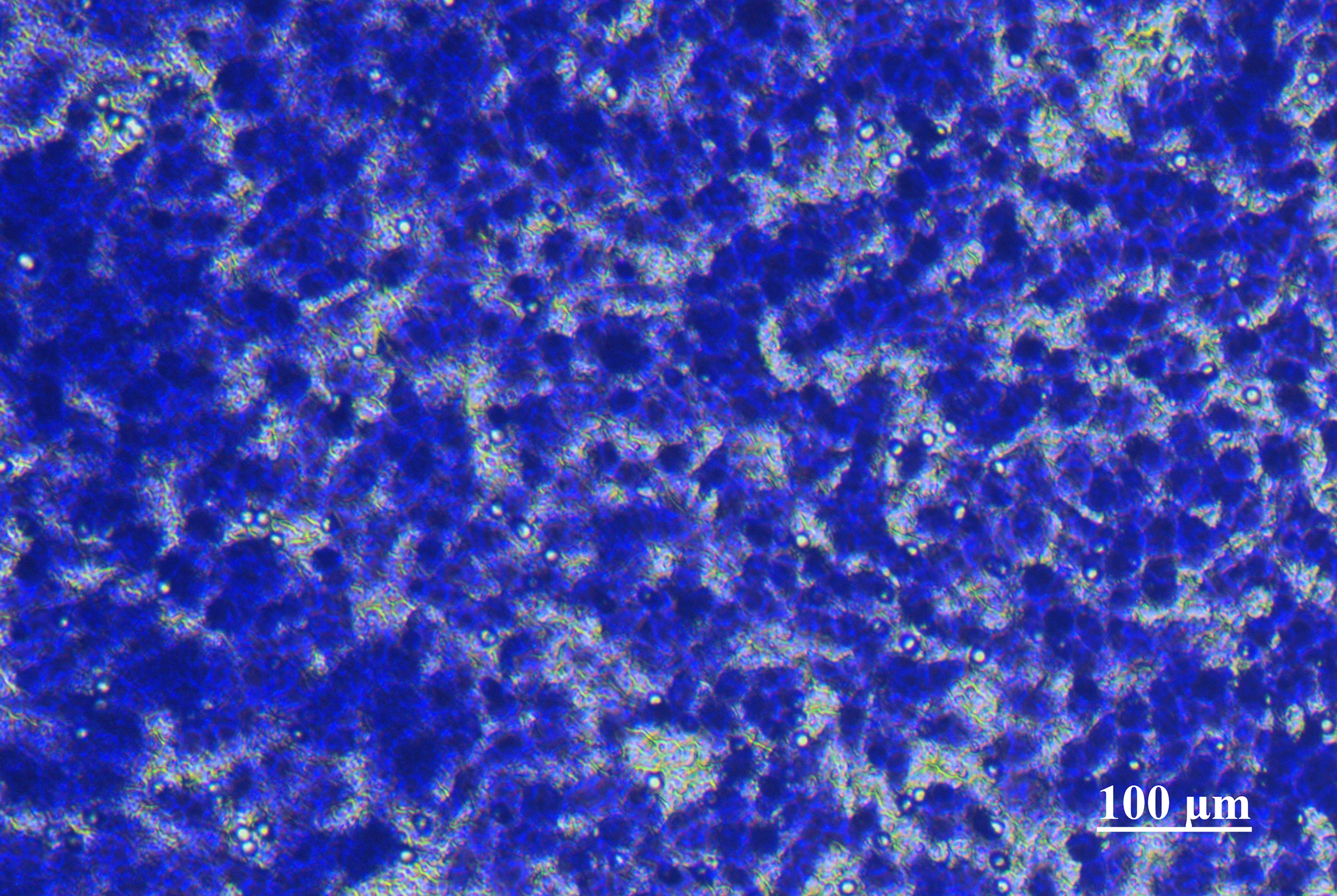

Supplement: Supplementary file 4 — Supplementary Material 4 [file 12885_2024_12140_MOESM4_ESM.zip › Fig.2/2F/U-CH2/MI-2-2-1.jpg]

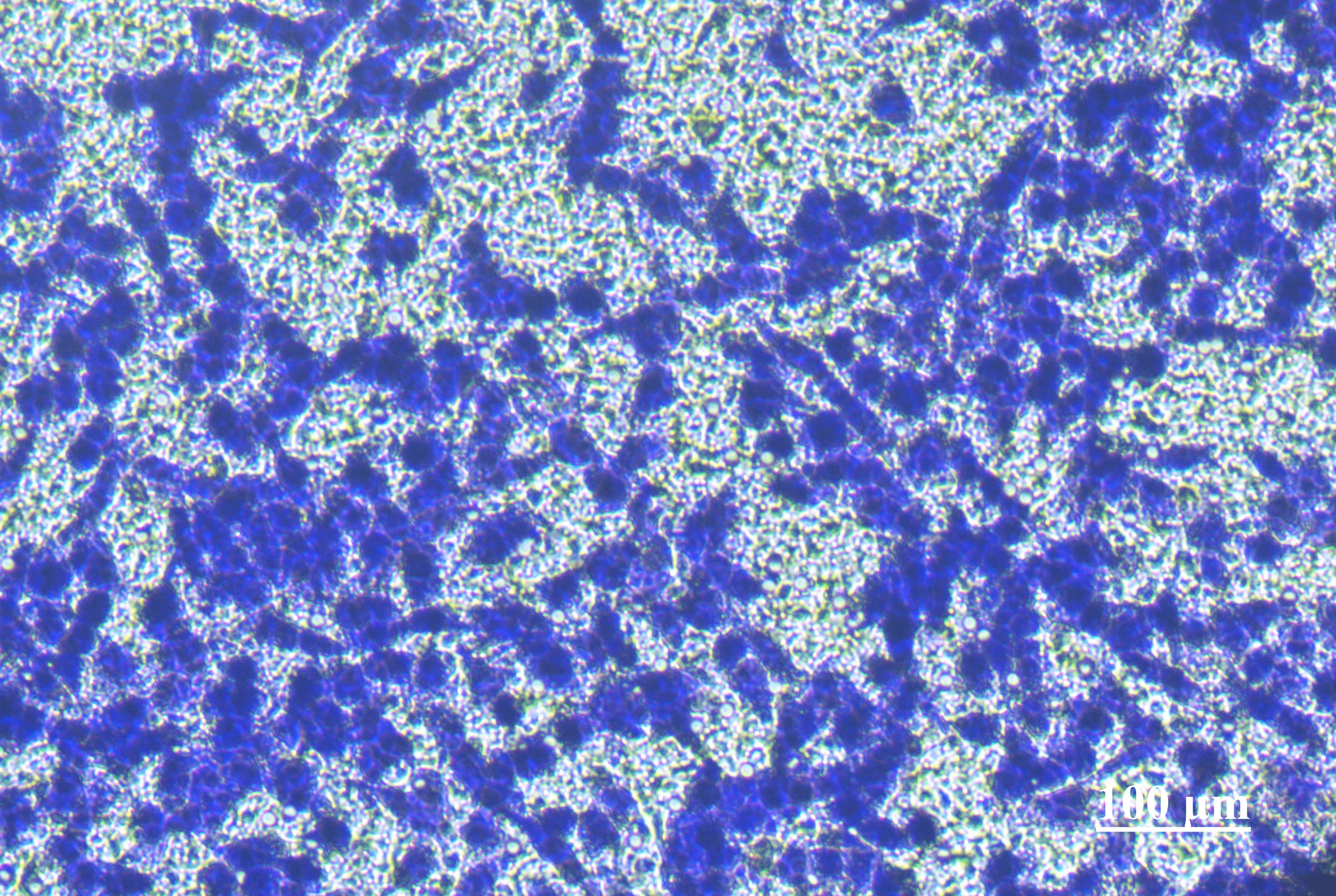

Supplement: Supplementary file 4 — Supplementary Material 4 [file 12885_2024_12140_MOESM4_ESM.zip › Fig.2/2F/U-CH2/MI-2-2-2.jpg]

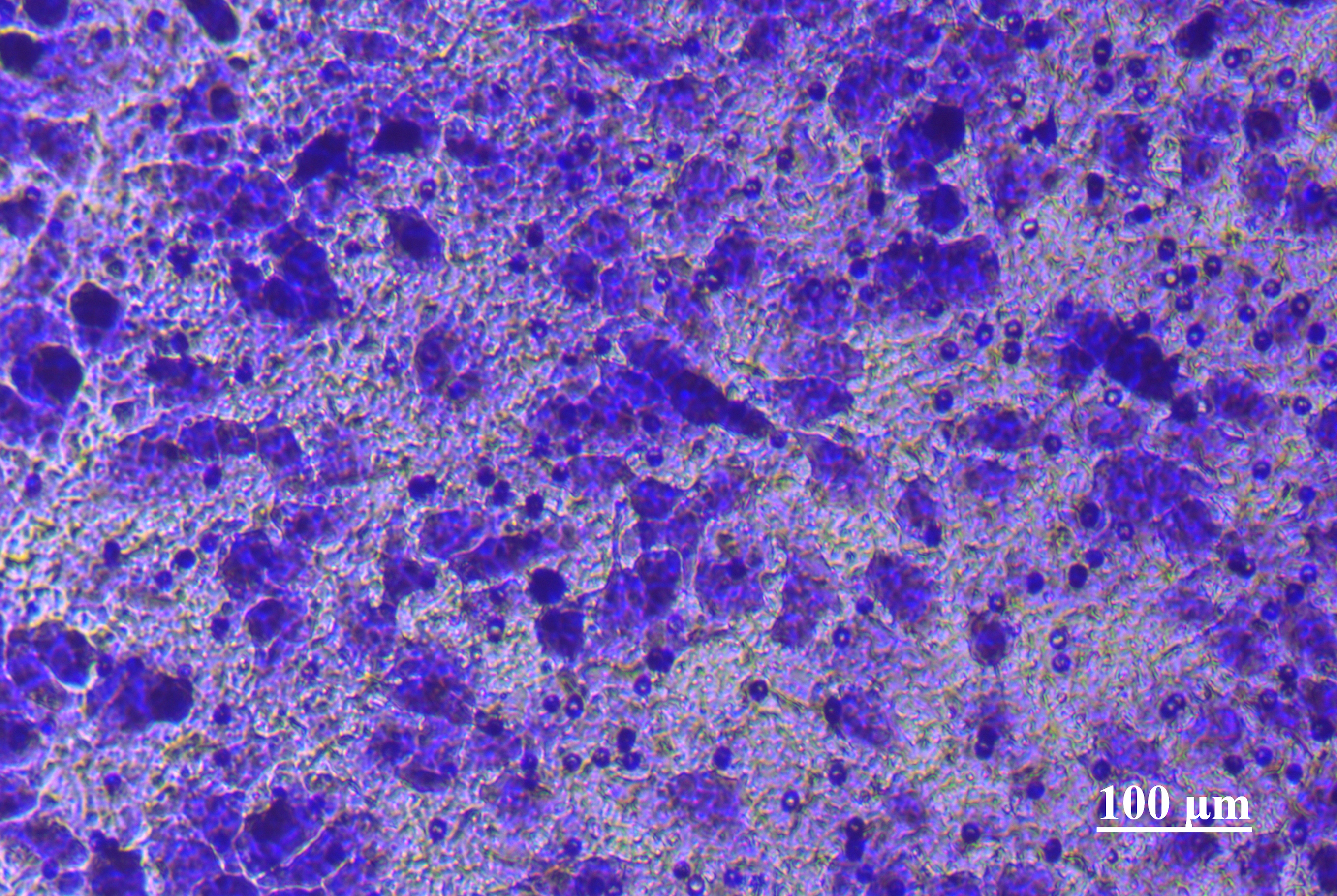

Supplement: Supplementary file 4 — Supplementary Material 4 [file 12885_2024_12140_MOESM4_ESM.zip › Fig.2/2H/U-CH1/IN-2-1-1.jpg]

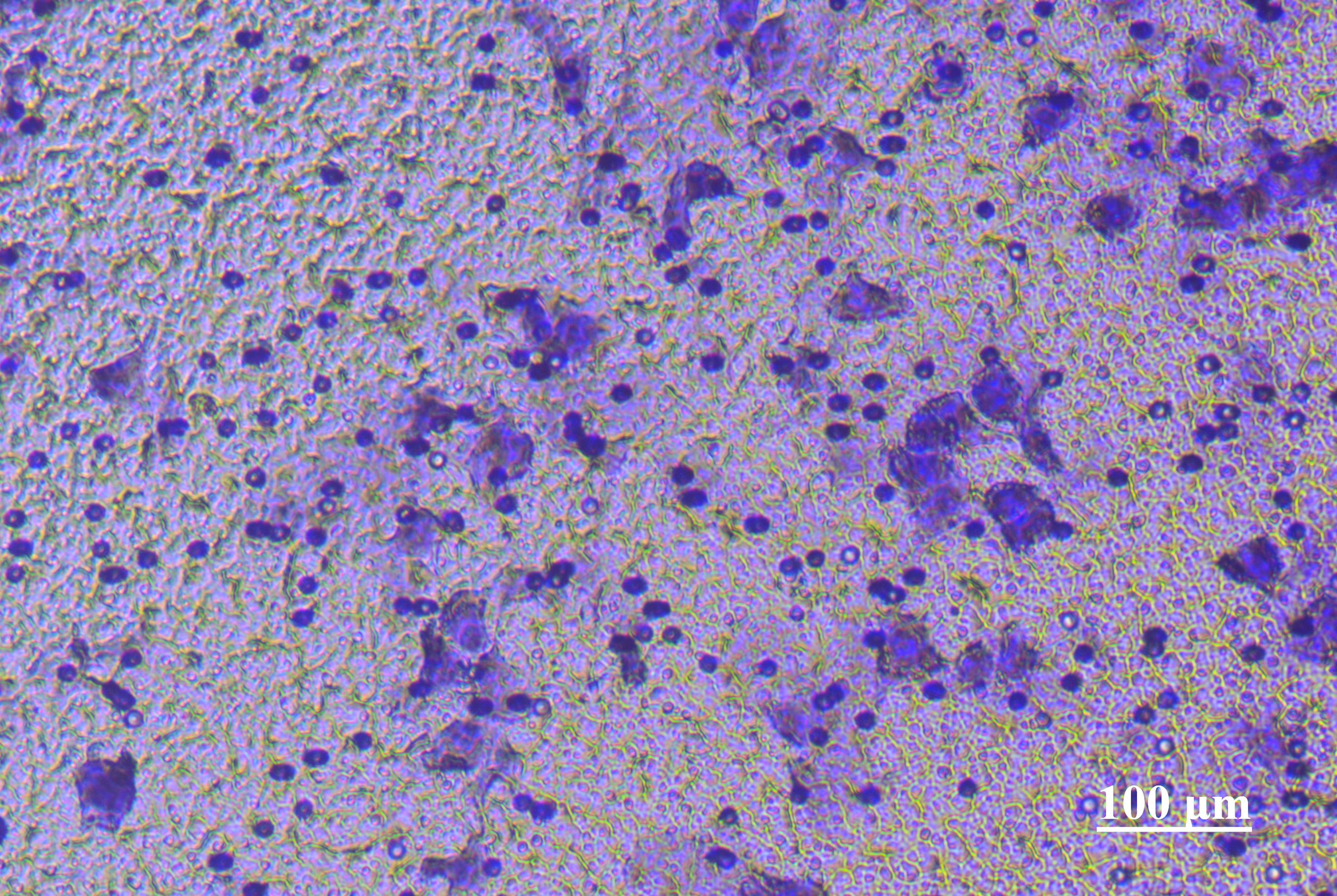

Supplement: Supplementary file 4 — Supplementary Material 4 [file 12885_2024_12140_MOESM4_ESM.zip › Fig.2/2H/U-CH1/IN-2-1-2.jpg]

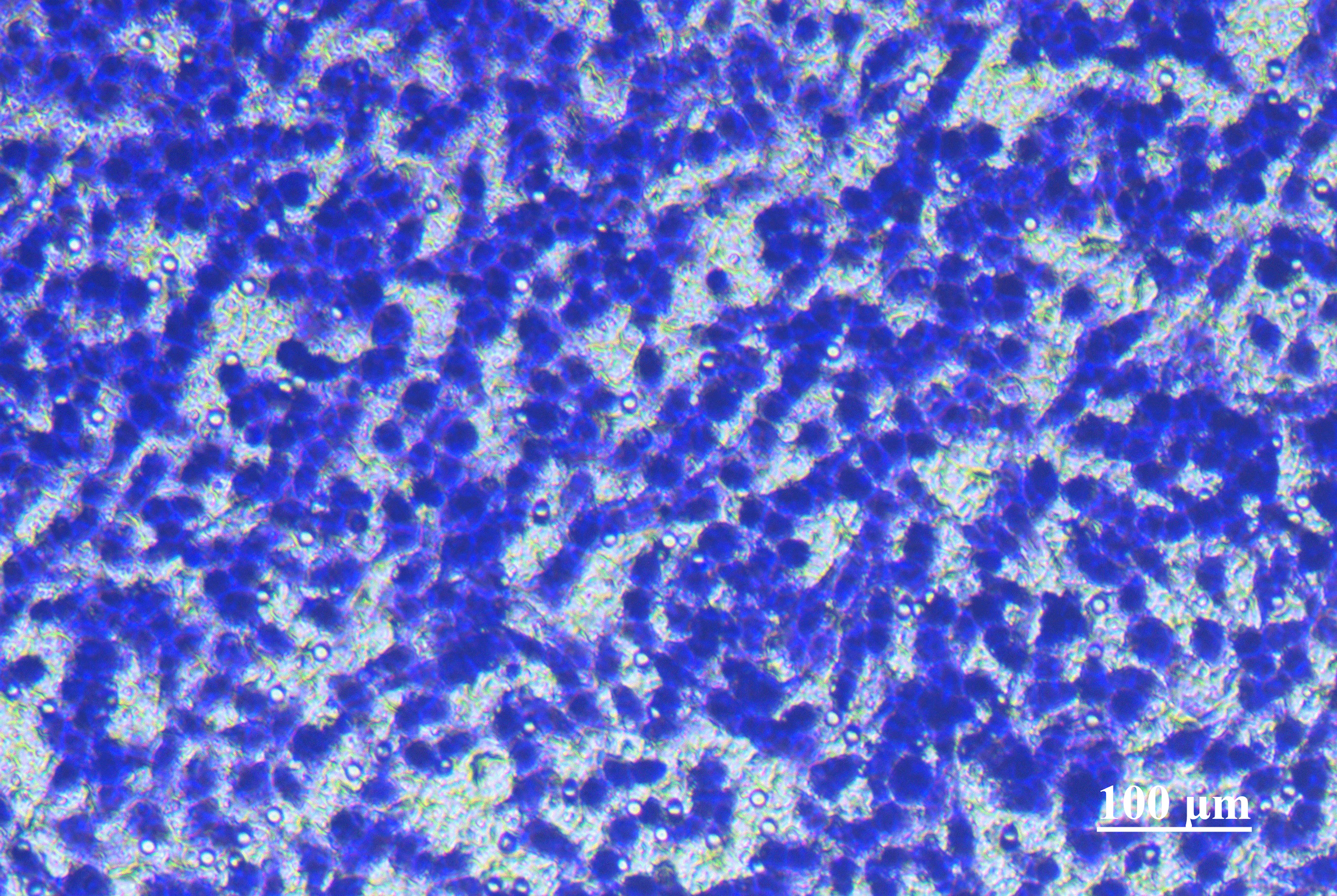

Supplement: Supplementary file 4 — Supplementary Material 4 [file 12885_2024_12140_MOESM4_ESM.zip › Fig.2/2H/U-CH2/IN-2-2-1.jpg]

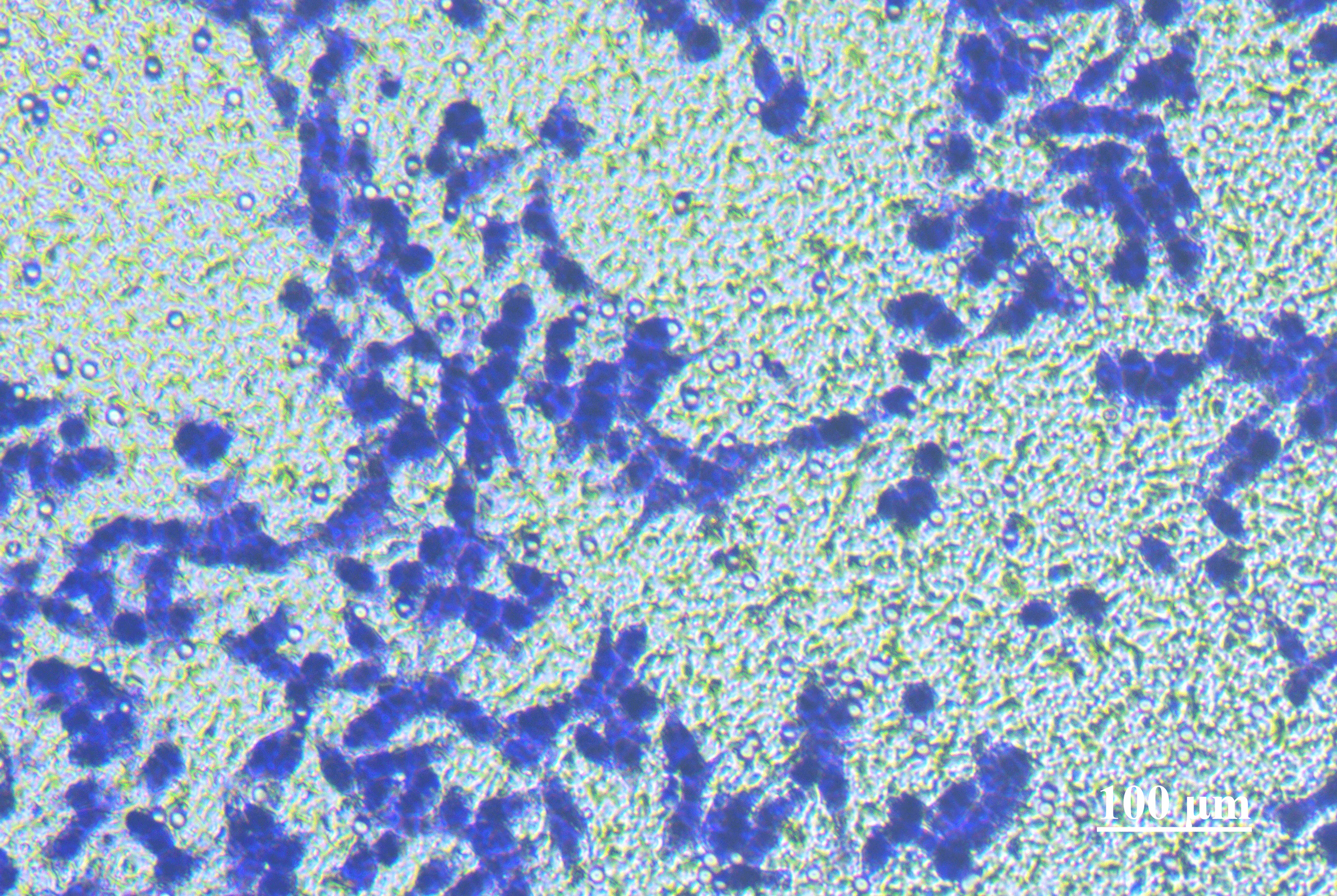

Supplement: Supplementary file 4 — Supplementary Material 4 [file 12885_2024_12140_MOESM4_ESM.zip › Fig.2/2H/U-CH2/IN-2-2-2.jpg]

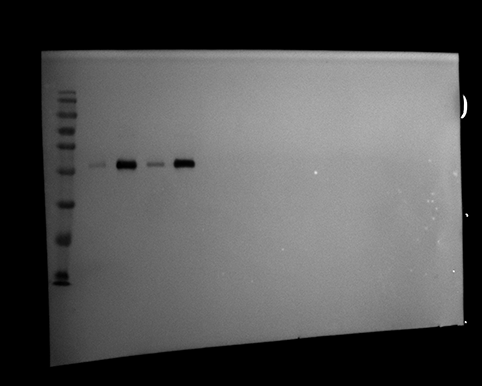

Supplement: Supplementary file 5 — Supplementary Material 5 [file 12885_2024_12140_MOESM5_ESM.zip › Fig.4/4B/1-c-myc.tif]

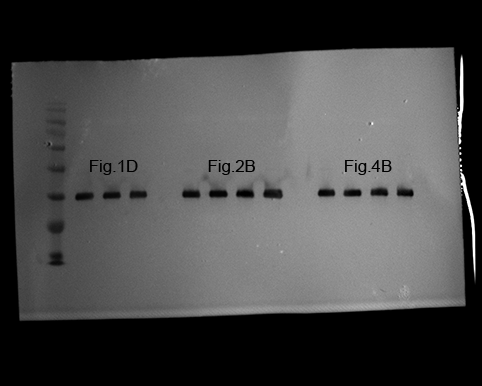

Supplement: Supplementary file 5 — Supplementary Material 5 [file 12885_2024_12140_MOESM5_ESM.zip › Fig.4/4B/2-GAPDH.tif]

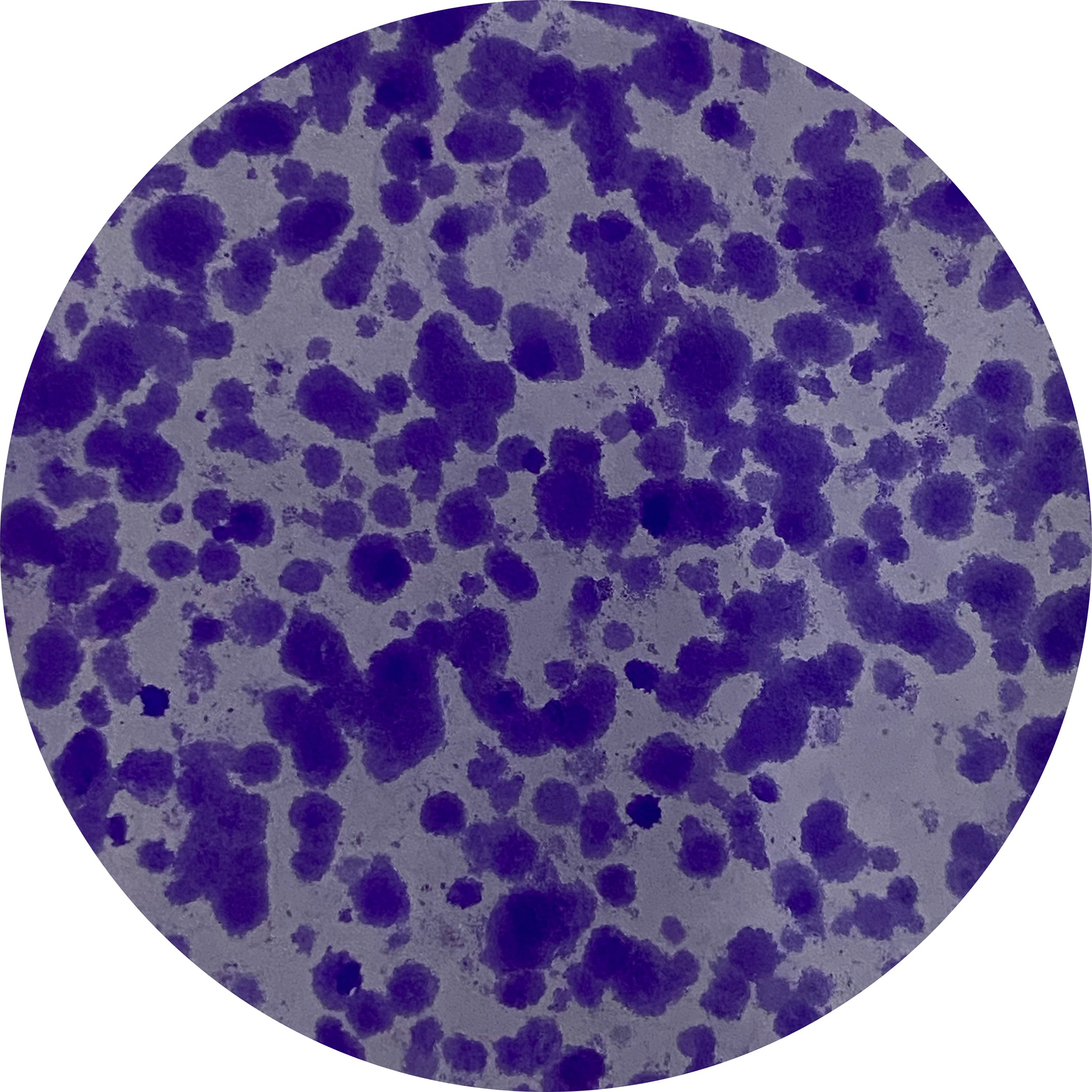

Supplement: Supplementary file 5 — Supplementary Material 5 [file 12885_2024_12140_MOESM5_ESM.zip › Fig.4/4D/U-CH1/4-1-1.jpg]

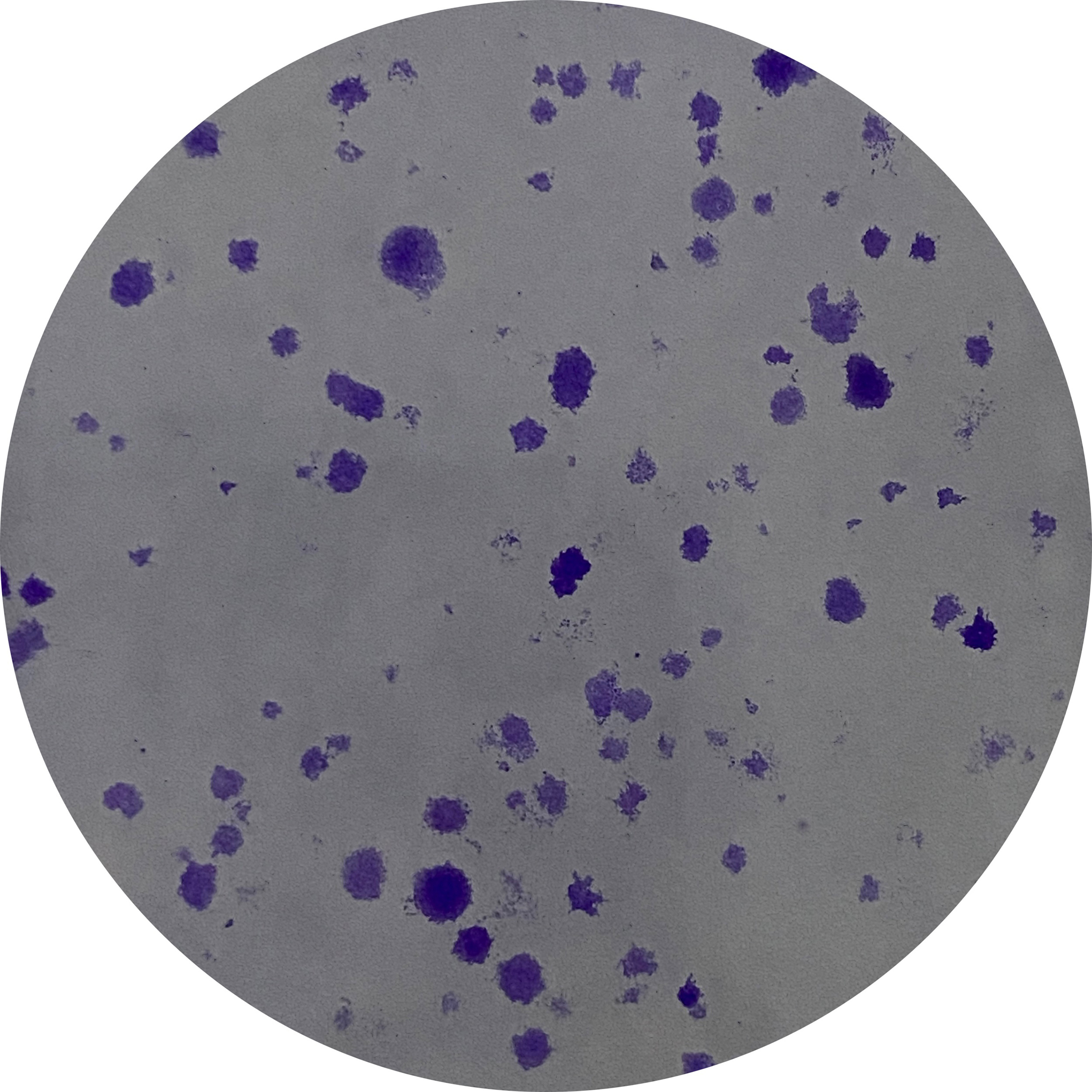

Supplement: Supplementary file 5 — Supplementary Material 5 [file 12885_2024_12140_MOESM5_ESM.zip › Fig.4/4D/U-CH1/4-1-2.jpg]

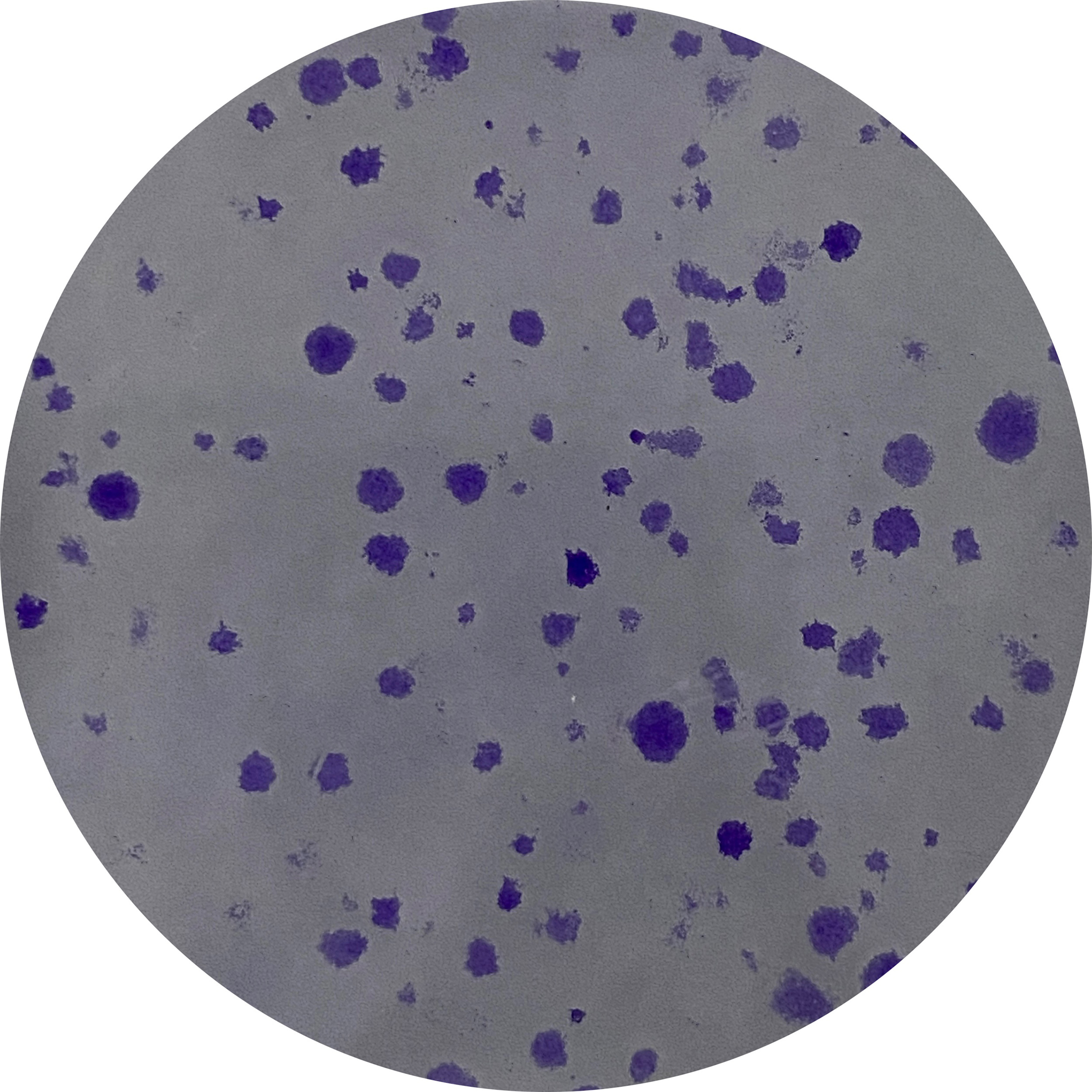

Supplement: Supplementary file 5 — Supplementary Material 5 [file 12885_2024_12140_MOESM5_ESM.zip › Fig.4/4D/U-CH1/4-1-3.jpg]

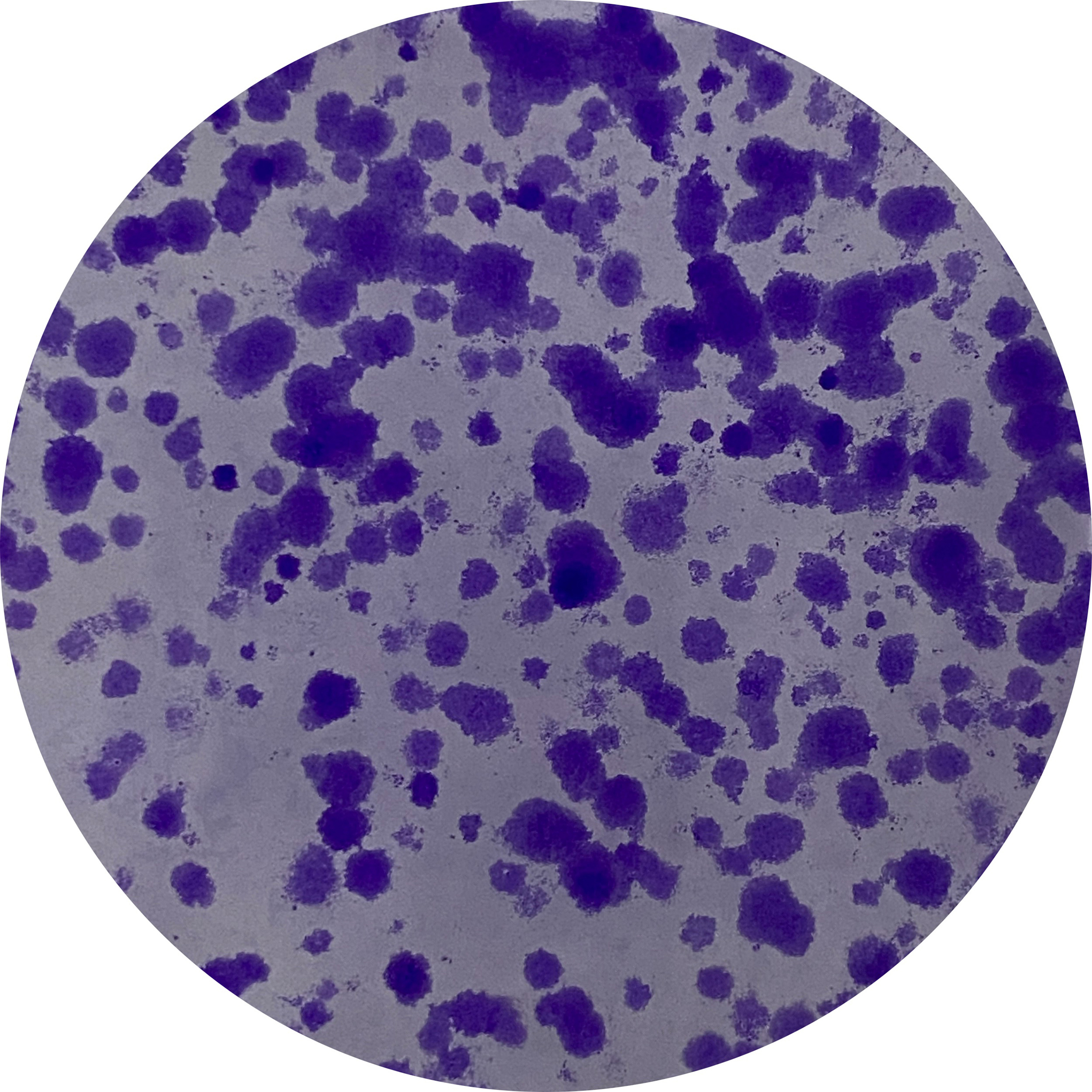

Supplement: Supplementary file 5 — Supplementary Material 5 [file 12885_2024_12140_MOESM5_ESM.zip › Fig.4/4D/U-CH1/4-1-4.jpg]

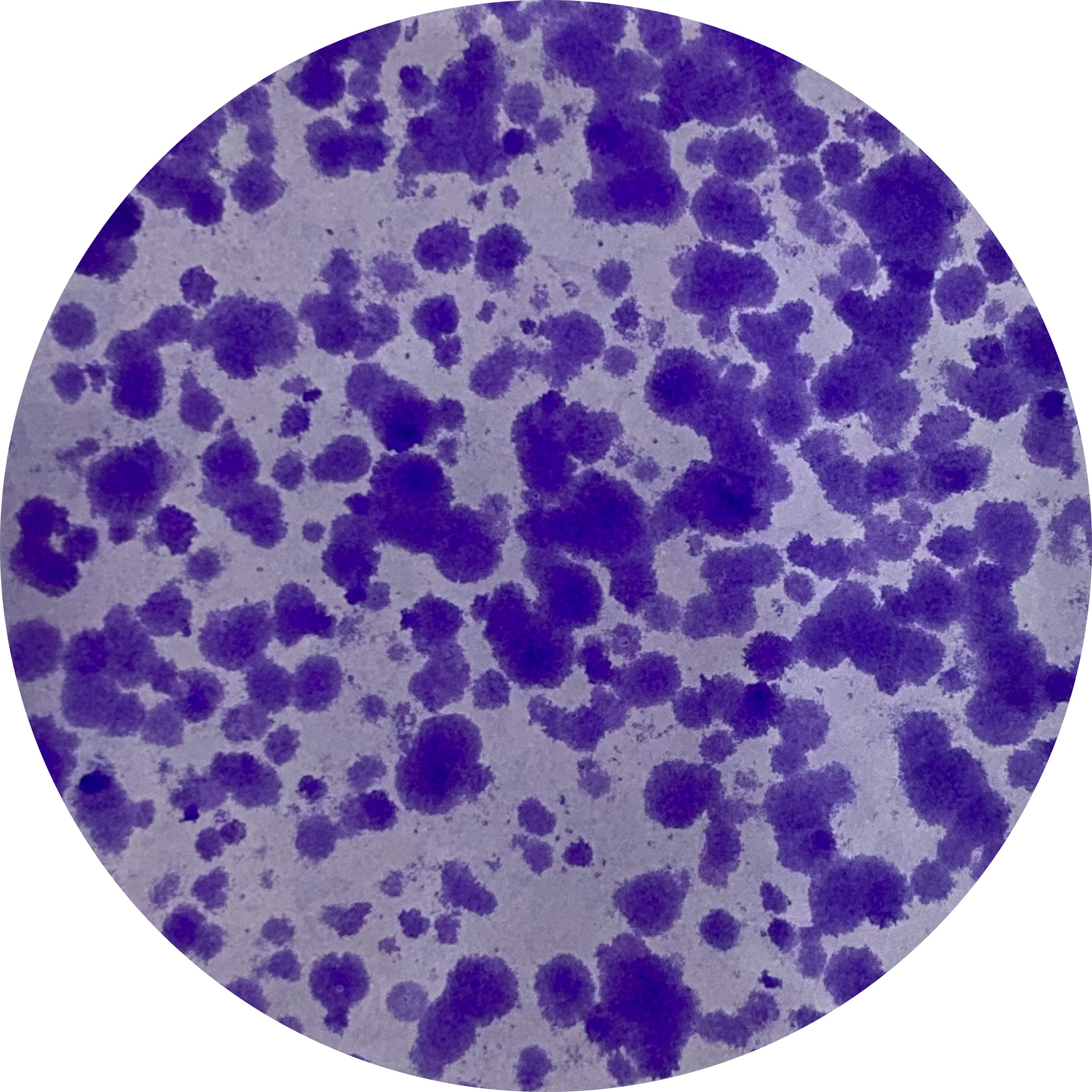

Supplement: Supplementary file 5 — Supplementary Material 5 [file 12885_2024_12140_MOESM5_ESM.zip › Fig.4/4D/U-CH2/4-2-1.jpg]

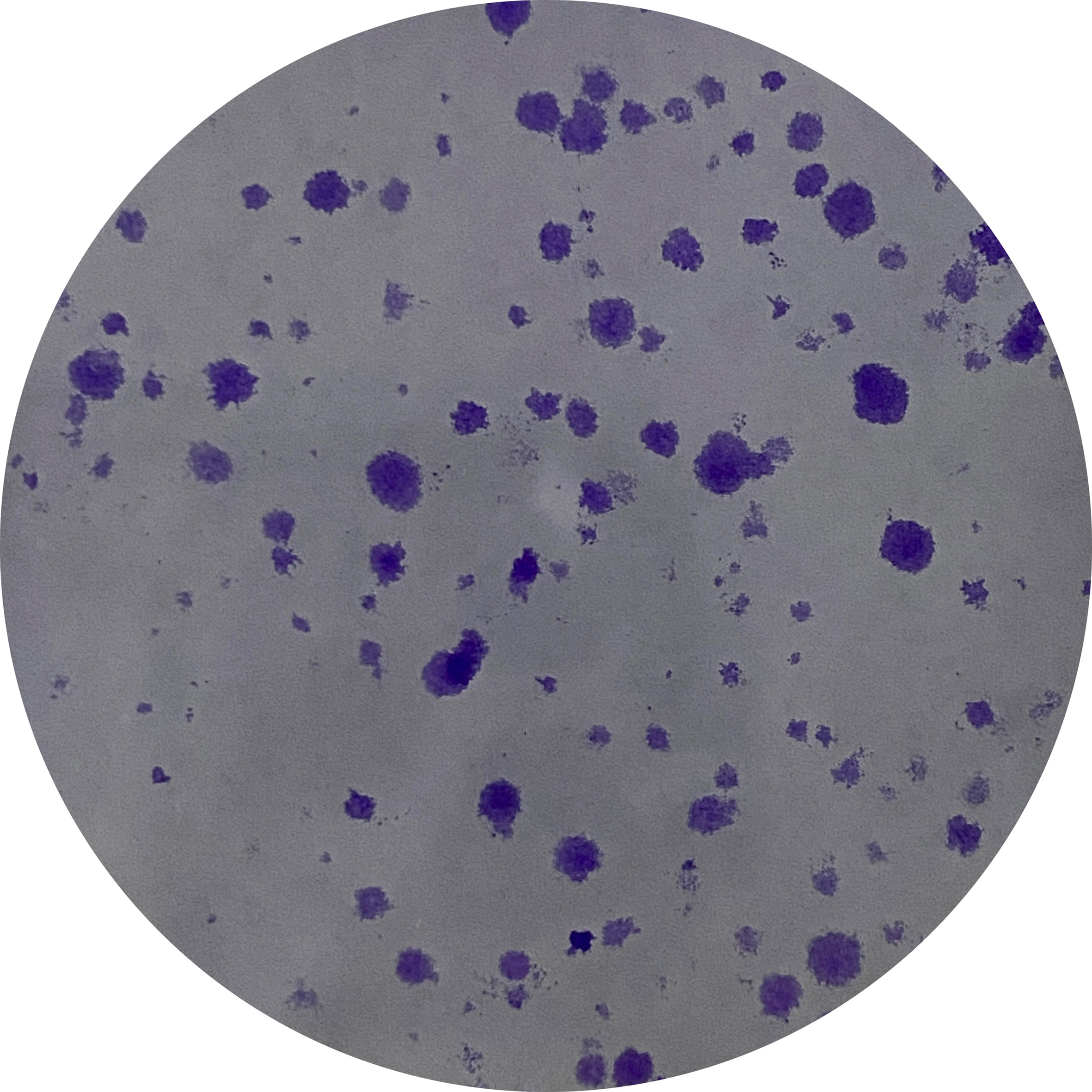

Supplement: Supplementary file 5 — Supplementary Material 5 [file 12885_2024_12140_MOESM5_ESM.zip › Fig.4/4D/U-CH2/4-2-2.jpg]

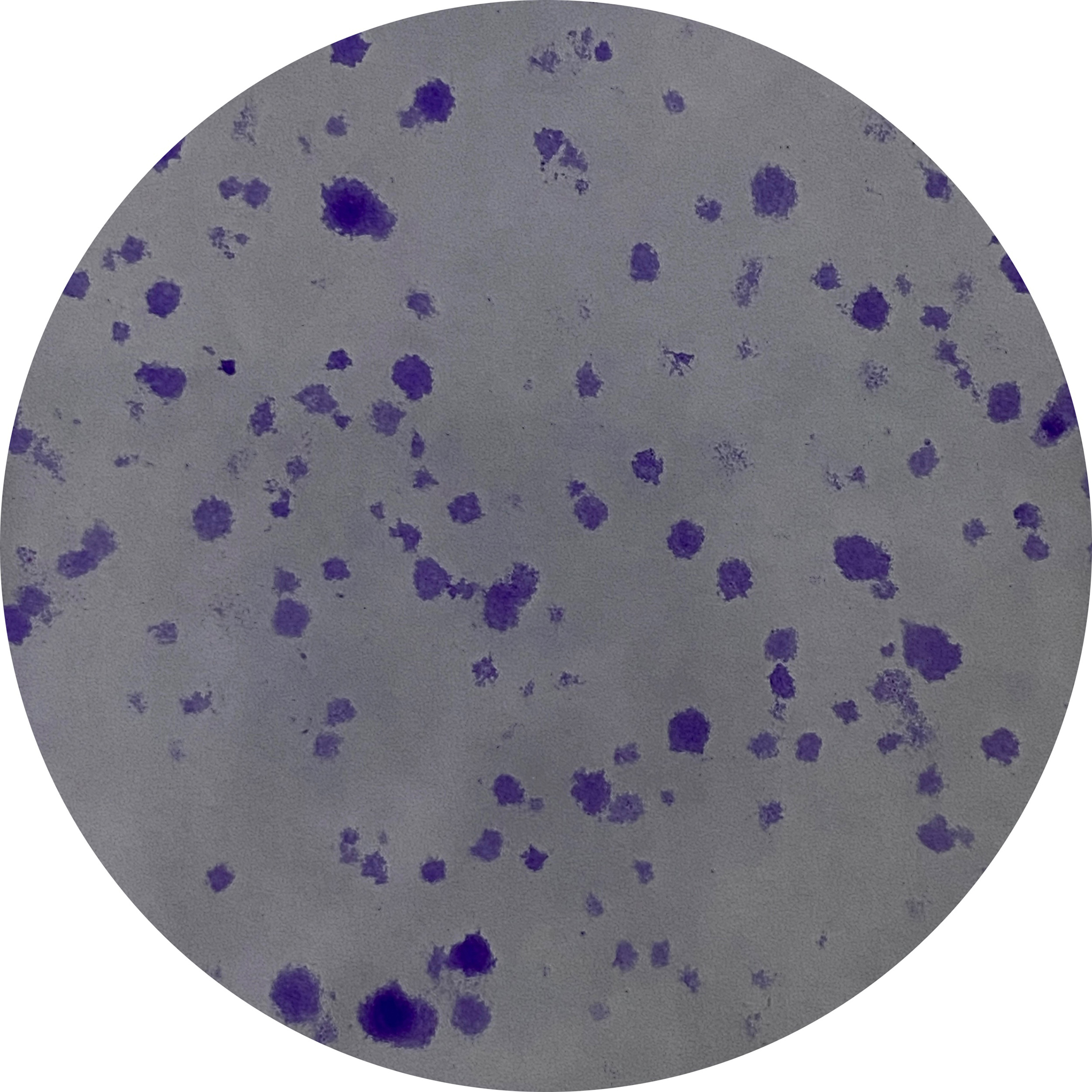

Supplement: Supplementary file 5 — Supplementary Material 5 [file 12885_2024_12140_MOESM5_ESM.zip › Fig.4/4D/U-CH2/4-2-3.jpg]

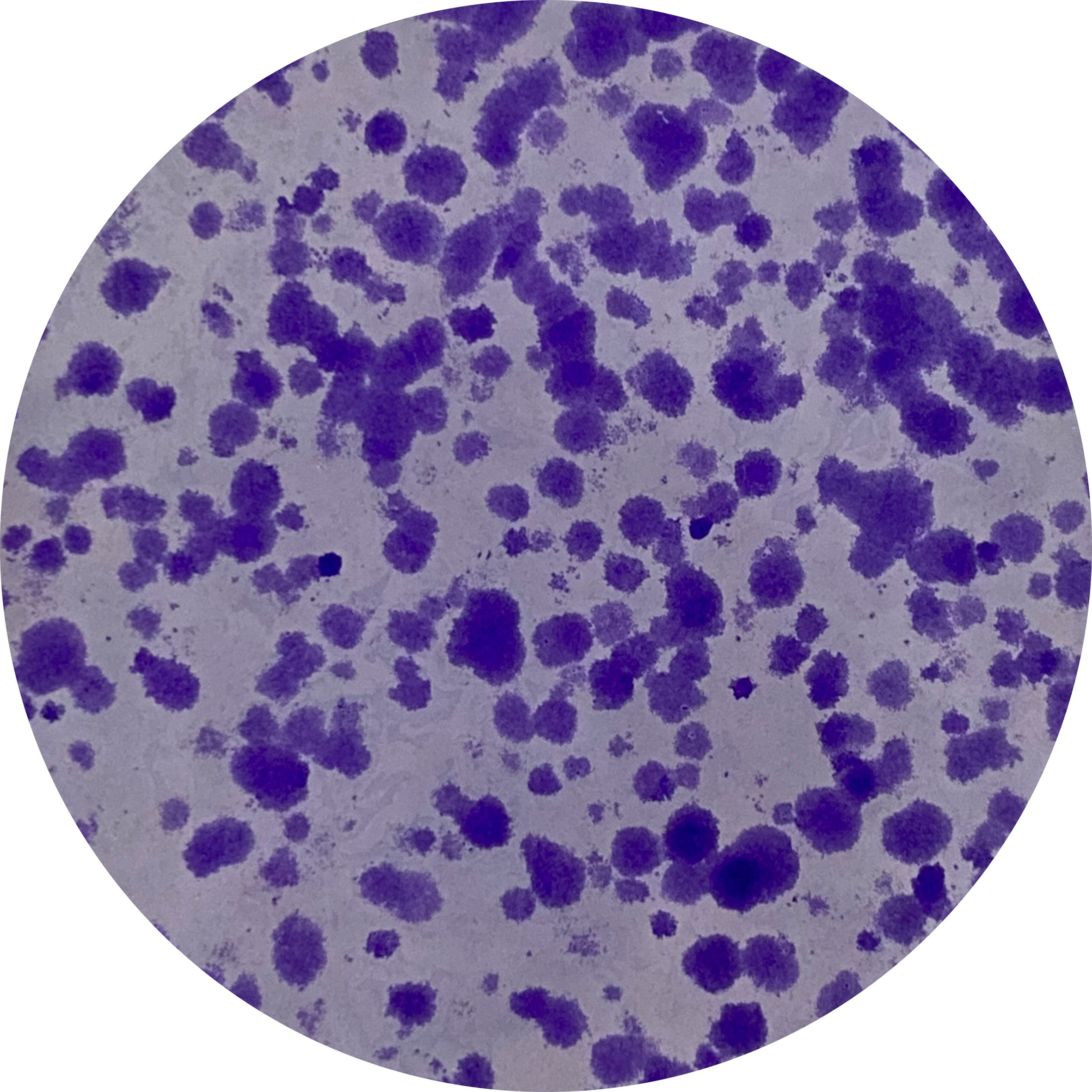

Supplement: Supplementary file 5 — Supplementary Material 5 [file 12885_2024_12140_MOESM5_ESM.zip › Fig.4/4D/U-CH2/4-2-4.jpg]

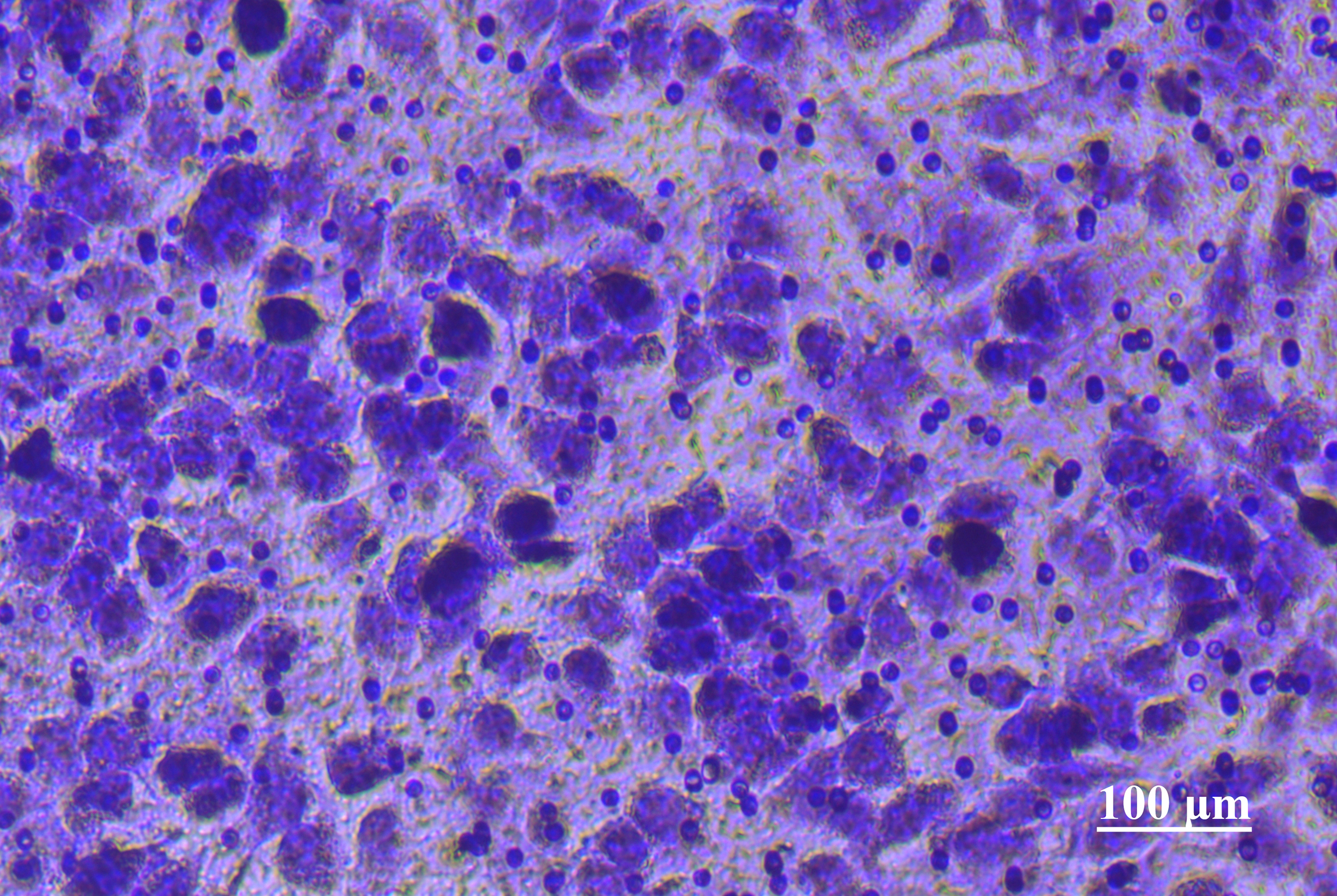

Supplement: Supplementary file 5 — Supplementary Material 5 [file 12885_2024_12140_MOESM5_ESM.zip › Fig.4/4F/U-CH1/MI-4-1-1.jpg]

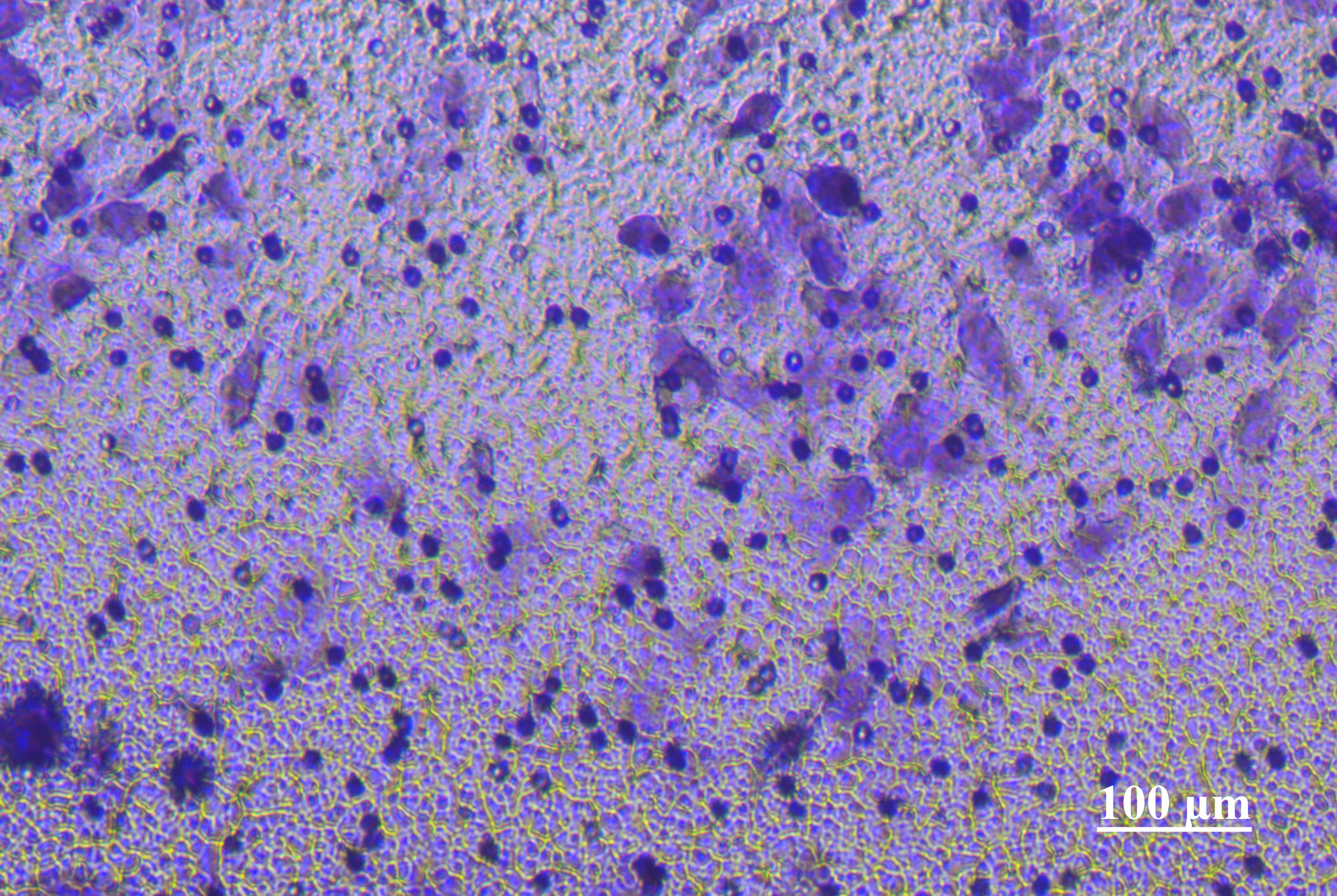

Supplement: Supplementary file 5 — Supplementary Material 5 [file 12885_2024_12140_MOESM5_ESM.zip › Fig.4/4F/U-CH1/MI-4-1-2.jpg]

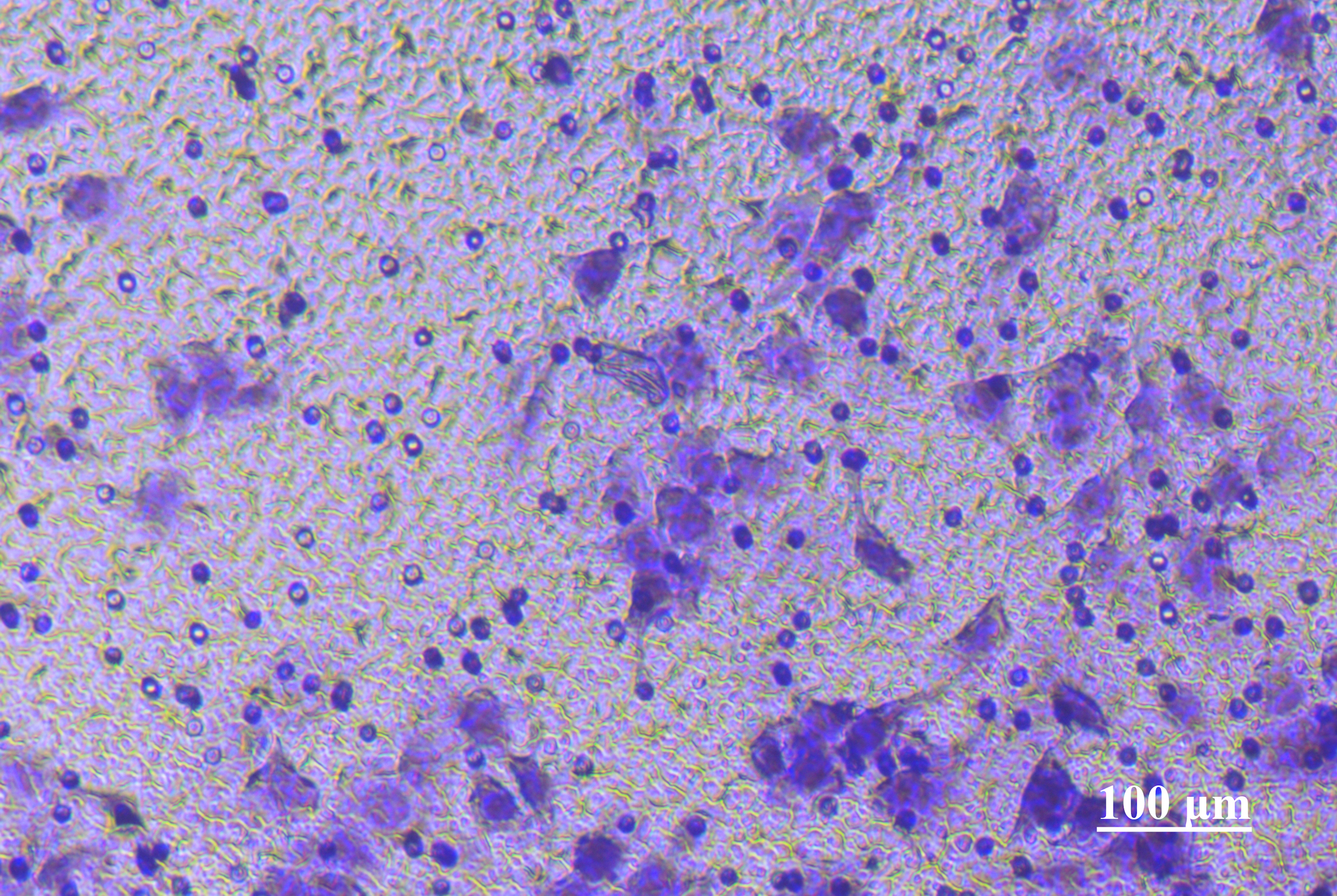

Supplement: Supplementary file 5 — Supplementary Material 5 [file 12885_2024_12140_MOESM5_ESM.zip › Fig.4/4F/U-CH1/MI-4-1-3.jpg]

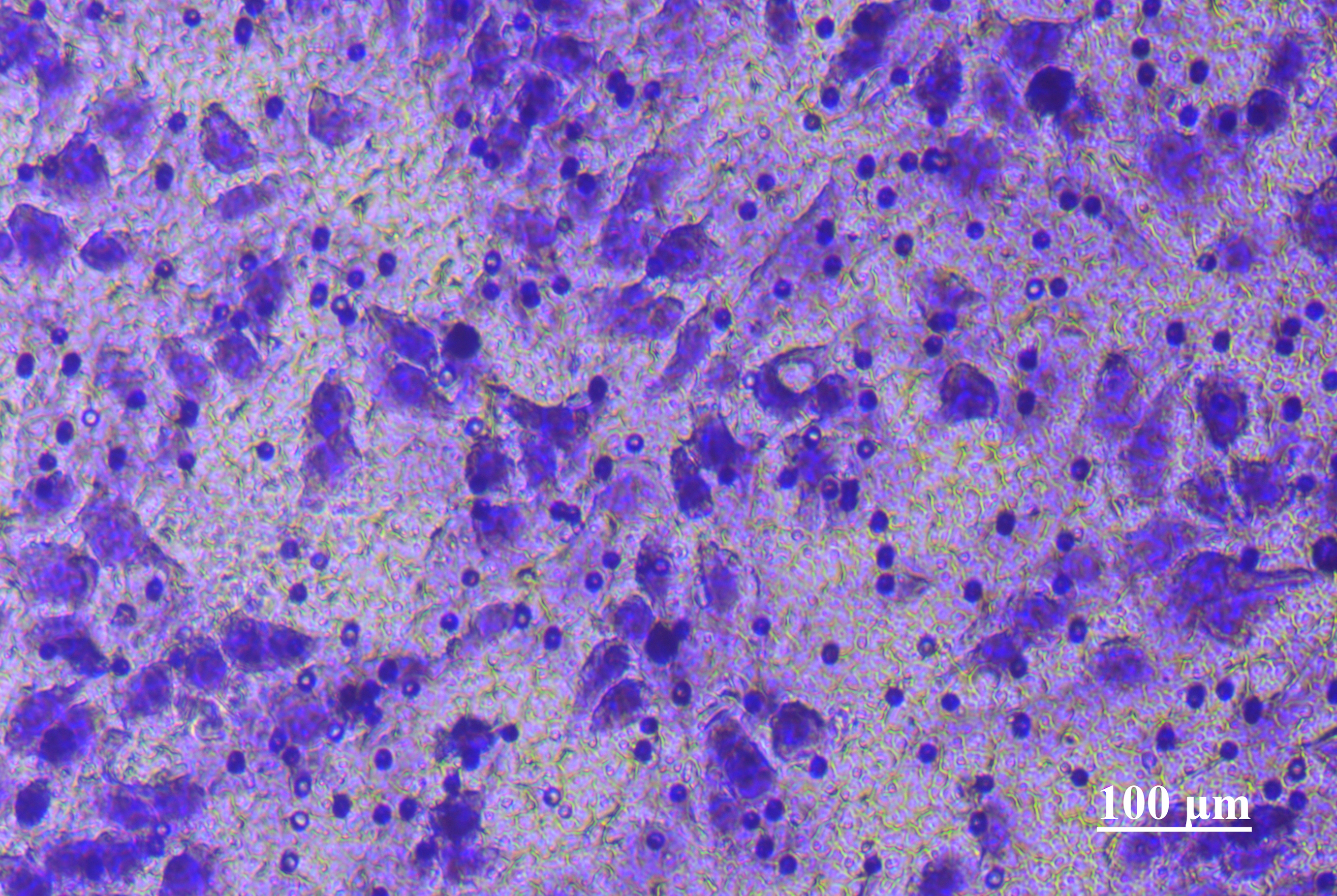

Supplement: Supplementary file 5 — Supplementary Material 5 [file 12885_2024_12140_MOESM5_ESM.zip › Fig.4/4F/U-CH1/MI-4-1-4.jpg]

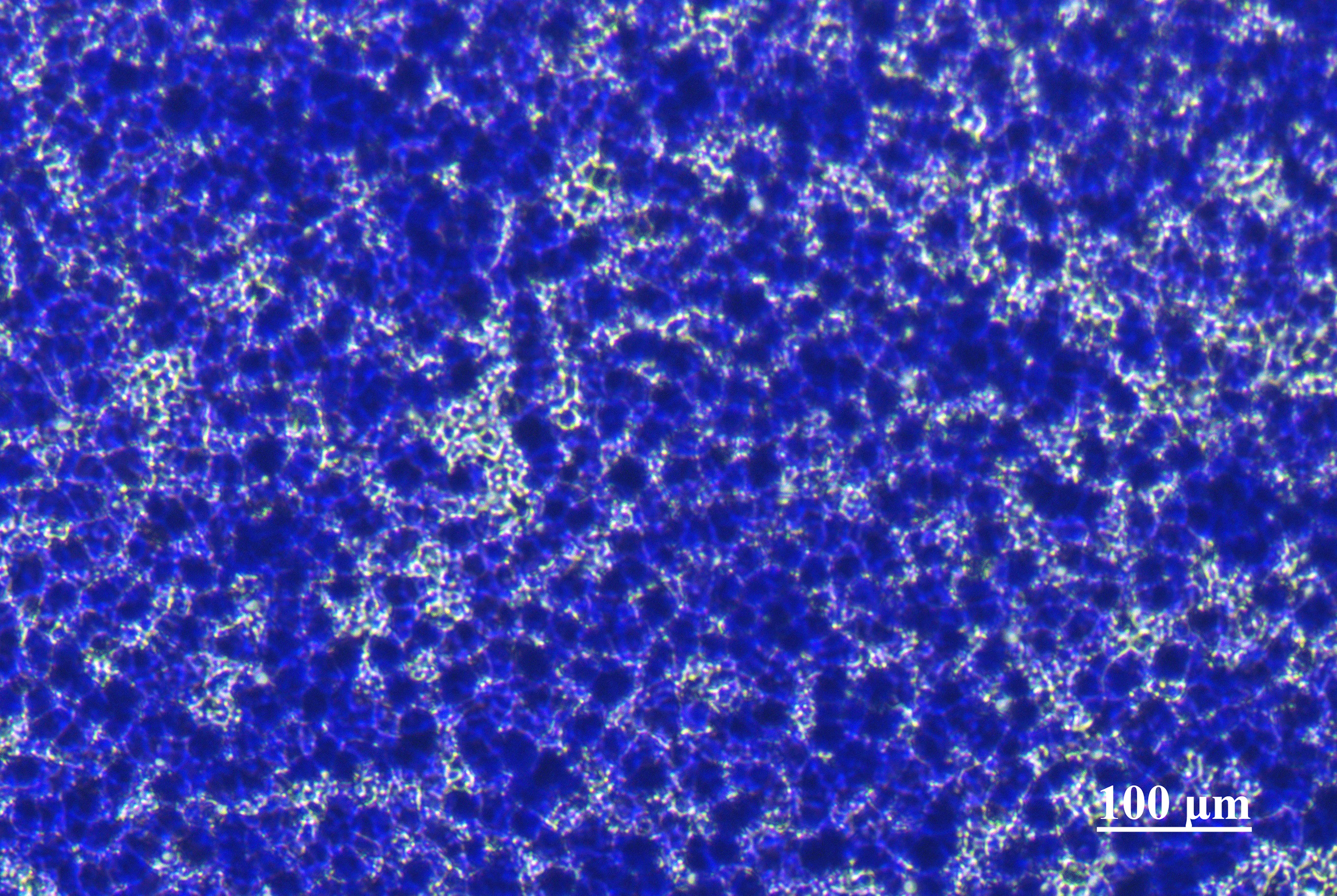

Supplement: Supplementary file 5 — Supplementary Material 5 [file 12885_2024_12140_MOESM5_ESM.zip › Fig.4/4F/U-CH2/MI-4-2-1.jpg]

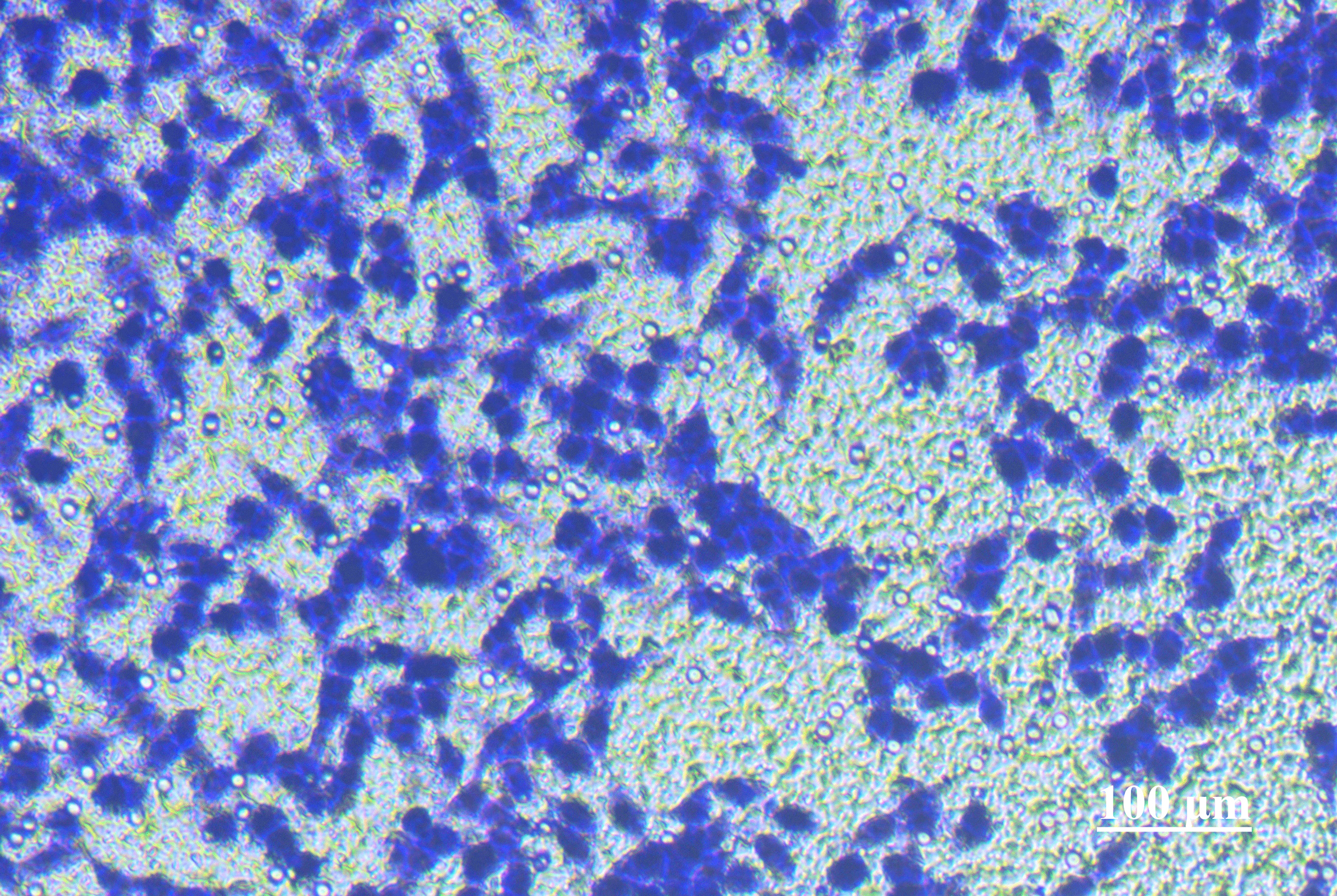

Supplement: Supplementary file 5 — Supplementary Material 5 [file 12885_2024_12140_MOESM5_ESM.zip › Fig.4/4F/U-CH2/MI-4-2-2.jpg]

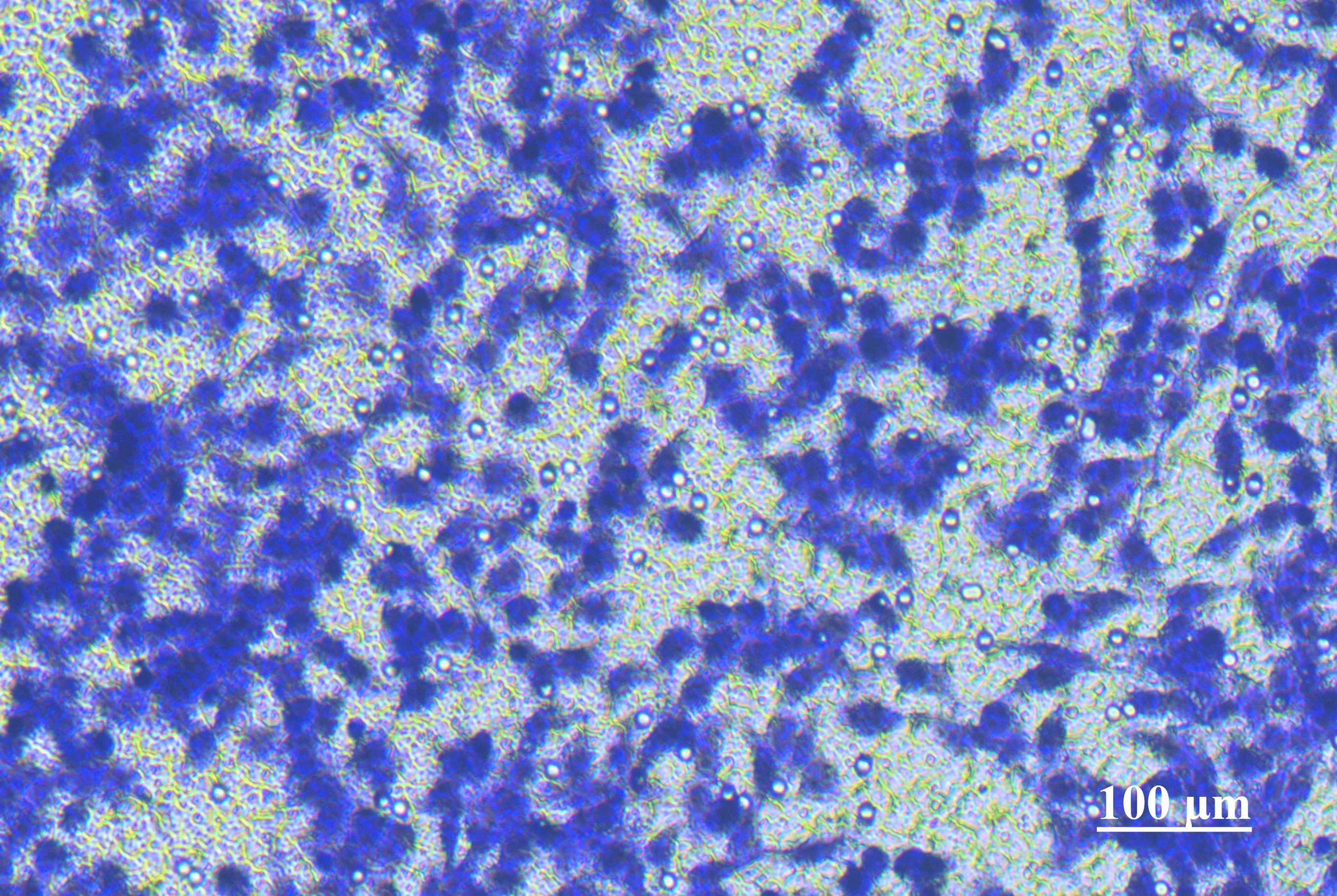

Supplement: Supplementary file 5 — Supplementary Material 5 [file 12885_2024_12140_MOESM5_ESM.zip › Fig.4/4F/U-CH2/MI-4-2-3.jpg]

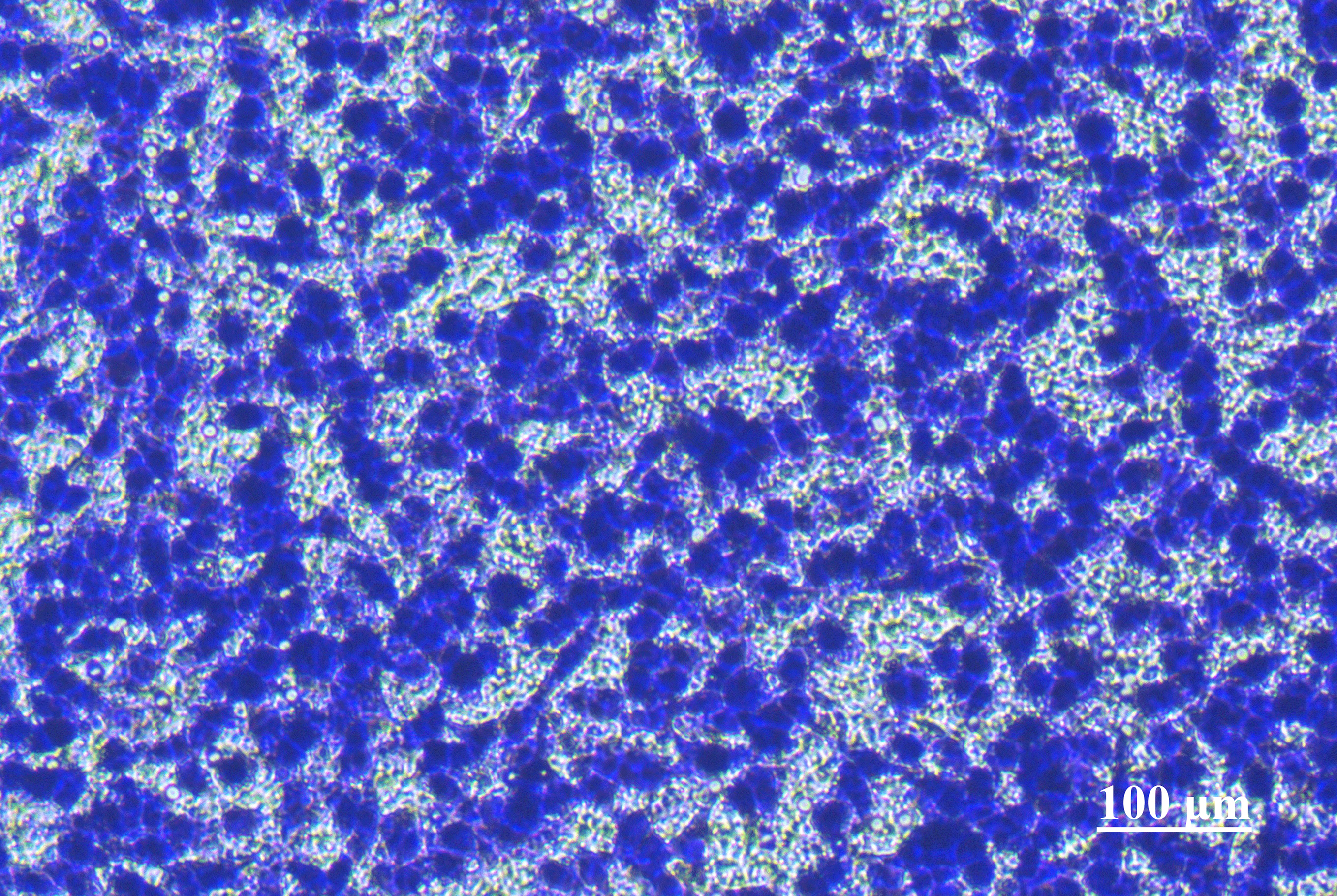

Supplement: Supplementary file 5 — Supplementary Material 5 [file 12885_2024_12140_MOESM5_ESM.zip › Fig.4/4F/U-CH2/MI-4-2-4.jpg]

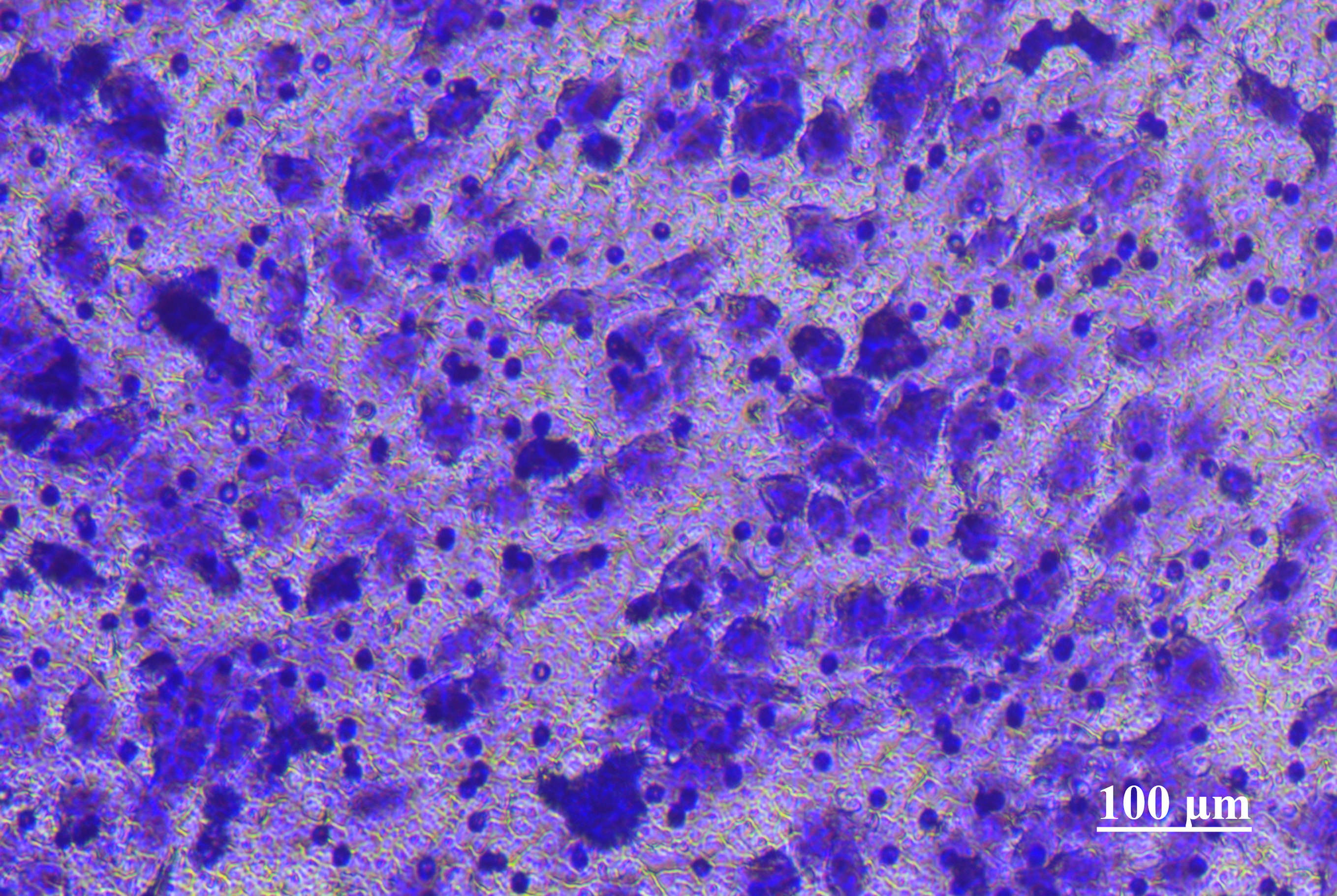

Supplement: Supplementary file 5 — Supplementary Material 5 [file 12885_2024_12140_MOESM5_ESM.zip › Fig.4/4H/U-CH1/IN-4-1-1.jpg]

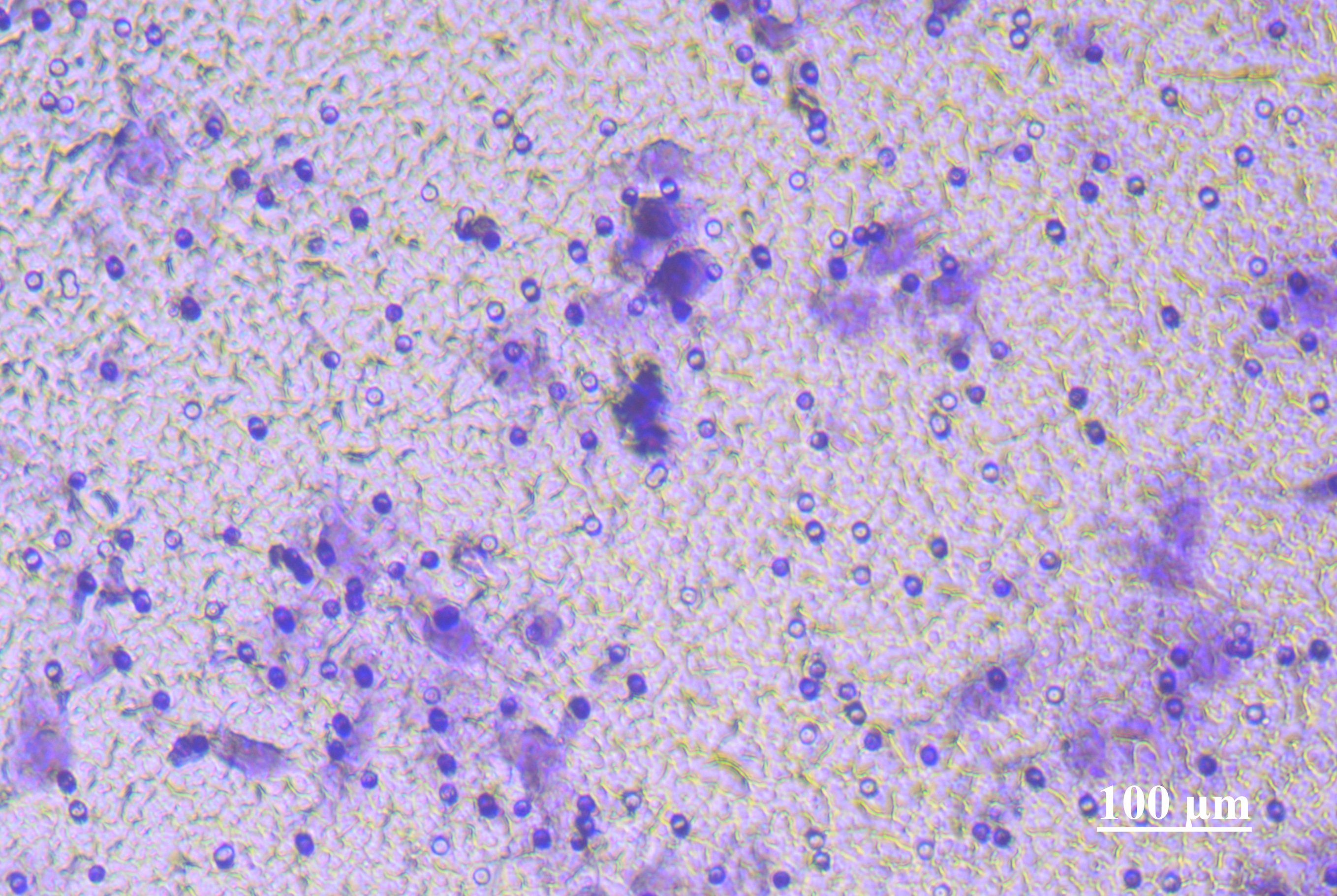

Supplement: Supplementary file 5 — Supplementary Material 5 [file 12885_2024_12140_MOESM5_ESM.zip › Fig.4/4H/U-CH1/IN-4-1-2.jpg]

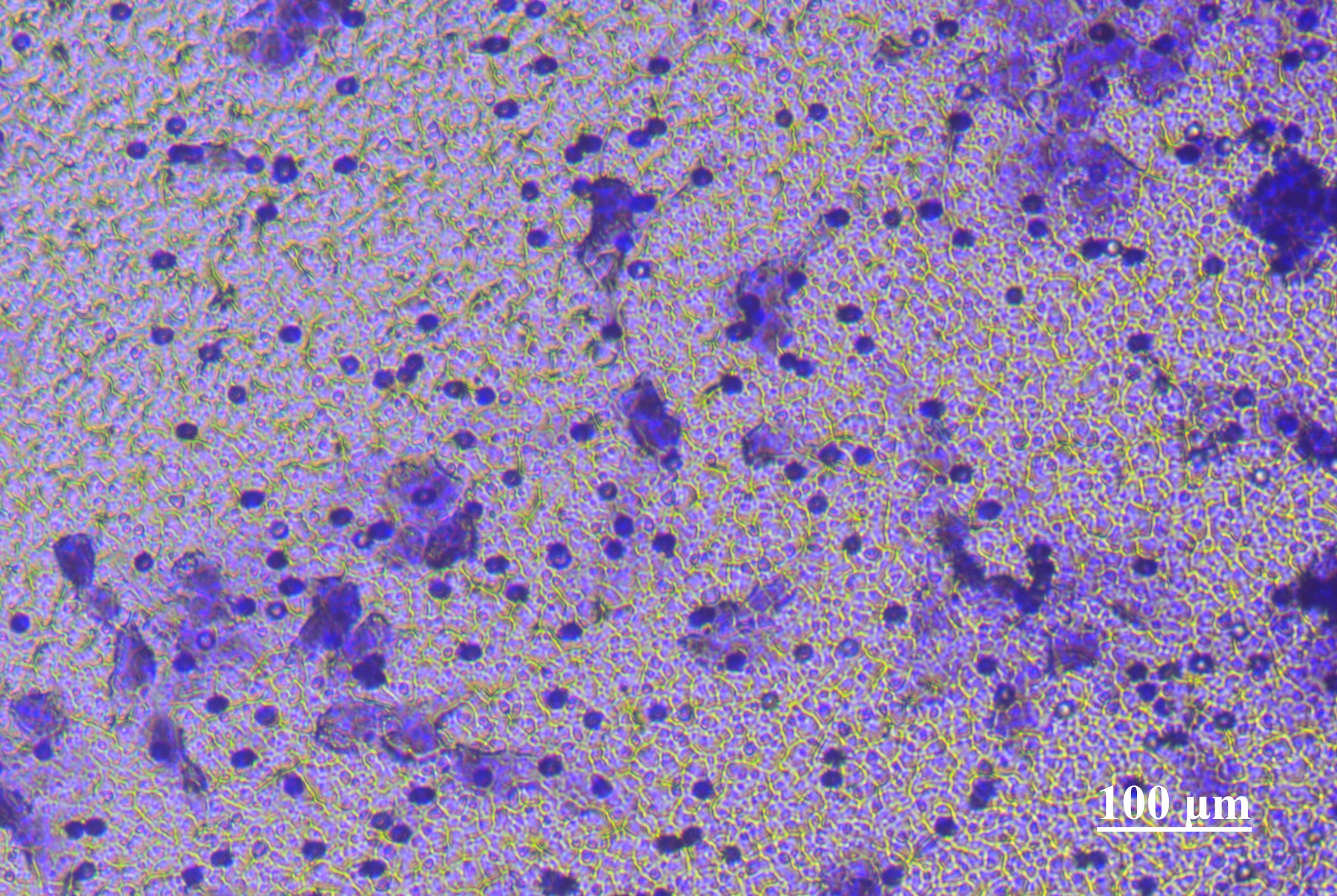

Supplement: Supplementary file 5 — Supplementary Material 5 [file 12885_2024_12140_MOESM5_ESM.zip › Fig.4/4H/U-CH1/IN-4-1-3.jpg]

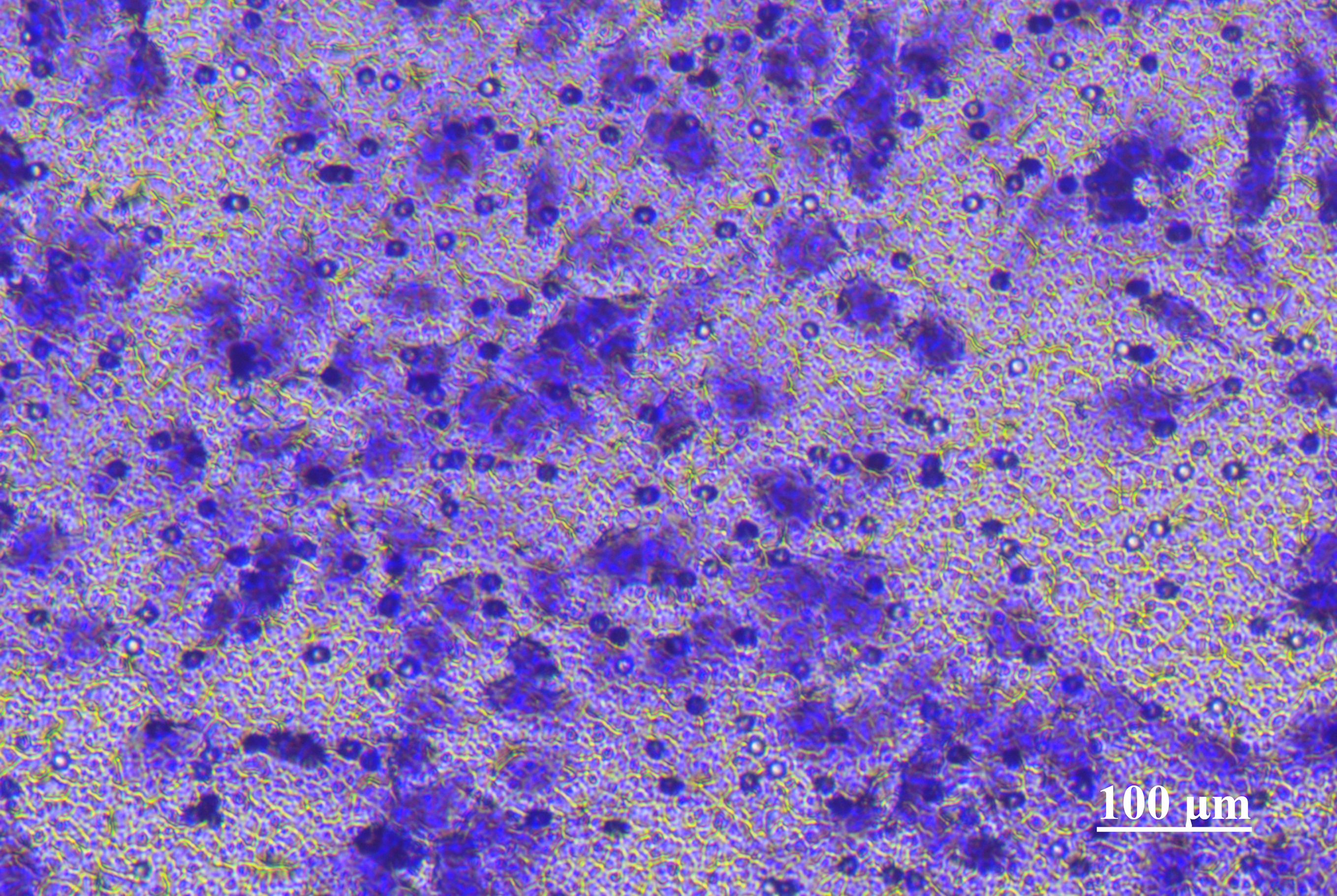

Supplement: Supplementary file 5 — Supplementary Material 5 [file 12885_2024_12140_MOESM5_ESM.zip › Fig.4/4H/U-CH1/IN-4-1-4.jpg]

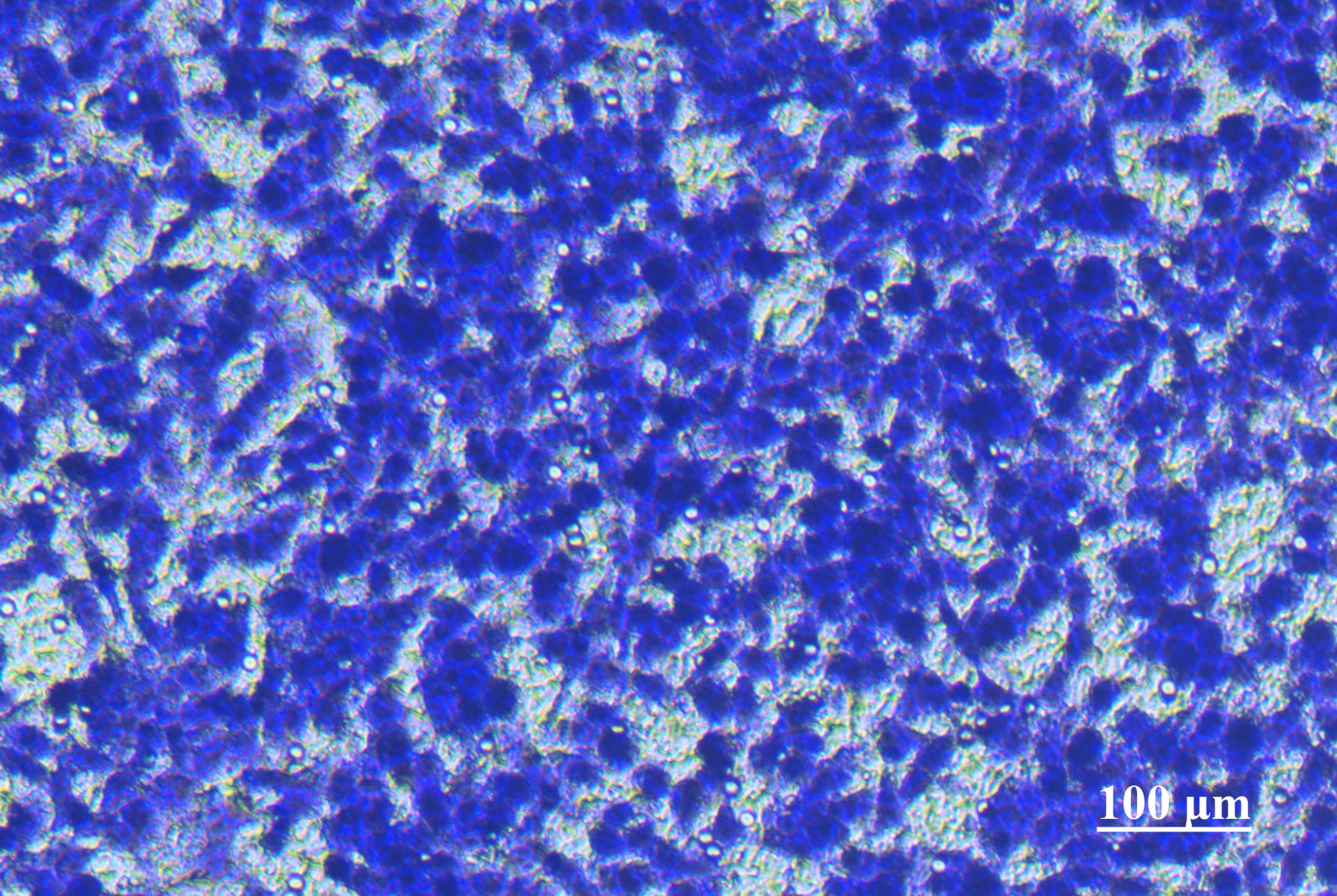

Supplement: Supplementary file 5 — Supplementary Material 5 [file 12885_2024_12140_MOESM5_ESM.zip › Fig.4/4H/U-CH2/IN-4-2-1.jpg]

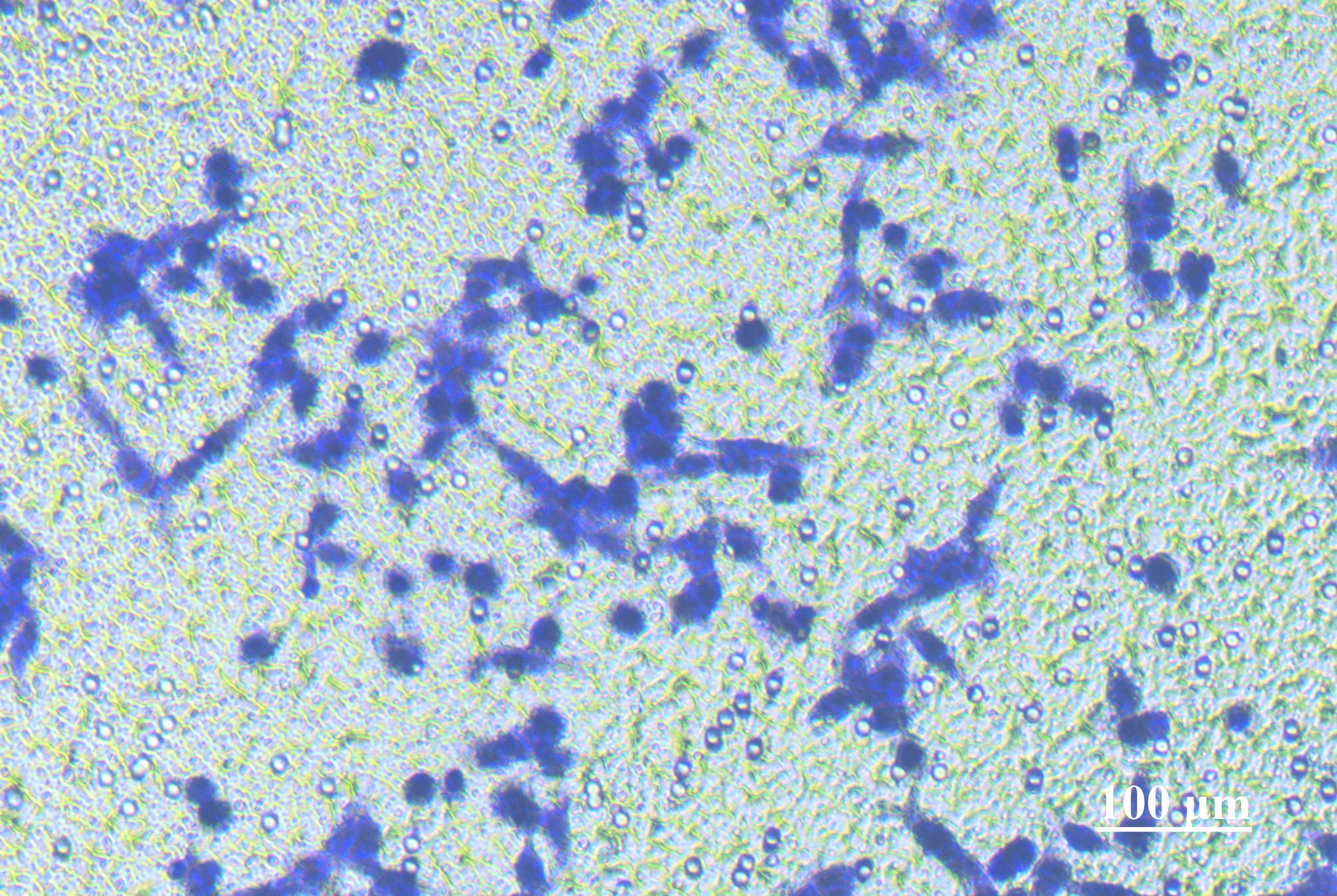

Supplement: Supplementary file 5 — Supplementary Material 5 [file 12885_2024_12140_MOESM5_ESM.zip › Fig.4/4H/U-CH2/IN-4-2-2.jpg]

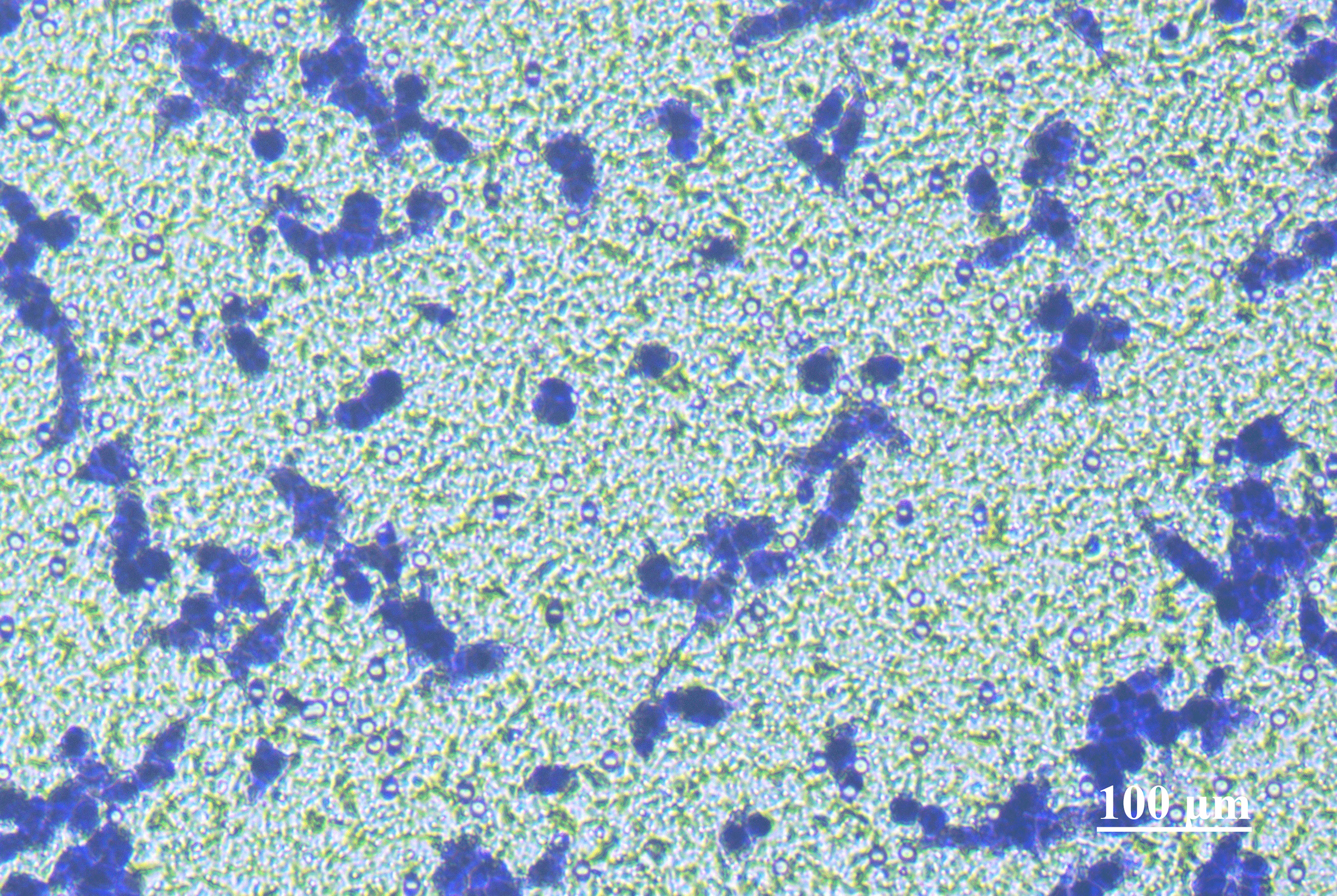

Supplement: Supplementary file 5 — Supplementary Material 5 [file 12885_2024_12140_MOESM5_ESM.zip › Fig.4/4H/U-CH2/IN-4-2-3.jpg]

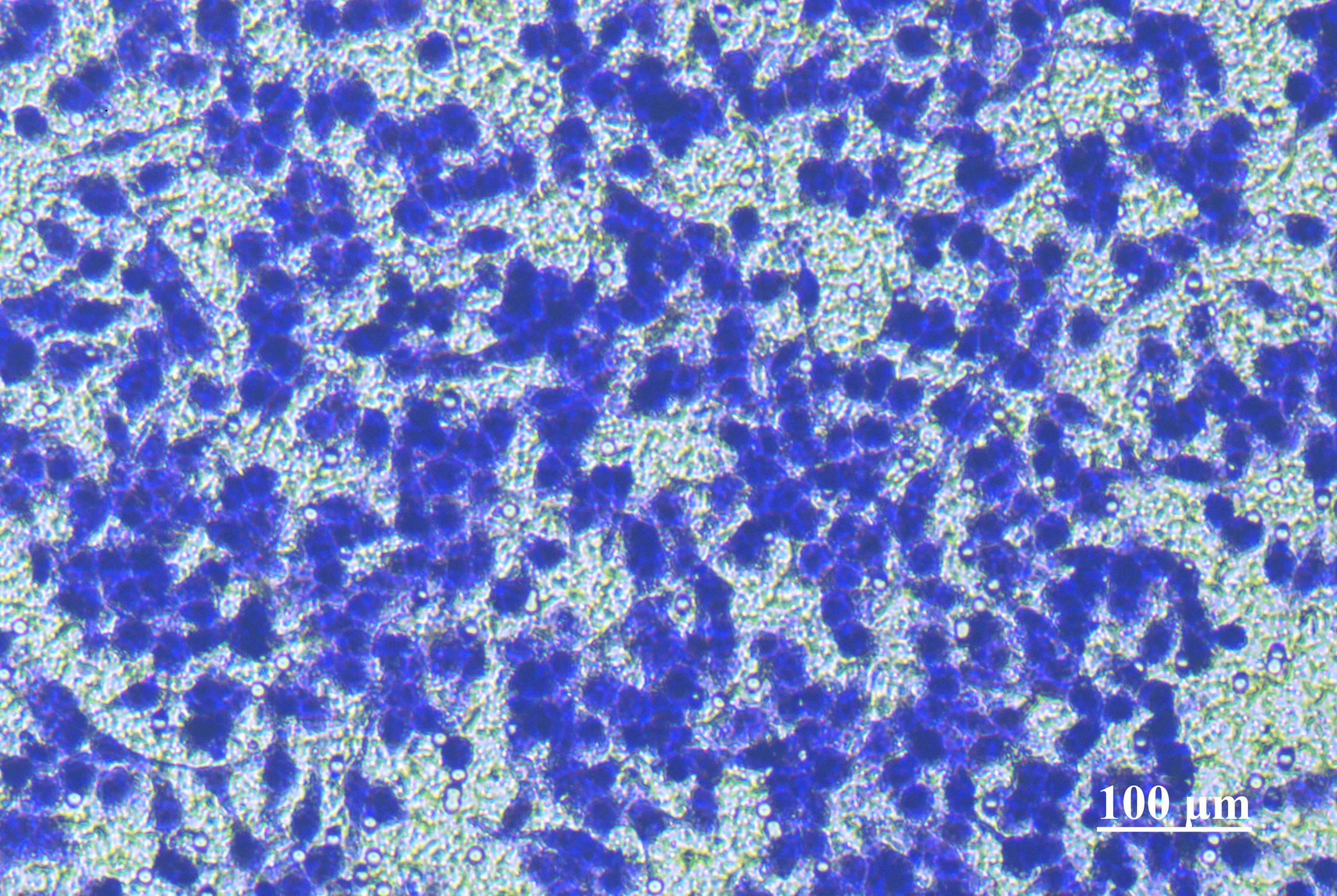

Supplement: Supplementary file 5 — Supplementary Material 5 [file 12885_2024_12140_MOESM5_ESM.zip › Fig.4/4H/U-CH2/IN-4-2-4.jpg]

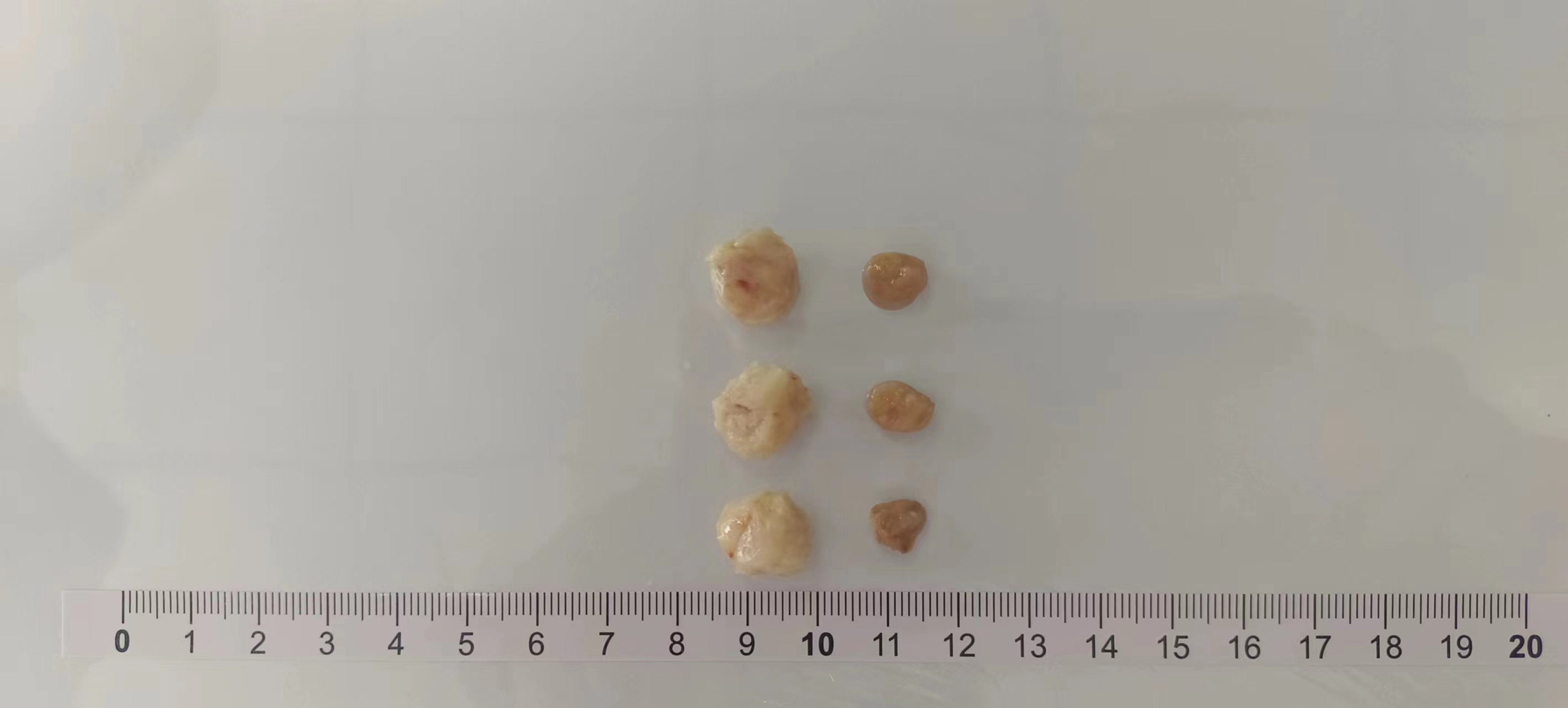

Supplement: Supplementary file 6 — Supplementary Material 6 [file 12885_2024_12140_MOESM6_ESM.zip › Fig.5/5A/5A.jpg]

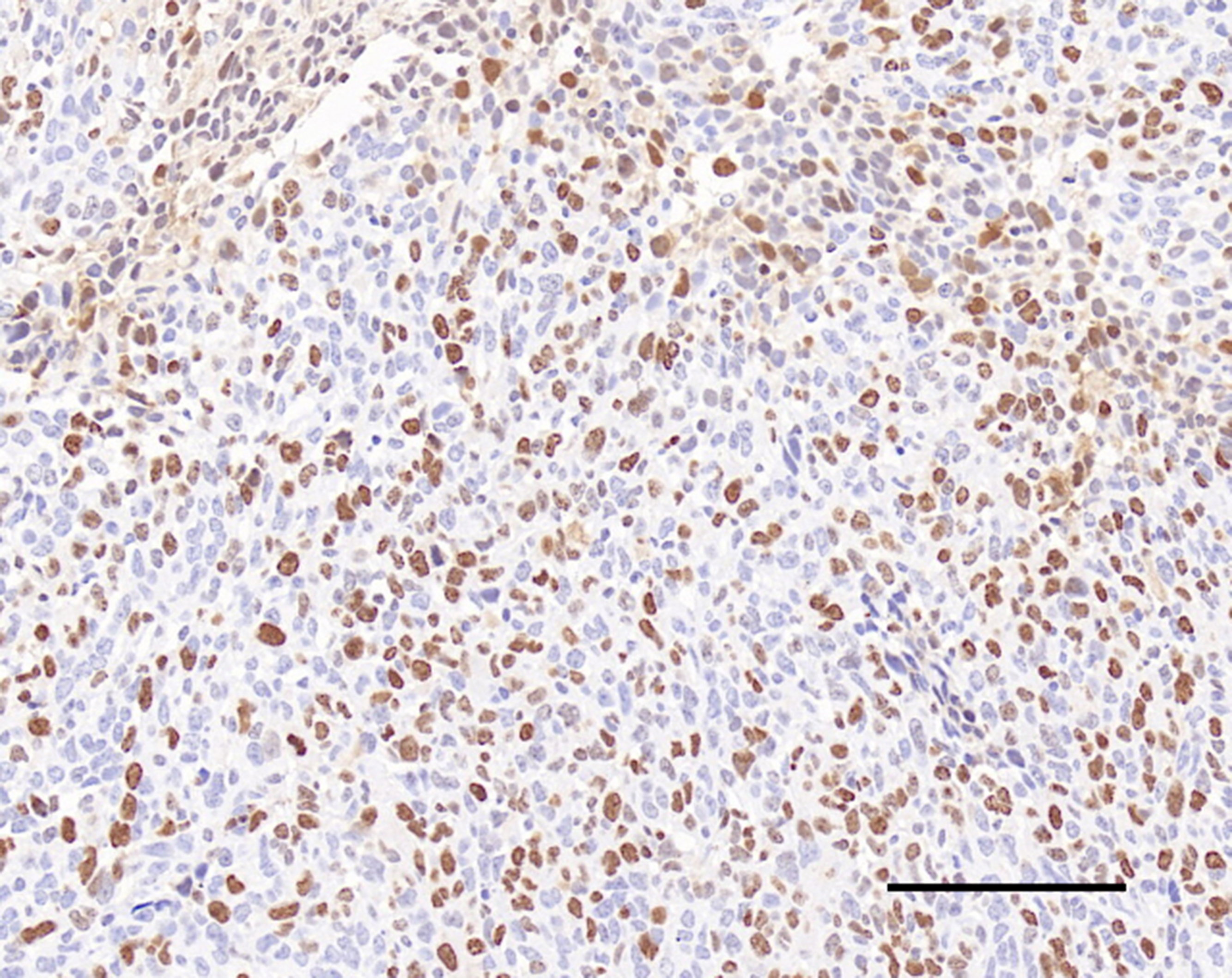

Supplement: Supplementary file 6 — Supplementary Material 6 [file 12885_2024_12140_MOESM6_ESM.zip › Fig.5/5D/1-1.jpg]

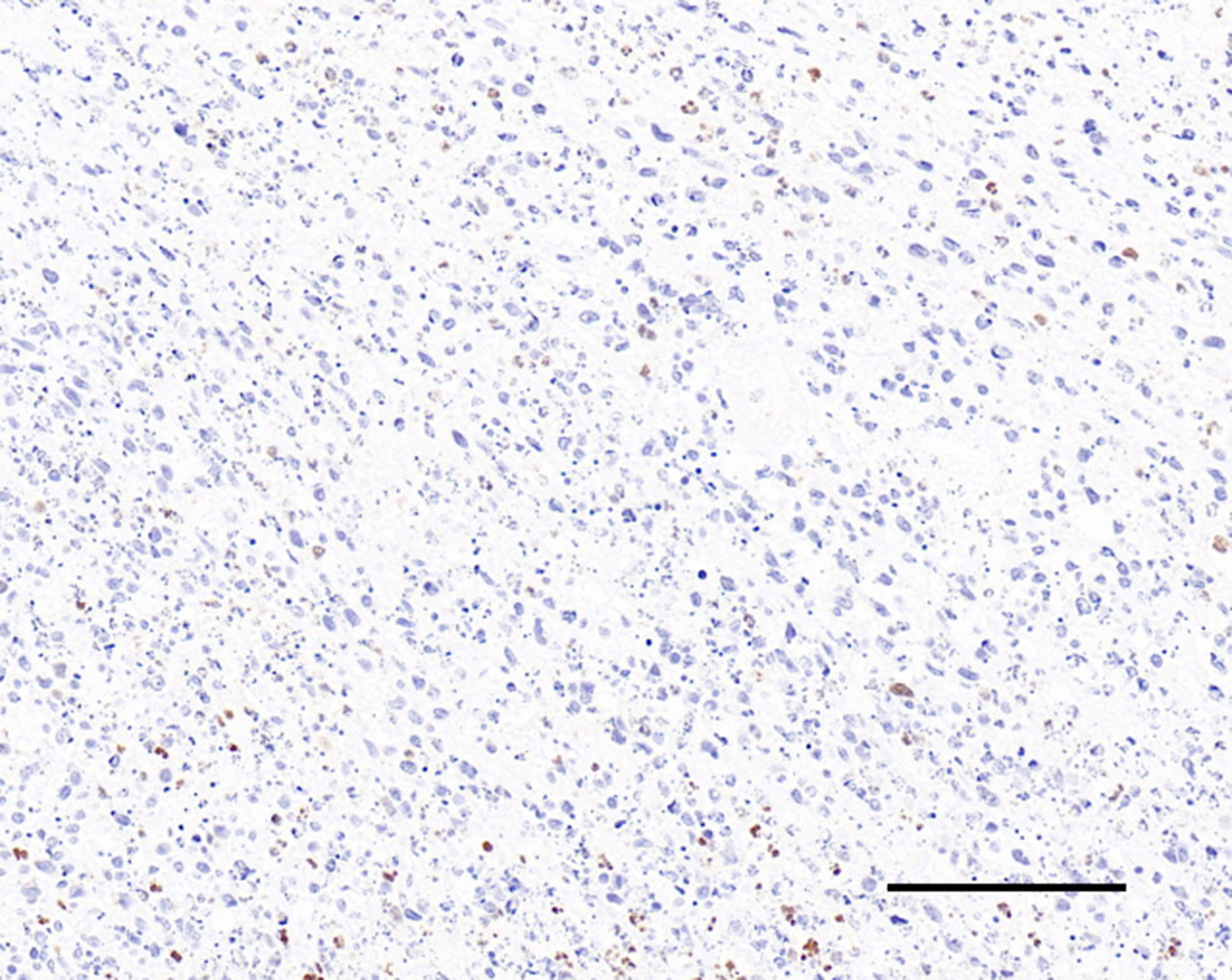

Supplement: Supplementary file 6 — Supplementary Material 6 [file 12885_2024_12140_MOESM6_ESM.zip › Fig.5/5D/1-2.jpg]

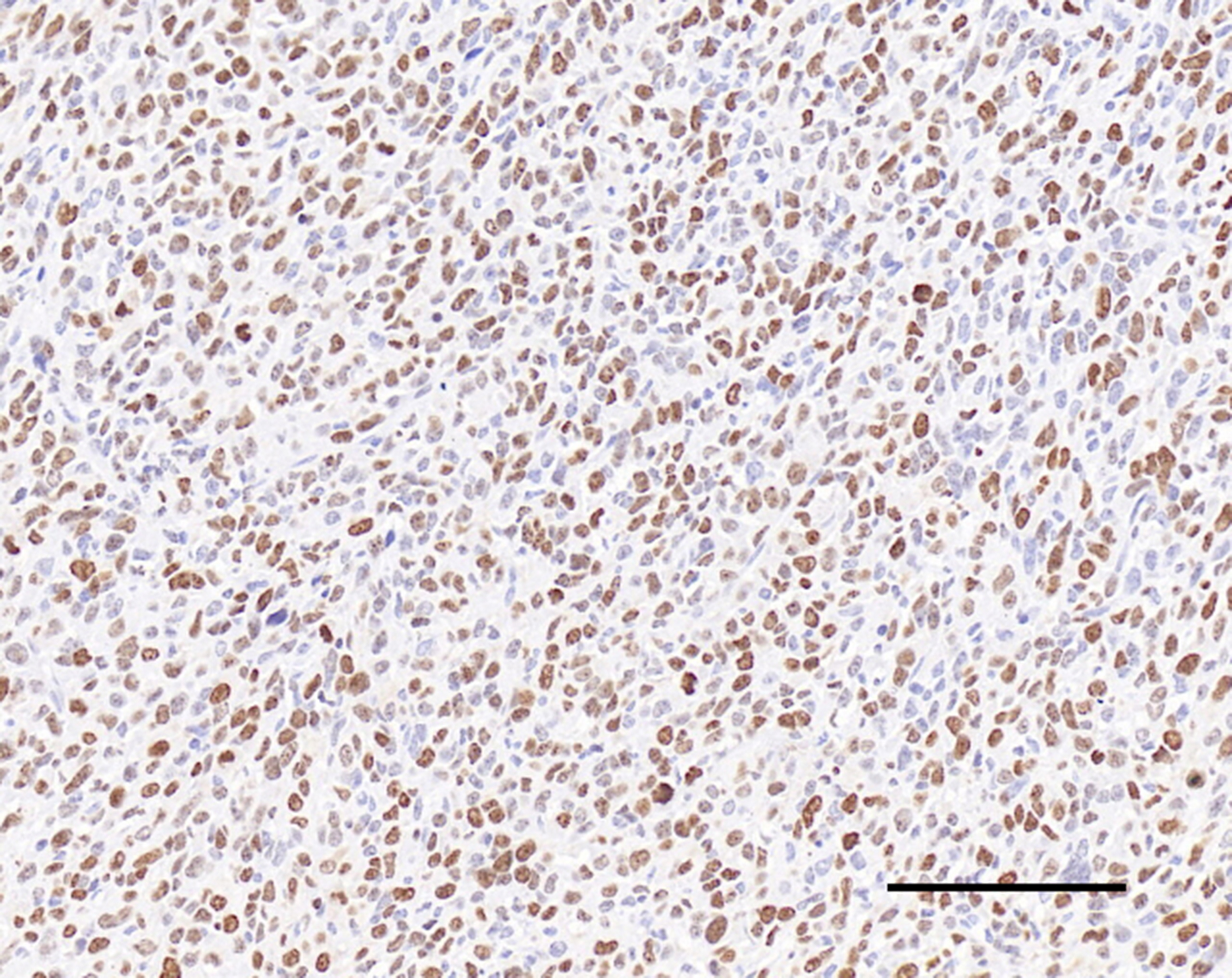

Supplement: Supplementary file 6 — Supplementary Material 6 [file 12885_2024_12140_MOESM6_ESM.zip › Fig.5/5E/2-1.jpg]

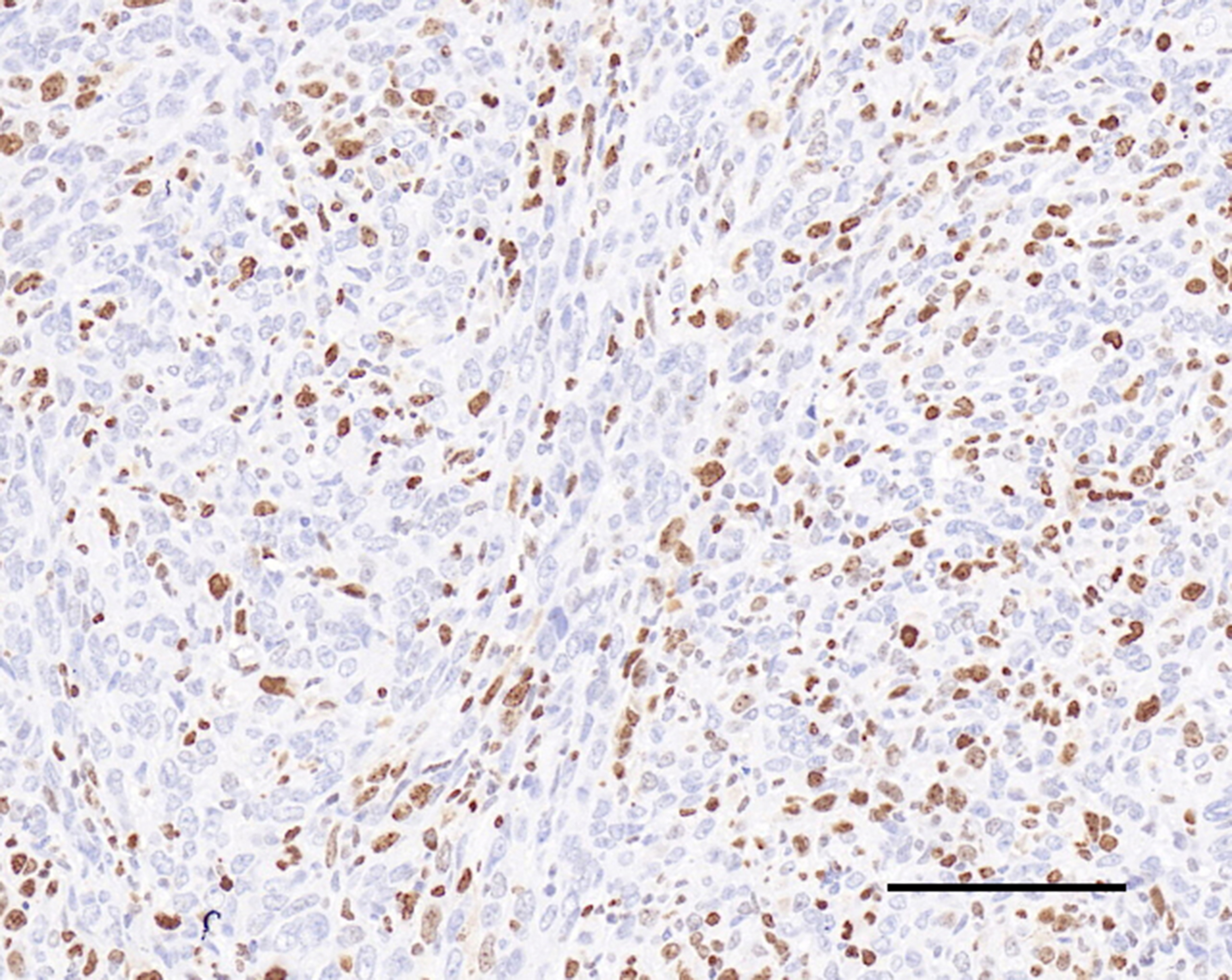

Supplement: Supplementary file 6 — Supplementary Material 6 [file 12885_2024_12140_MOESM6_ESM.zip › Fig.5/5E/2-2.jpg]
